# Composition of Transcription Machinery and Its Crosstalk with Nucleoid-Associated Proteins and Global Transcription Factors

**DOI:** 10.3390/biom11070924

**Published:** 2021-06-22

**Authors:** Georgi Muskhelishvili, Patrick Sobetzko, Sanja Mehandziska, Andrew Travers

**Affiliations:** 1 School of Natural Sciences, Agricultural University of Georgia, David Aghmashenebeli Alley 24, Tbilisi 0159, Georgia; 2 Department of Chromosome Biology, Philipps-Universität Marburg, LOEWE-Zentrum für Synthetische Mikrobiologie, Hans-Meerwein-Straße, 35043 Marburg, Germany; sobetzko@staff.uni-marburg.de; 3 School of Engineering and Science, Campus Ring 1, Jacobs University Bremen, 28759 Bremen, Germany; sanja.mehandziska@zmc.mk; 4 MRC Laboratory of Molecular Biology, Francis Crick Avenue, Cambridge Biomedical Campus, Cambridge CB2 0QH, UK; aat@mrc-lmb.cam.ac.uk; 5 Department of Biochemistry, University of Cambridge, Tennis Court Road, Cambridge CB2 1GA, UK

**Keywords:** RNA polymerase supramolecular complex, metabolic enzymes, nucleoid-associated proteins, regulons and couplons, DNA topology

## Abstract

The coordination of bacterial genomic transcription involves an intricate network of interdependent genes encoding nucleoid-associated proteins (NAPs), DNA topoisomerases, RNA polymerase subunits and modulators of transcription machinery. The central element of this homeostatic regulatory system, integrating the information on cellular physiological state and producing a corresponding transcriptional response, is the multi-subunit RNA polymerase (RNAP) holoenzyme. In this review article, we argue that recent observations revealing DNA topoisomerases and metabolic enzymes associated with RNAP supramolecular complex support the notion of structural coupling between transcription machinery, DNA topology and cellular metabolism as a fundamental device coordinating the spatiotemporal genomic transcription. We analyse the impacts of various combinations of RNAP holoenzymes and global transcriptional regulators such as abundant NAPs, on genomic transcription from this viewpoint, monitoring the spatiotemporal patterns of couplons—overlapping subsets of the regulons of NAPs and RNAP sigma factors. We show that the temporal expression of regulons is by and large, correlated with that of cognate regulatory genes, whereas both the spatial organization and temporal expression of couplons is distinctly impacted by the regulons of NAPs and sigma factors. We propose that the coordination of the growth phase-dependent concentration gradients of global regulators with chromosome configurational dynamics determines the spatiotemporal patterns of genomic expression.

## 1. Introduction

The bacterial transcriptional regulation system is one of the most fascinating of evolutionary devices. It comprises an intricate network of interdependent genes modulating the chromosome dynamics and thus providing an integrated response to changing environmental conditions [1,2,3,4]. The main hallmark of this system (also a stumbling block for exploring it) is its organizational complexity, featuring control mechanisms involving spatiotemporally coordinated communications between its analog and digital components [5,6]. In this system, the unique sequences of individual genes represent discontinuous (i.e., digital) entities expressed as continuous (analog) variables—different species of indistinguishable protein molecules produced at various concentrations, including the abundant DNA binding proteins. The latter in turn determine the occupation pattern of chromosomal DNA binding sites thereby affecting the activity of individual genes/operons by switching them on or off [3]. This interconversion of analog and digital information makes the system organizationally closed, that is, the information flow in the system is circular. The type of genetic control mediated via transcription factors that bind specific, high affinity DNA sites has been dubbed digital control [5,7]. Digital control can be assessed in “effective” (i.e., growth condition-specific) transcript profiles using the electronically compiled transcriptional regulatory network (TRN) as a reference [8]. However, the TRN comprises only a part of communications within the genetic regulation system, namely those between the DNA binding transcription factors (TFs) and their target genes (TGs), which even in the best-studied bacterial model organism *E. coli* comprise only about one third of total genes, while the rest is not known to be under the control of TFs [9]. Interestingly, some bacterial endosymbionts seem to lack the TFs almost entirely [10]. Furthermore, certain transcription processes cannot be readily accounted for on the basis of TF-TG interactions, as for example, the widespread transcriptional read-through by RNAP and production of long transcripts extending across adjacent operons as well as the local, gene orientation-dependent transcriptional effects [11,12]. The nature of the regulation of the genes that are not part of the TRN remained uncertain until the development of high-throughput approaches, which discovered distinct genome-wide patterns of transcriptional responses to changes of the chromosomal DNA topology induced by DNA topoisomerase inhibitors and environmental stress [13,14,15,16,17]. Accordingly, this latter type of genetic control, mediated by a continuous variable—namely, the concerted alterations of DNA superhelical density and composition of abundant chromatin architectural proteins—was dubbed analog control. Importantly, the digital and analog control types were found to counterbalance each other that is, one type can compensate for the deficiency in the other [5]. While the connection between the changes of DNA topology and cellular metabolism has been well documented in earlier studies [18,19,20], recent studies strongly suggest that analog control via DNA topology provides the main mechanism sustaining the metabolic coherence ([21]; but see also [22]).

In contrast to the eukaryotic nucleus where the predominant class of proteins compacting and packaging DNA is the histones, in the bacterial nucleoid a variety of highly abundant NAPs are present some of which are widely distributed such as the members of the HU, Lrp and H-NS families while others, for example FIS, are more restricted in their occurrence. Available data indicate that, during the bacterial growth cycle, the crosstalk between the NAPs and the DNA topoisomerases regulates the overall chromosomal supercoil density homeostatically [1,15,17]. While both the DNA topology as well as the composition and relative abundance of the NAPs vary in a growth phase-dependent manner, the NAPs predominantly recognise local DNA conformations rather than the DNA bases per se, and thus, generally exhibit a wide and quasi-continuous range of DNA sequence-dependent affinity, acting as architectural proteins stabilising a particular configuration of the trajectory of the DNA double helix [23,24,25]. The ordering of DNA structure by NAPs can be considered to be acting at several levels, including twisting and untwisting or DNA bending over short distances, stabilisation of more extensive supercoil-dependent structures and finally, long range organisation of large domains. It is the synthesis of this ordering that impacts the overall pattern of bacterial gene regulation.

## 2. The Intracellular Context

The growth phase-dependent gene expression during the *E. coli* growth cycle is ultimately dependent on energy and resource availability. One question then is how energy availability is globally linked to patterns of, for example, transcriptional regulation. Early data showed that the progression of the growth cycle was accompanied by changes in the internal ionic composition, notably a predominance of K^+^ on nutritional shift-up changing gradually to a predominance of Na^+^, especially in late stationary phase [26]. This picture is likely an over-simplification of the overall compositional changes. For example, rapid accumulation of potassium glutamate is triggered by osmotic shock [27,28], a phenomenon, which concomitantly increases DNA supercoiling levels [18]. Although the levels of potassium glutamate during the growth cycle have not been experimentally determined, *gltP*, the gene for the proton (and hence energy)-dependent glutamate transporter is maximally expressed immediately after nutritional shift-up [29], a situation in which the preferential expression of genes dependent of high levels of DNA superhelicity is strongly favoured [30]. Likewise, *kdpA* and *kdpB*, encoding two components of the major K^+^ transporter complex [31] are primarily expressed during the lag (aka early) phase of the growth cycle in a rich medium [29].

Internal ionic compositional changes in glutamate, K^+^ and Na^+^, because of their differential effects on water potential dependent largely on the Hofmeister series (glutamate > K^+^ > Na^+^) [32], could affect the structures of both proteins and DNA. An increase in water potential is correlated with an increase in the axial twist of helical regions of both proteins [33] and DNA [34,35]. In this context the increased intracellular superhelicity observed during salt shock could simply be a homeostatic response to an increase in DNA twist. Similarly, the extent of compaction of the 30 nm fibre is strongly dependent on the nature of the monovalent cation (K^+^ or Na^+^) [36]. Importantly, promoter selection by the *E. coli* σ^70^ RNA polymerase holoenzyme in vitro is also dependent on ionic strength [37]. Since changes in water potential can, in principle, strongly impact both DNA and protein structure in similar directions, we would expect the functions of both DNA and proteins, especially RNA polymerase, to be tightly integrated during the growth cycle, such that modulators of DNA structure and organisation, such as the NAPs, would be functionally coupled to the operation of the major direct transcriptional regulators, such as polymerase itself and the TFs. In this review, we explore how the extent of this coupling changes with the growth cycle.

## 3. Modulators of RNAP

Whatever the implicated type of transcriptional control, the central element of genetic regulation system is the multi-subunit RNAP holoenzyme, which not only transcribes genes adjusting the cellular metabolism to changing environmental conditions, but also receives numerous signals from the environment by interacting with regulatory proteins and other molecules providing various impacts according to the physiological state of the cell. These include factors involved in transcription initiation, elongation and termination, RNAP secondary channel modulators and small molecules, such as the purine nucleotide derivative alarmones pppGpp and ppGpp that play a key role in stringent response as well as their putative opponent pppApp [38]. Ultimately, all these factors are involved in analog control by modifying and adjusting the RNAP composition and function to DNA topology and chromatin architecture [1,38,39,40].

**Figure 1:**
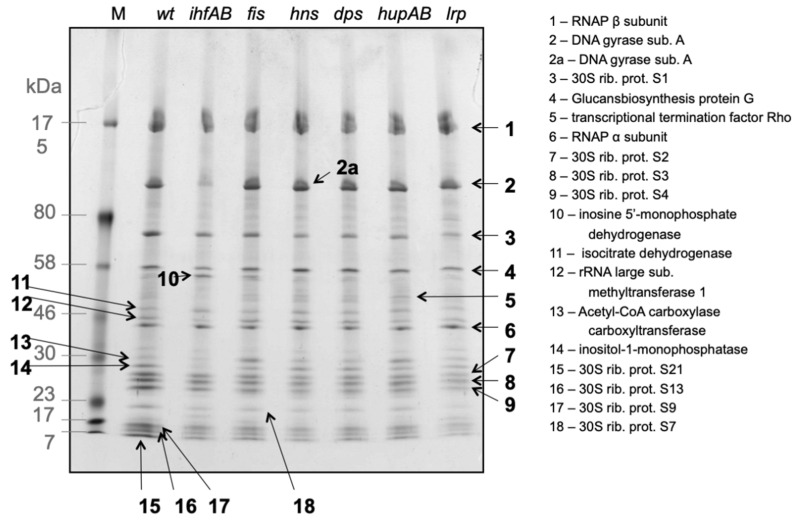
The RNAP supramolecular complex. Coomassie-stained, 2nd dimension SDS-PAGE of RNA polymerase supramolecular complexes isolated from *E. coli* CSH50 wild type cells and NAP deficient mutants (as indicated above the lines). The shown (2nd dimension) SDS-PAGE was preceded by heparin column chromatography and non-denaturing Blue-Native PAGE, from which the RNAP supramolecular complexes were excised and resolved into individual components by denaturing SDS-PAGE. The bands in the gel were further subjected to in-gel trypsin digestion followed by MS or MS-MS analyses [45]. Protein identities are linked to the numbers. Further details of the protein functions and analyses scores are provided in Supplementary Table S1.

Importantly, previous studies using various approaches made it increasingly clear, that in vivo the RNAP holoenzyme can be closely associated with DNA topoisomerases, ribosomal proteins and metabolic enzymes [41,42,43,44,45]. While the composition of such a multiprotein RNAP assembly, designated the RNAP supramolecular complex, critically depends on the applied isolation procedure, it was found to vary with growth phase [43,46] and be alterable by mutations of NAP genes [45] (Figure 1; Supplementary Table S1). Despite variable composition, some of the RNAP-associated proteins such as the ribosomal proteins, DNA gyrase subunits and certain metabolic enzymes (e.g., inosine 5′-monophosphate dehydrogensase—IMPDH), have been identified repeatedly by different approaches. Detected ribosomal proteins most likely reflect the association of the transcribing RNAP with ribosomes in the expressome complex functionally coupling transcription with translation in *E. coli* and *Mycoplasma* but not in Gram positive model bacterium *B. subtilis* and other Firmicutes, where most of the operons are translated without a closely trailing ribosome and where this “runaway transcription” creates different rules for cotranscriptional regulation [47,48]. Interestingly, while about a third of the 30S ribosomal subunit proteins were found to be associated with RNAP supramolecular complex isolated by heparin column chromatography, none of the 50S subunit proteins were detected (Figure 1; Supplementary Table S1), supporting the view that RNAP forms a complex with the 30S ribosomal subunit prior to expressome formation. The enzymes that co-purify with RNA polymerase, including those involved in tRNA processing, nucleotide metabolism, and energy biosynthesis, were proposed to be necessary for optimal transcription rates [43]. Notably, among the RNAP-associated metabolic enzymes, inositol monophosphatase (SuhB) is an integral part of the ribosomal antitermination complex involved in rRNA maturation and ribosome assembly [49]. SuhB both interacts with RNA and competes with C-terminal domain of the RNAP α subunit (αCTD) for binding the antitermination protein NusA [50].

Another RNAP-associated enzyme, inositol monophosphate dehydrogenase (IMPDH), is encoded by *guaB* gene conserved from prokaryotes to humans and catalyzes the NAD^+^-dependent oxidation of IMP to xanthosine monophosphate, thus controlling the guanine nucleotide pool size [51]. Notably, IMPDH binds single-stranded nucleic acids both in vivo and in vitro [52]. In *Drosophila*, IMPDH was shown to bind single-stranded CT-rich DNA, acting as a transcriptional repressor of an evolutionarily conserved gene regulatory network of nucleotide metabolic enzymes and thus, linking the metabolic state to cell proliferation [53]. Also the *E. coli* IMPDH binds CT-rich single-stranded DNA via its Bateman domain and independent of its catalytic function, inhibits adenylate nucleotide biosynthesis [54]. IMPDH thus affects the ATP homeostasis, which in turn could affect DNA gyrase activity and eventually, modulate DNA supercoiling [18,19,55]. Notably, the *E. coli guaB* gene, as well as almost all the nucleotide biosynthesis genes, is activated by increased negative superhelicity [15]. It is conceivable that in the context of RNA polymerase supramolecular complex, the DNA single-strand binding activity of IMPDH plays a role in adjusting the transcription machinery and DNA topology at these supercoiling-dependent gene promoters. In addition, IMPDH has been associated with transcription elongation and translation [56,57,58].

Furthermore, in the context of RNAP supramolecular complex functionally meaningful relationships have been proposed for enzymes involved in the metabolism of lipopolysaccharides [46]. For example, the RNAP-associated periplasmic glucan biosynthesis protein MdoG (Supplementary Table S1) is encoded by *mdoG* (now *opgG*) gene; a constituent of *mdoA* operon the mutations of which influence the levels of stationary phase σ^S^ initiation factor in the cell [59]. Additionally, the association of RNAP with isocitrate dehydrogenase (Supplementary Table S1), an enzyme regulated by phosphorylation/dephosphorylation allowing rapid shifts between the catabolic TCA and the anabolic glyoxalate bypass pathways, which are enriched respectively for genes responding to DNA relaxation and high negative superhelicity [15], is suggestive.

The observed close association of RNAP with metabolic enzymes is consistent with proposed interdependence of cellular metabolism and transcription [3], revealing a new layer of analog control of gene expression via direct communication of RNAP with metabolic enzymes and perhaps, also via indirect effects mediated by changing availability of metabolites, as proposed for replication control [60]. Analog control is conceivable here given that the concentration of both the enzymes and metabolites in the cell is sufficiently high to generate concentration gradients. It is noteworthy, however, that while the composition of RNAP supramolecular complex varies with growth phase [43,46] the relationship between this variation and the growth phase-dependent changes in RNAP sigma factor composition remains largely unexplored.

## 4. Role of DNA Topology and Homeostatic Regulation of Supercoiling Response

Compositional variation in the transcription machinery during the *E. coli* growth cycle is paralleled by variation of spatial distribution of RNAP in the nucleoid, likely determined by the changes of NAP composition, DNA topology and overall nucleoid configuration [61,62]. Notably, while the direct communications of metabolic enzymes with RNAP could relate the extant state of particular physiological function(s) to transcription machinery, the topoisomerases relay the cellular energy charge to the global supercoiling level of chromosomal DNA [18,19,55] and also, facilitate the translocation of RNAP along the DNA template by relieving the supercoiling imbalance resultant from the Liu/Wang twin supercoil domain effect, manifest in accumulation of positive and negative supercoils respectively ahead and behind of the translocating RNAP [63,64]. Furthermore and perhaps most importantly, the superhelical density of the DNA modulates the promoter recognition and transcription initiation by RNAP holoenzyme [1]. Binding of RNAP at the strong stable RNA promoters lead to constraint of writhed microloops storing torque, which subsequently can be utilized for promoter opening via topological writhe to twist transition [65,66]. The RNAP holoenzyme and DNA topology thus act conjointly as major analog factors harnessing and channeling the torque for facilitated promoter opening and transcription initiation [38].

Dynamic changes in DNA supercoiling and NAP composition in vivo determine both how DNA is packaged and how it is accessed for transcription initiation and other DNA transactions [65]. At the same time, changes in DNA superhelical density concertedly alter the expression of the networked genes of NAPs, RNAP sigma initiation factors, transcription elongation and termination factors and RNAP secondary channel modulators [3]. Accordingly, gene expression analyses of the wild type *E. coli* cells and its mutant derivatives lacking the NAPs (either FIS or H-NS, or both) grown under conditions of norfloxacin-induced hypernegative DNA supercoiling (−σ~0.09) and DNA relaxation (−σ~0.03) reveal specific patterns of up-regulated genes (Table 1), consistent with the homeostatic network regulating DNA supercoiling [55,67,68]. DNA hypernegative superhelicity induced by the addition of the TopoIV and gyrase inhibitor norfloxacin to the *E. coli* LZ54 WT strain (and its derivatives lacking the NAPs) encoding norfloxacin-resistant gyrase (i.e., TopoIV is inhibited, whereas gyrase is not), activates the *yrdD* and *parC/parE* genes encoding DNA relaxing topoisomerases. Interestingly, under the same conditions activation of the *crl* gene, the product of which facilitates the assembly of RNAPσ^S^ holoenzyme, is observed [69,70,71]. In addition, the *rpoZ* gene, encoding the RNAP ω subunit, which stabilises the σ^70^ holoenzyme assembly [72], as well as the *rpoE*, *nusG* and *rho* genes respectively encoding RpoE initiation, NusG elongation and Rho termination factors, are up-regulated. Transcription elongation factor NusG enhances the elongation rate [73] and links transcription to translation [74,75] by forming a bridge with ribosomal protein S10 (aka NusE). NusG thus stabilizes the RNAP-ribosome interaction in the expressome complex, albeit dependent on the length of the mRNA spacer between the RNAP and the P site of the ribosome active-center [76]. However, the RNAP-ribosome contact likely precedes the association of NusG with the complex, suggesting that recruitment of NusG depends on translation [77]. The termination factor Rho can resolve clashes between transcription and replication machineries [78], is crucial for maintaining the transcriptional boundaries in the genome and, being supported by NusG and NusA, exerts a silencing effect on transcription of AT-rich DNA [79] which may undergo undesirable spontaneous unwinding under conditions of high negative superhelicity.

Importantly, Rho is associated with RNAP throughout the transcription cycle allosterically trapping the transcription complex [74,80,81] and preventing the formation of deleterious R-loops (a loop formed by single-stranded RNA base-paired with one strand of duplex DNA with the complementary DNA strand being displaced) at supercoiling-dependent stable RNA operons [82], as well as precluding antisense transcription [83] and transcriptional read-through [79], potentially increased under conditions of DNA hypernegative supercoiling. Concomitant activation of sigma *rpoE* and anti-sigma *rseAB* genes by hyperenegative DNA supercoiling (Table 1) suggests an induction of envelope stress under hypernegative supercoiling regimen [84] and supports the notion that the σ and anti-σ factors are often co-transcribed to ensure the maintenance of stoichiometric levels [85].

Again in keeping with homeostatic control of DNA supercoiling, DNA relaxation induced by addition of norfloxacin to LZ41WT strain (and its derivatives lacking the NAPs), encoding norfloxacin-resistant TopoIV (i.e., gyrase is inhibited, whereas TopoIV is not), activates the *gyrA* and *gyrB* genes encoding the DNA gyrase subunits. DNA relaxation also activates the genes encoding the sigma factors RpoD, RpoH and the secondary channel modulator GreA (Table 1). Activation of this latter is consistent with its role in ppGpp-DksA regulatory subnetwork [40,86] and might counteract the down-regulation of ribosomal RNA promoters under conditions of DNA relaxation [87,88]. The *E. coli rpoH* gene encoding the heat shock σ^32^ (aka σ^H^) factor is sensitive to changes of DNA supercoiling, and DNA relaxation induced by novobiocin treatment of *B. subtilis*, was shown to decrease transcription from both the ribosomal RNA promoters and the promoters dependent on alternative sigma factors including σ^H^ [89,90]. The RNAP σ^70^ subunit encoded by *rpoD* gene confers preference for highly negatively supercoiled templates [91] and its overexpression can increase the global DNA negative superhelicity [39], suggesting a compensatory activation of *rpoD* expression counteracting the norfloxacin-induced DNA relaxation especially in LZ41 cells lacking the NAPs and hence, their buffering effects due to constraint of negative supercoils. Among the genes encoding the NAPs, *fis*, *lrp*, *hupB*, *ihfA* and *stpA* are activated by hypernegative supercoiling, while *ihfB*, *hupA*, and the global TFs *crp* and *fnr* are activated by DNA relaxation (Table 1). The *fis* promoter depends on high negative superhelicity for optimal transcription [92], whereas binding of FIS at phased sites in upstream activating sequences of stable RNA (ribosomal and transfer RNA) promoters stabilises tightly bent loops, acting as a topological “homeostat” rescuing these supercoiling-dependent promoters from inactivation on deviations from optimal superhelicity [65,87]. The *crp* gene is transcribed from two promoters showing different dependence on DNA topology, one of which is derepressed on transition to stationary phase [93] associated with DNA relaxation, whereas the *fnr* gene is negatively autoregulated by Fnr protein which is activated under anaerobic conditions [94] associated with increased levels of DNA superhelicity. Constraint of DNA supercoils by HU is required for balanced transcription of the bacterial genome [61,95] and the observed activation of *hupA*, as opposed to *hupB*, by DNA relaxation can also be explained by a homeostatic mechanism, since the HUα homodimer, but not the HUβ homodimer, efficiently constrains negative superhelicity and thus, could counteract the global relaxation of DNA [96]. Overall, the changing activities of genes featured in Table 1, mostly constituting the homeostatic network regulating the chromosomal DNA superhelicity [1,3], likely reflect the integrated adaptive response of this overarching network to norfloxacin-induced changes in global DNA supercoiling and its modulation in cells lacking FIS and H-NS. It is conceivable that this adaptive integration occurs, at least in part, at the level of direct communications in the context of RNAP supramolecular complex.

## 5. Interdependence of the Network Elements

**Figure 2:**
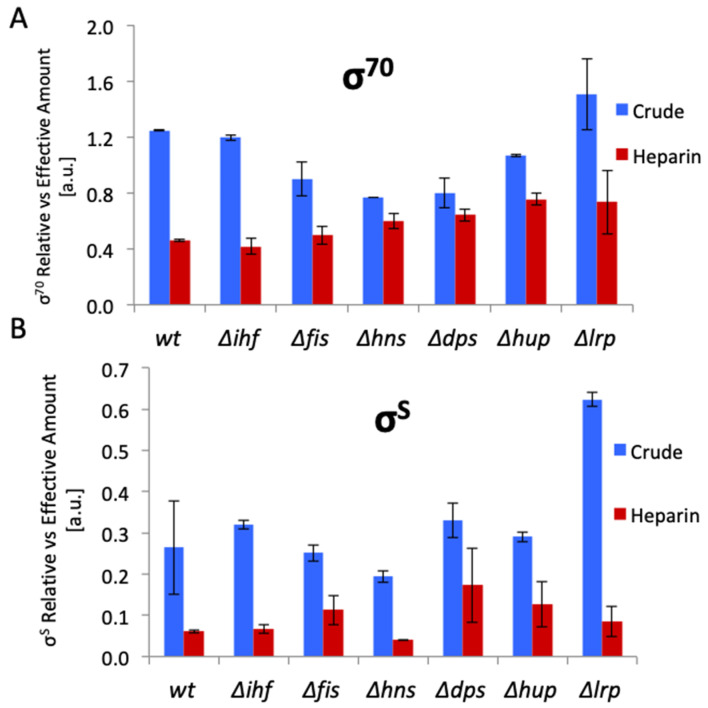
Variation of the σ^70^ and σ^S^ factor levels in *E. coli* strains with mutated NAP genes. Graphical representation of quantitative Western blots of RNA polymerase subunits detected in crude and heparin column-purified protein extracts of *E. coli* CSH50 wild type (wt), Δ*ihfAB*, Δ*fis*, Δ*hns*, Δ*dps*, Δ*hupAB* and Δ*lrp* mutant cells during exponential growth phase (OD_600_ = 0.5) using mouse monoclonal antibodies raised against RpoD (σ^70^) and RpoS (σ^S^) and normalized against RpoC (β’). (**A**). Comparison of the relative (crude cell extract) to effective (heparin protein extract) amount of RpoD (σ^70^) (**B**). Comparison of the relative (crude cell extract) to effective (heparin protein extract) amount of RpoS (σ^S^). Note that in crude extracts the normalized levels of RpoS constitute about one third of those of RpoD. Bars indicate the standard errors of two biological replicates. *E. coli* CSH50 wild type and isogenic NAP mutant strains were grown in 3l of dYT medium (1.6% tryptone 1% yeast extract, 0.05% NaCl) in BIOSTAT Bplus fermentor Sartorius AB, Göttingen, Germany). Conditions were set to 37 °C, pH 7.5, 500 rpm, and pO2 of 100% at the start of the growth, and carefully controlled throughout the growth. For more details see “Experimental procedure to Figure 2” in the Supplementary data file.

**Figure 3:**
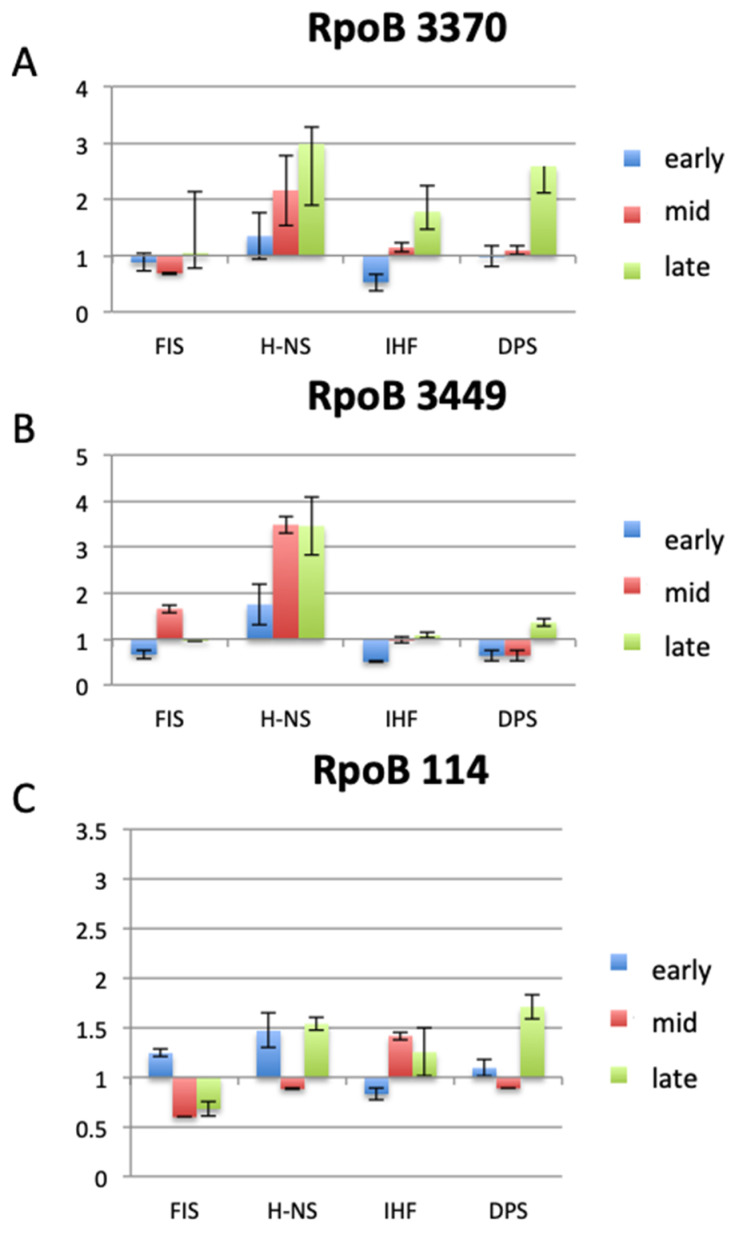
Variation of the NAP levels in *E. coli* CSH50 cells carrying mutations in the beta subunit of RNAP (“stringent” RNAP mutants; described in [97]), during the various growth stages. Western blots of FIS, H-NS, IHF and Dps proteins in crude protein extracts of: (**A**) rpoB3370 (T563P), (**B**) rpoB3449 (D532A) and (**C**) rpoB114 (S531F) strains analyzed during early (exponential), middle (transition to stationary) and late (stationary) growth phases. Overnight cultures (after 12 h of growth in a shaker at 37 °C) were inoculated in rich dYT medium at O.D_600_ of 0.1. The cultures were grown in a fermenter under constant pH 7.4 and high aeration (5 L air per min) at 37 °C. The cells were harvested during exponential phase at O.D_600_ of 0.5 (early), at O.D_600_ of 2.0 during transition to stationary phase (mid) and at O.D_600_ of 4.0 in stationary phase (late). The relative NAP amounts in the “stringent” RNAP mutants were normalized to those in the wild type cells. NAP proteins were detected using mouse monoclonal antibodies raised against FIS, H-NS, IHF and Dps. Bars indicate the standard errors of two biological replicates (for experimental details, see the appended Master Thesis of Steffi Jimmy in the Supplementary data file).

Since the genes encoding DNA topoisomerases, NAPs and transcription machinery components are interconnected in a homeostatic network [1,3], the mutations in RNAP genes can affect the expression of genes encoding the NAPs and vice versa. In *E. coli* strains with mutated NAP genes, the σ^70^ and σ^S^ factor levels vary distinctly with the background—as evident both in crude cell extracts and in heparin column purified preparations of RNAP (Figure 2). This suggests that the NAPs modulate not only the expression levels of sigma factors, but also the sigma factor composition of the holoenzyme. In turn, in extracts of cells carrying mutations in the beta subunit of RNAP (the so-called “stringent” RNAP mutants mimicking the effect of the stringent response regulator ppGpp) [97], the NAP levels are correspondingly altered during bacterial growth (Figure 3). Furthermore, deletion of *rpoZ* gene encoding the RNAP ω subunit increases both the RNAP σ^S^/σ^70^ ratio in the cells and the RpoS impact in the transcript profile, concomitantly modulating the impacts of the NAPs and global TFs and favoring transcription of Rel genes responding to DNA relaxation [39]. Conversely, a decrease of the RNAP σ^S^/σ^70^ ratio in *rpoZ* cells induced by overexpression of RpoD, increases the impact of the latter on the transcript profile and again, modulates the impacts of the NAPs and global TFs (Table 2) favoring transcription of Hyp genes responding to high levels of negative superhelicity [39]. These σ factor-dependent changes in regulatory impacts of the NAPs and global TFs are reflected in up-regulation of corresponding regulons and overlapping subsets thereof—the couplons (Table 2; the organization of couplons will be enlarged upon below).

**Figure 4:**
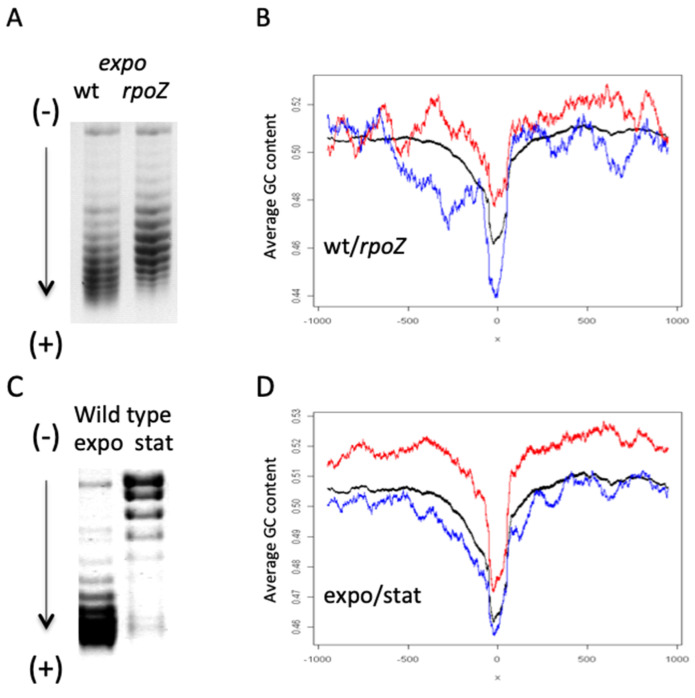
Relationship between DNA topology and the thermodynamic stability of transcribed sequences. (**A**). High-resolution agarose gel-electrophoresis of DNA plasmids isolated from exponentially growing wild-type and *rpoZ* mutant cells. More negatively supercoiled topoisomers migrate faster in the gel. Note the relaxation of DNA in *rpoZ* mutant. (**B**). Comparison of the DNA thermodynamic stability of promoter sequence context in wild type and *rpoZ* mutant cells. Position of transcription start site (TSS) is indicated by 0. Average DNA GC-content of the promoter sequence context for all genes (black curve). Average DNA GC-content of genes up-regulated in wild-type and *rpoZ* mutant cells during exponential phase is indicated by red and blue curves, respectively (data from [39]). (**C**). High-resolution agarose gel-electrophoresis of plasmids isolated from exponentially growing (expo) and stationary (stat) *E. coli* CSH50 cells. Note the relaxation of DNA in stationary phase. (**D**). Comparison of the DNA thermodynamic stability of promoter sequence context in exponentially growing (expo) and stationary (stat) *E. coli* CSH50 cells. The TSS position is indicated by 0. Average DNA GC-content of the promoter sequence context for all genes (black curve). Average DNA GC-content of genes up-regulated in exponentially growing and stationary cells is indicated by red and blue curves, respectively.

Importantly, the increase of the RNAP σ^S^/σ^70^ ratio in *rpoZ* cells where DNA is relaxed, coordinately changes both the global DNA topology and the average thermodynamic stability (GC-content) of the transcribed sequences, indicating that under conditions of DNA relaxation, sequences with lower GC-content are utilized preferentially (Figure 4A,B). This finding is in keeping with previous observations [13]. Corresponding decrease in GC-content of transcribed sequences is observed on transition of cells from exponential growth to stationary phase (Figure 4C,D), characterized by transition from the predominance of RNAPσ^70^ holoenzyme to that of RNAPσ^S^ and from high to low levels of global negative superhelicity, respectively [98]. It is noteworthy, that the more and the less GC-rich sequences are respectively enriched around the origin (OriC) and terminus (Ter) of chromosomal replication, such that ultimately, the genomic sequence organization underpins the sequential transcription of chromosomal ends [30].

Taken together, all these observations support the notion of structural coupling between DNA topology, NAP-dependent chromatin architecture and the transcription machinery composition as a fundamental device coordinating the spatiotemporal genomic transcription [3,38,39]. Understanding of this coordinating effect can be facilitated by analyses of the joint impacts of the NAPs and transcription machinery components on genomic transcription.

## 6. Spatiotemporal Organization of Transcription in Genome

Previous explorations of chromosomal gene organisation revealed the gene copy number effects on gene expression, with higher expression levels characteristic of locations close to the chromosomal replication origin due to multiple rounds of replication initiation [99,100]. These positional effects were interpreted as basic differences in the “expressivity” potential of different chromosomal locations, rather than unique features of translocated genes. Other studies proposed that organization of genes in bacterial genome is explained by selective pressure driving the clustering of essential genes on the leading strand and thus, suggested that gene essentiality, not expressivity, is the determinative factor of chromosomal gene organisation. Still another explanation of spatial gene clustering did not take into account their function but rather their persistence, i.e., their presence either in the majority, or in the minority of the organisms [101,102]. However, pivotal for understanding the relationship between chromosomal gene organization and gene activity was the discovery of coupling between the highly conserved spatial order of major regulatory genes along the chromosomal OriC-Ter axis and the temporal order of their expression during the bacterial growth cycle (i.e., during the period from inoculation of the “overnight” culture in fresh growth medium through the exponential growth to stationary phase) [10,30,103,104]. In other words, it became apparent that the major factor organizing the important regulatory genes spatially along the OriC-Ter axis of the chromosome is the temporal requirement of their products during the bacterial growth cycle. Furthermore, this temporal expression pattern was linked to a putative growth phase-dependent OriC-Ter gradient of negative superhelicity thus coupling the gene expression to the metabolic state of the cell. So far, the existence of this superhelicity gradient could not be experimentally demonstrated. However, the importance of gene order along the chromosomal OriC-Ter axis has been supported by genetic studies demonstrating that chromosomal position shifts of regulatory genes lead to rewiring of the genetic network as well as alterations of both the growth phase-dependent DNA supercoil dynamics and the bacterial phenotype [105,106,107]. Furthermore, the chromosomal gene order appears to underlie the spatial organization of function in genome. In particular, it was observed that anabolic genes are enriched around the OriC-end and catabolic genes around the Ter-end of the *E. coli* chromosome [108]. Since this spatial separation of function was found associated with respectively high and low thermodynamic stability of the DNA at the chromosomal OriC and Ter ends [30,109], it revealed a general strategy underlying the structural-functional organization of the bacterial genomes [108] meaning that at the global level of the physical chromosome the genic ‘typography’ is integrated with the genomic ‘topography’ in the primary sequence organisation of the DNA molecule. Additional relevant features of genomic spatial organization are manifest in the emergence of sub-chromosomal domains eliciting peculiar patterns of gene expression, denoted as coherent domains of transcription (CODOs) [110]. Under different growth conditions various constellations of CODOs were observed both in commensal *E. coli* [30] and in plant pathogen *D. dadantii* [16,111,112]. The CODOs harbor functionally linked genes, the coordinated expression of which is related to particular physicochemical and structural-dynamical properties of their encoding DNA sequences [16,30] and is distinctly impacted by DNA topology-modulating effects of the NAPs [16,111,112].

## 7. Regulons and Couplons

As already mentioned above, the abundant NAPs bind the chromosomal DNA sites in a quasi-continuous mode with affinities differing by three orders of magnitude, affecting the supercoil dynamics of the chromosome and thus contributing to analog control of gene expression. However, numerous target genes have been identified that are regulated by NAPs directly via specific, high-affinity DNA binding sites and therefore, the NAPs are featuring as hubs in the electronically compiled digital TRN [8]. Together, such directly regulated gene classes constitute the TRN regulons, attributed to a particular NAP or a global transcription factor, such as e.g., Catabolite Repressor Protein (CRP) or Fumarate and Nitrate Reduction Regulator (Fnr). Likewise, the various sigma factors of RNA polymerase, having distinct DNA binding specificities and directing the holoenzyme to disparate gene promoters, also establish their cognate regulons [85,113]. The overlaps between the regulons of a particular NAP and a particular sigma factor make it possible to reveal sets of genes under the control of both regulators and thus, assess the coordinated impacts of the transcription machinery of particular composition (i.e., RNAP associated with a certain sigma factor) and a particular NAP (or a global TF, such as e.g., CRP and Fnr) on genomic transcription. Groups of genes regulated conjointly by a particular couple of RNAP holoenzyme (sigma factor) and NAP (or a global TF), are dubbed couplons [3]. Couplons were found to harbor functionally related genes that could be activated or repressed relatively independent of parent regulons, thus featuring as genuine entities reflecting the modular organization of the genetic regulation system. There are couplons with distinct functional properties as well as couplons sharing functional properties, such that combinations of couplons with different sets of genes can support similar functions. However, the vast majority of couplons inherit distinct local functions [3,39]. Notably, while both the NAPs and sigma factors are produced over a range of concentrations and represent analog components of the genetic regulation system, the conversion of analog information into the digital (punctuation, as it were) is achieved by intersections of their concentration gradients, revealing unique genes under the control of a particular couple of NAP (or TF) and sigma factor.

## 8. Spatial Organization of Regulons and Couplons

Given the revealed correspondence between the chromosomal order and temporal expression of genes during the passage of cells from early to exponential to stationary phase, how are the spatial organisation and the temporal expression of genes comprised in regulons and couplons related? The genomic targets of both the NAPs and the RNAP sigma factors demonstrate a peculiar spatial organization [103]. For example, the regulons of RpoD (major σ^70^ factor, predominating during active growth and transcribing the bulk of genes) and RpoS (stationary phase or stress σ^S^ factor predominating on cessation of growth) demonstrate a conspicuous opposite bias of spatial organization, being enriched respectively at the Ori and Ter ends of the chromosome (Supplementary Figure S1). Also the RpoH (heat shock sigma factor) and RpoE (extracytoplasmic sigma factor) regulons show a bias towards the OriC end, whereas RpoN (the nitrogen limitation sigma factor) shows an opposite bias on the left and right replichores. Similarly, the regulons of the NAPs and global TFs, CRP and Fnr, show distinct spatial patterns and all, perhaps except those of Fnr and IHF, show a relative enrichment in the OriC half of the chromosome (Supplementary Figure S2).

**Figure 5:**
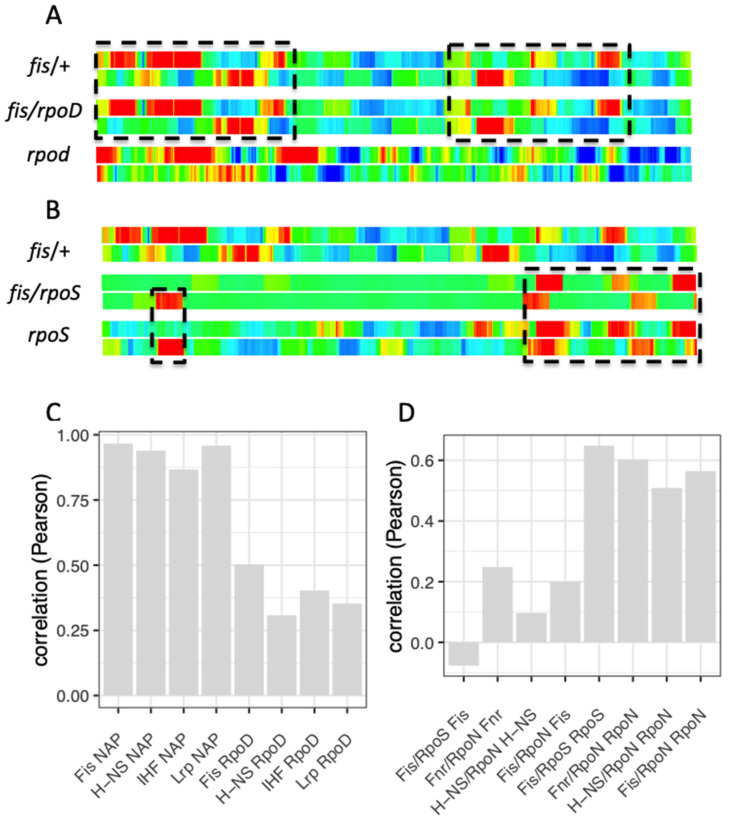
Correlation between the genomic organization of regulons and couplons. (**A**). Genomic organisation of the FIS and RpoD regulons and FIS/RpoD couplons. The circular chromosome is divided into the right (top) and left (bottom) replichores. The origin of replication is situated on the left, the terminus on the right. The red and blue colors respectively indicate a significant enrichment (Z score > 2) and depletion (Z score < −2) in relative frequency of the regulon or FIS/RpoD couplon genes. Note that the pattern of FIS/RpoD couplons is largely coinciding with that of the FIS regulon (dashed box). (**B**). Genomic organisation of the FIS and RpoS regulons and FIS/RpoS couplons. Note that the pattern of FIS/RpoS couplons largely coincides with that of the RpoS regulon (dashed box). Enrichment of regulon genes was determined by comparison of the number of regulon genes within the 100 kb window with the number of regulon genes within random samples with the same number of genes. Z-scores were derived from 10.000 random samples. (**C**). Pearson correlations (ordinate) between the genomic organisation of NAP/RpoD couplons and cognate NAP regulons (indicated on abscissa, the first four columns in the graph. FIS NAP means FIS/RpoD couplons compared to FIS regulon, H-NS NAP means H-NS/RpoD couplons compared to H-NS regulon and so on); the last four columns in the graph show Pearson correlations between the genomic organisation of NAP/RpoD couplons and RpoD regulon (as indicated on abscissa: FIS RpoD means FIS/RpoD couplons compared to RpoD regulon, H-NS RpoD means H-NS/RpoD couplons compared to RpoD regulon and so on); Note the high positive correlation between NAP/RpoD couplons and NAP regulons compared to that between NAP/RpoD couplons and RpoD regulon. (**D**). Pearson correlations (ordinate) between the genomic organization of NAP/ASF couplons and NAP regulons (the first four columns in the graph); Pearson correlations between NAP/ASF couplons and ASF regulons (the last four columns in the graph). Note the high positive correlation between NAP/ASF couplons and ASF regulons. Correlation of the spatial distribution was determined by Pearson correlation of the Z-scores (indicated by rainbow colors along the chromosome in A and B and in Supplementary Figures S3–S5).

Interestingly, spatial organization of couplons reveals an apparent σ factor-dependent peculiarity. For example, genomic distributions of all the couplons of NAPs involving the major σ^70^ factor (RpoD), positively correlate with the spatial pattern of the regulons of NAPs rather than with that of RpoD (Figure 5A,C; Supplementary Figures S3 and S4). In contrast, the spatial patterns of couplons of the NAPs involving alternative sigma factors (ASFs, meaning all the sigma factors except the ‘house-keeping’ sigma RpoD) are positively correlated to the patterns of ASF regulons (Figure 5B,D; Supplementary Figure S5). The difference between the spatial organization of the major RpoD and ASF couplons is perhaps unsurprising, given that the vegetative RNAPσ^70^ holoenzyme binds the vast majority of genomic promoters, whereas the holoenzymes assembled with ASFs which govern more focused functions, bind only subsets thereof [113]. Since the observed biases in the impacts of regulons on spatial organization of couplons are conspicuous, the pertinent question is how these impacts are related to the temporal expression of couplons.

## 9. Temporal Expression Patterns of Regulons and Couplons

**Figure 6:**
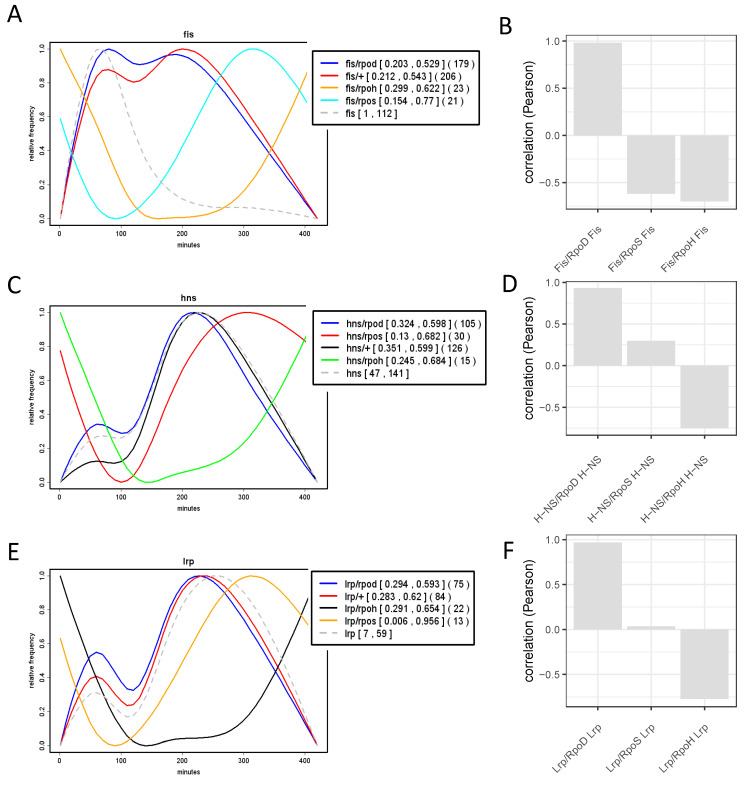
Correlation between the temporal expression of NAP regulons and couplons. (**A**). Growth phase-dependent expression of the FIS regulon, FIS/RpoD and FIS/ASF couplons. The colored curves were computed as average patterns of all the regulon/couplon genes and normalized to 1.0 (for details see legend to Supplementary Figure S6). Abscissa—time in minutes after inoculation of cells in fresh growth medium; ordinate—relative frequency of each class of genes as indicated in the inset. (**B**). Pearson correlations (ordinate) between the temporal expression of FIS regulon, FIS/RpoD couplons and FIS/ASF couplons (FIS/RpoS and FIS/RpoH, as indicated on abscissa). (**C**,**D**). The same as in (**A**,**B**), but for H-NS regulon and couplons. (**E**,**F**), the same as in (**A**,**B**) but for Lrp regulon and couplons. Note the high positive correlation between the temporal expression of NAP regulons and NAP/RpoD couplons but not the NAP/ASF couplons. Plots **A**, **C** and **E**, data from [29]. The expression patterns of *fis*, *hns* and *lrp* genes (in **A**, **C** and **E** respectively) are indicated by grey dashed lines. fis/+, hns/+ and lrp/+ respectively indicate the FIS, H-NS and Lrp regulons. Numbers in squared brackets indicate the minimum and maximum of the regulon/couplon before normalization to [0;1]. Note, that these numbers are averages of the regulon/couplon genes already normaized individually to [0;1]. Numbers in round brackets indicate the number of genes involved in the respective regulon or couplon. The *Escherichia coli* CSH50 overnight (16 h) cultures were inoculated at an initial OD600 of 0.1 in rich double yeast-tryptone (dYT) medium and grown in a fermenter under constant pH 7.4 and high aeration (5 L air per min) at 37 °C for 7 h. Samples for RNA-seq were taken at 1, 2, 3, 5 and 7 h after inoculation and immediately dissolved in ice-cold ethanol–phenol (5% phenol) solution to prevent mRNA degradation. RNA was extracted using the RNeasy Mini kit (Qiagen, Hilden Germany) and treated with Turbo DNase (Life Technologies, Carlsbad USA). Subsequent rRNA depletion was carried out using the MicrobExpress kit (Life Technologies) and 0.5 μg of enriched mRNA of each sample were subjected to RNA-seq (Illumina HiSeq 2000).

**Figure 7:**
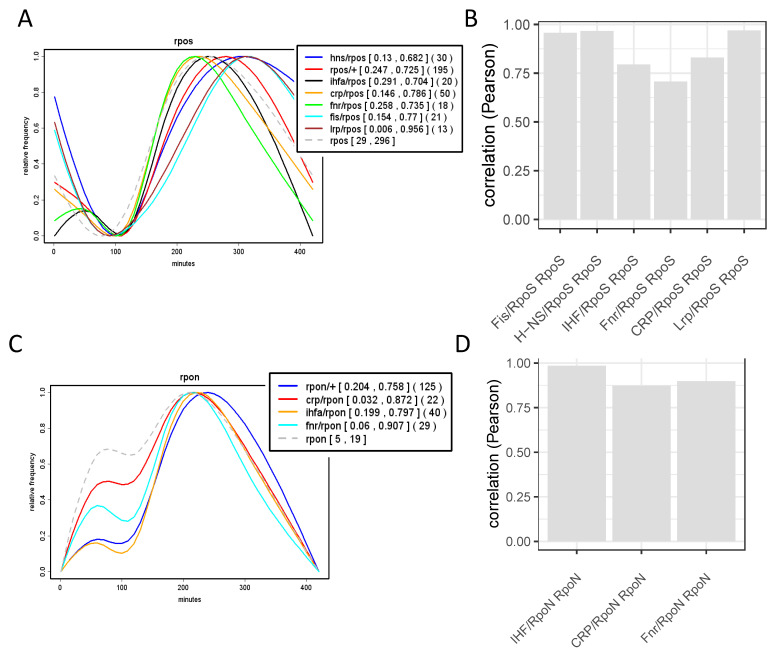
Correlation between the temporal expression of ASF regulons and couplons. (**A**). Growth phase-dependent expression of the RpoS regulon and RpoS/NAP couplons. The colored curves were computed as described in the legend to Figure 6. Abscissa—time in minutes after inoculation of cells in fresh growth medium; ordinate—relative frequency of each class of genes as indicated in the inset. (**B**). Pearson correlations (ordinate) between the temporal expression of RpoS regulon, RpoS/NAP and RpoS/global TF (CRP and Fnr) couplons (as indicated on the abscissa). (**C**,**D**). The same as in (**A**,**B**), but for the RpoN sigma factor. Note the high positive correlation between the temporal expression of both ASF (RpoS and RpoN) regulons and that of NAP/ASF and global TF/ASF couplons. The expression patterns of *rpoS* and *rpoN* genes (in **A** and **C**, respectively) are indicated by grey dashed lines. rpoS/+ and rpoN/+ respectively indicate the RpoS and RpoN regulons. Numbers in squared brackets indicate the minimum and maximum of the regulon/couplon before normalization to [0;1]. Note, that these numbers are averages of the regulon couplon genes already normalised individually to [0;1]. Numbers in round brackets indicate the number of genes involved in the respective regulon or couplon The experimental procedure was as described in the legend to Figure 6 (plots **A** and **C**, data from [29]).

**Figure 8:**
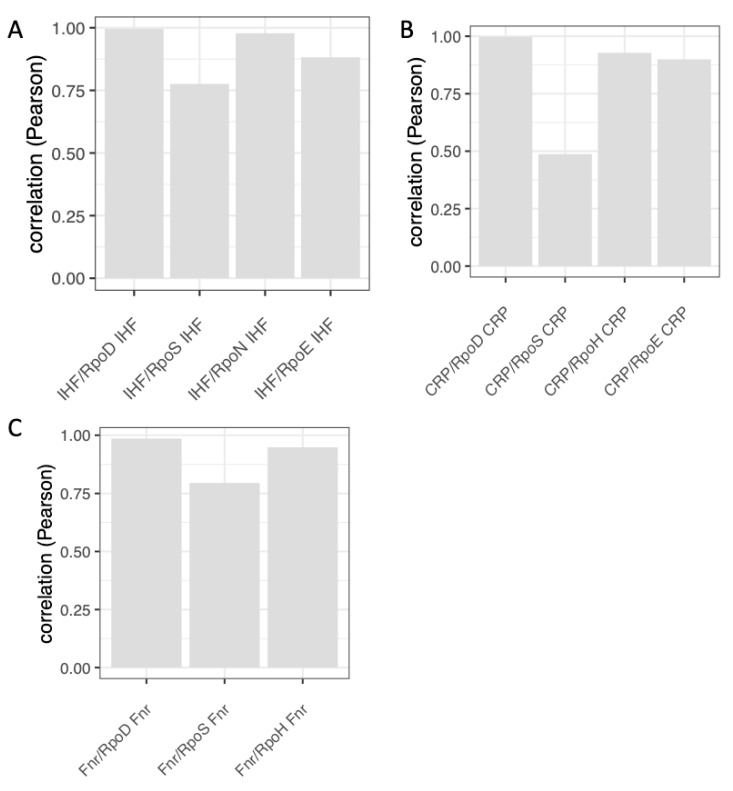
The temporal regulon/couplon correlation patterns for IHF and global TFs. (**A**). Pearson correlation between the expression of IHF regulon, IHF/RpoD and IHF/ASF couplons. (**B**,**C**), the same as in (**A**), but for CRP and Fnr, respectively Note that the couplon patterns show positive correlation with those of IHF and global TFs irrespective of the involved sigma factor (data from [29]).

Analyses of the growth phase-dependent expression patterns of regulons and couplons (computed as average patterns of all the genes in the given regulon/couplon and normalized to 1.0; for details see legend to Supplementary Figure S6) show that the temporal patterns of the regulons of NAPs (FIS, H-NS and Lrp) and the corresponding NAP/RpoD couplons are highly correlated (Figure 6). Thus, not only the spatial organization, but also the temporal expression patterns of the NAP/RpoD couplons are positively correlated with those of NAP regulons. In contrast, the growth phase-dependent expression patterns of the NAP/ASF couplons correlate with those of the ASF (RpoS and RpoN) regulons (Figure 7). Thus again, both the spatial organization and the temporal expression patterns of the NAP/ASF couplons are positively correlated with those of ASF regulons. However, the temporal patterns of the IHF/ASF, CRP/ASF and Fnr/ASF couplons demonstrate positive correlations with both of the parent regulons (compare Figure 7 and Figure 8), suggesting a joint impact of the latter on cognate couplon patterns.

**Figure 9:**
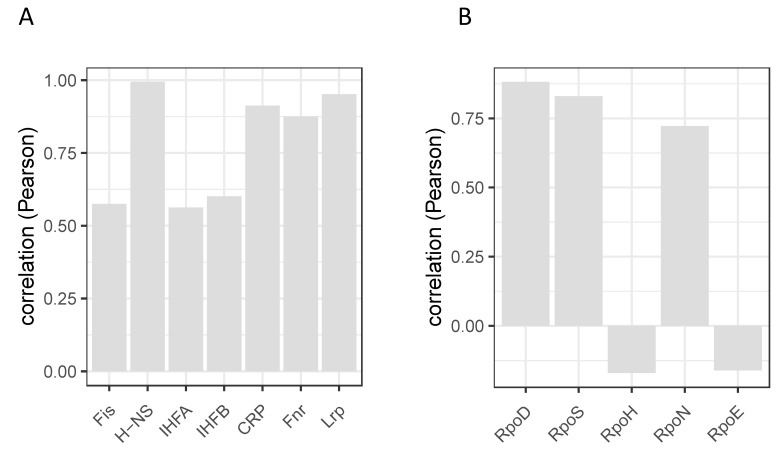
Correlation between the expression patterns of regulators and their cognate regulons. (**A**). Pearson correlation (ordinate) between the expression of NAP genes and their corresponding regulons. Individual regulators for which the correlations between cognate gene/regulon expression were calculated are indicated on the abscissa. (**B**). Pearson correlation between the expression of sigma factor genes and their corresponding regulons (data from [29]).

Thus it appears that the regulons have variable impacts on the temporal patterns of couplons. However, in that case, what determines the temporal patterns of regulons in the first place? Are the expression patterns of regulons determined by cognate regulators? Indeed, analyses showed that with notable exception of the *rpoH* and *rpoE* genes, in all cases a positive correlation obtains between the expression pattern of the regulatory gene and its cognate regulon (Figure 9). Since the expression of NAP genes is positively correlated with that of the NAP regulons, while the latter in turn appear determinative for the expression patterns of NAP/RpoD (but not the NAP/ASF) couplons (see Figure 6), this suggests that the temporal patterns of the NAP/RpoD couplons are, by and large, determined by expression patterns of the NAP genes. Conversely, the expression pattern of *rpoS* gene positively correlates with that of the RpoS regulon, whereas this latter appears determinative for the patterns of corresponding NAP/RpoS couplons (Figure 7B), suggesting that temporal expression patterns of these couplons are ultimately determined by the expression pattern of *rpoS* gene. Similar argument applies to *rpoN* gene, RpoN regulon and RpoN couplons. By the same token, whenever the temporal patterns of couplons correlate positively with those of both parent regulons, as is the case with the IHF and global TF couplons (compare Figure 7 and Figure 8), the assumption would be that the expression patterns of both regulatory genes jointly contribute to that of the couplons.

**Figure 10:**
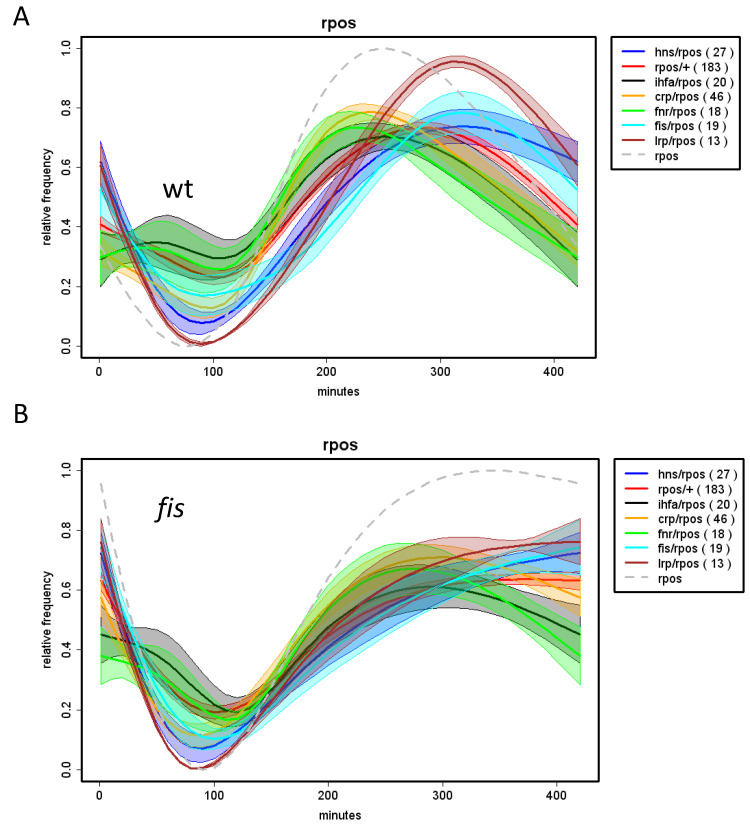
Modulation of the expression of ASF gene affects the temporal patterns of corresponding regulons and couplons. (**A**). Growth phase-dependent expression patterns of *rpoS* gene (grey dashed line), the RpoS regulon and Rpos/NAP couplons (color-coding indicated in the inset) in *E. coli* wild type (wt) cells. Numbers in round brackets indicate the number of genes involved in the respective regulon or couplon. Abscissa—time in minutes after inoculation of cells in fresh growth medium. The different curves were normalized to [0;1] to compare them in one plot. The envelopes of the curves indicate the standard deviation at 10% random remapping of the expression patterns to genes. (**B**). The same as in (**A**) but in the *fis* mutant cells. Note that mutation of *fis* gene alters the expression pattern of both, the *rpoS* gene and RpoS regulon, as well as RpoS couplons (data from [29]). The experimental procedure was as described in the legend to Figure 6.

**Figure 11:**
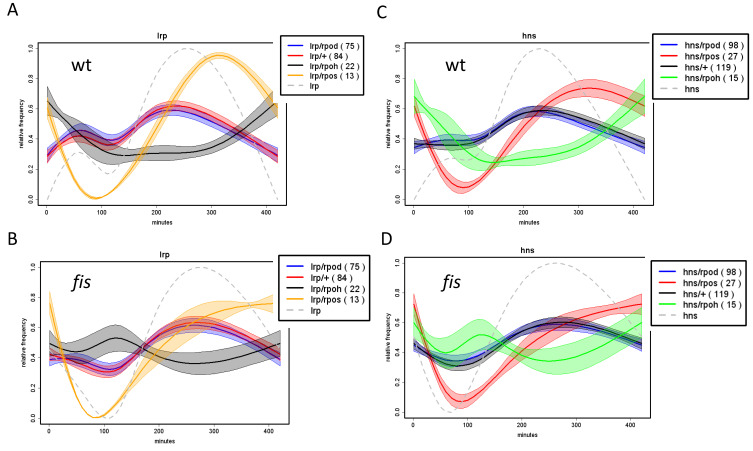
Modulation of the expression of NAP genes affects the temporal patterns of corresponding regulons and couplons. (**A**). Expression patterns of *lrp* gene Lrp regulon and Lrp couplons in wild type (wt) cells (color-code indicated in the inset). Numbers in round brackets indicate the number of genes involved in the respective regulon or couplon. The different curves were normalized to [0;1] to compare them in one plot. The envelopes of the curves indicate the standard deviation at 10% random remapping of the expression patterns to genes. (**B**). The same as in (**A**) but in the *fis* mutant cells. (**C**). Expression patterns of *hns* gene, H-NS regulon and H-NS couplons in wild type cells. (**D**). The same as in (**C**) but in the *fis* mutant cells. Note that mutation of *fis* gene alters the expression pattern of *lrp* and *hns* genes (grey dashed lines) as well as the patterns of Lrp and H-NS couplons (data from [29]). The experimental procedure was as described in the legend to Figure 6.

The dependence of temporal regulon/couplon patterns on the regulating gene expression is corroborated by observed impacts of altered regulator gene expression on the expression of cognate couplons and/or regulons. For example, FIS is a repressor of *rpoS* gene expression [114] and while inactivation of the *fis* gene by mutation abolishes the down-regulation of the *rpoS* gene expression in stationary phase (Figure 10; compare the grey dashed lines in A and B), the RpoS regulon and couplons react accordingly. Similarly, the *fis* mutation affects the expression of *lrp* gene and Lrp couplons and that of *hns* gene and H-NS couplons in a concerted manner (Figure 11). In all these cases altered regulator gene expression concomitantly alters the expression pattern of cognate couplons and/or regulon.

## 10. NAPs versus Global TFs?

Whereas expression of the NAP genes positively correlates with, and most likely defines, the temporal patterns of NAP/RpoD but not that of NAP/RpoS couplons, the expression patterns of the latter appear defined primarily by that of *rpoS* gene. Among the studied NAPs, only IHF does not conform to this rule—there is no such sigma factor-dependent difference of IHF impact (compare Figure 7B and Figure 8A) and in that sense, IHF shows similarity to the global TFs, CRP and Fnr.

This difference between IHF and the other NAPs is interesting and merits some elaboration. It is noteworthy that except IHF, all the NAPs examined here affect the global DNA topology by constraining DNA supercoils and stabilizing higher-order nucleoprotein structures, while IHF is apparently incapable of oligomerization. On binding DNA, IHF stabilizes planar bends rather than writhed coils [115] and affects local DNA trajectory, rather than global DNA topology [112,116]. The sequence-specificity of IHF binding is almost entirely determined by structural features of the DNA and not by direct readout of the base sequence [117], yet similar to the global TFs, IHF recognizes a relatively well-defined sequence motif [118] introducing sharp bends (U-turns) in the DNA. Stabilization of such tightly bent structures could explain, why IHF in contrast to other NAPs, is primarily affecting the directionality (leading/lagging strand bias) of genomic transcription with associated transcription-coupled supercoil diffusion [112]. Put another way, IHF exerts its primary effect at that level of genomic organization (mutual orientation of transcription units), for which the promoter sigma factor specificity is, most probably, immaterial.

The global TFs, CRP and Fnr, are structurally closely related sequence-specific DNA binding proteins [119] not known to constrain supercoils or form long-range nucleoprotein structures characteristic of NAPs, although DNA bending by CRP may assist in wrapping of DNA by RNAP—a characteristic associated with σ^70^ holoenzyme [66,120,121,122]. The debated difference between the NAPs and other global TFs [123] may lie by and large, in the prominent capacity of the former to constrain supercoils and stabilize long-range nucleoprotein structures, that is to say, in prevalence of analog over the digital mode of transcriptional control [5]. Notably, specific CRP binding activity strictly depends on cAMP levels [124], whereas Fnr utilizes iron-sulfur clusters as cofactors and is activated strictly under conditions of anaerobiosis [125]. It is conceivable that this strict dependence of specific binding effects of CRP and Fnr on physiological conditions would require tight coupling with ASFs in coordinating functional responses to physiological stress.

Interestingly, among the analyzed regulators, the *rpoH* and *rpoE* gene expression show no correlation with the expression patterns of corresponding regulons (Figure 9). In the case of extracytoplasmic sigma factor RpoE, this uncoupling of RpoE regulon pattern from *rpoE* gene expression might be related to the proteolytic cascade involving several factors and preceding the release of RpoE in the cytoplasm, whereas *rpoH* expression is in turn, under the control of RpoE [126,127]. Nevertheless, in the case of RpoS regulon we see a positive correlation with *rpoS* gene expression, despite the complex regulation of cellular RpoS levels [128] and coupling of RpoS effects to changes of DNA topology [129].

## 11. Conclusions

Overall, we observe positive correlation between the temporal expression of global regulatory genes and their cognate regulons (see Figure 9) as well as between the regulons and couplons (see Figure 6, Figure 7 and Figure 8) on the one hand, and positive correlations between the spatial organization of regulons and couplons (in terms of their genomic distribution patterns; see Figure 5) on the other. Taken together, these observations suggest that the expression of global regulators is coordinated with organization of target genes in the chromosome, lending support to the hypothesis that transcriptional regulation shapes the spatial organization of genes [104,130]. The determinative impact of the regulons of NAPs and global TFs on spatial patterns of cognate RpoD couplons is apparently related to the fact that RpoD transcribes vast majority of the genes and so, tends to impose uniformity, whereas the effects of NAPs and global TFs regulating subsets of genes (as defined by the TRN) convey discreteness to the spatial couplon patterns (Figure 5A,C; Supplementary Figures S3 and S4). The alternative sigma factors are associated with particular functions [85] and also in this case, we see that the spatial impacts of the ASF regulons predominate (Figure 5B,D; Supplementary Figure S5). However, this does not explain why the regulon and couplon genes are organized in spatially defined clusters (see Figure 5 and Supplementary Figures S1–S5).

While the relation of regulatory gene expression pattern to the temporal regulon (or couplon) patterns appears trivial, the question is how the expression pattern of a regulator (be it a NAP or sigma factor) is related to spatial organization of cognate regulons and couplons in genome. One relevant observation is the distinct organization of the NAP and TF genes with respect to their targets in genome, indicating that the main parameters defining the position of a TF in the network hierarchy are the number and chromosomal distances of the genes they regulate and their protein concentration gradients [130]. Another relevant observation is that regulatory genes form gradients diffusing from their sites of production [131,132]. It is thus conceivable that the observed spatial patterns of regulons and couplons are selected to optimize the exposure of target genes to the gradients of diffusing regulators. Furthermore, recent observations that alterations of gene expression and phenotype can be induced by chromosomal position shift of a NAP gene, despite the maintenance of its natural expression pattern [105] and, that the effect of positional shift can be aggravated by altering the expression pattern of the NAP gene [107], support the proposal that the physical structure of chromosome is optimized by direct regulatory interactions involving the NAPs and other DNA structuring proteins [133]. In this respect it is relevant, that the NAPs can distinctly modulate the chromosomal regional dynamics by inducing transient domain boundaries in the genome [134,135]. Such adjustment would involve selection for efficient interactions between the growth phase-dependent concentration gradients of regulators and chromosomal domains by restricting the range of chromosome configurations within the available configuration space, delimited by various factors including macromolecular crowding, entropic repulsion, confinement-induced organisation and packing density of the DNA polymer. The spatiotemporal coupling between the *E. coli* chromosomal gene order and expression, the spatial organization of transcriptional regulation on both chromosomal arms along the OriC-Ter axis [103,104] the successive, growth phase-dependent activation of the OriC and Ter chromosomal ends [30,103] and clustering of co-functional genes [136] are all consistent with this notion. Notably, dynamic 3D colocalization of co-regulated genes optimized by locally increasing concentration of transcription factors has been proposed to occur in yeast [137]. It is obvious, that the evolutionary process of adjusting chromosome configuration dynamics and gene organization would be impacted by chromosomal replication [138]. Understanding the corresponding growth phase-dependent compositional changes of the RNAP supramolecular complex and underpinning cellular hyperstructures [139] both of which could be dependent on energy and resource availability, require further studies.

## Figures and Tables

**Table 1:** Relevant genes up-regulated under conditions of norfloxacin-induced hypernegative DNA supercoiling and DNA relaxation in exponentially growing *E.coli* LZ54 and LZ41 wild-type (WT) strains and their mutant derivatives lacking the NAP-encoding genes *fis*, *hns* or both (data from [15] and unpublished results).

Condition	Hypernegative Supercoiling				DNA relaxation			
Strain, Background	LZ54WT	∆*fis*	∆*hns*	∆*fis/hns*	LZ41WT	∆*fis*	∆*hns*	∆*fis/hns*
Genes of NAPs and global TFs	*fis*	*lrp*		*lrp* *hupB* *ihfA* *stpA*	*hupA*	*fnr* *ihfB*	*fnr*	*fnr* *crp*
Genes of RNAP modulators	*crl* *nusG* *nusB*	*crl* *rho*	*crl* *nusG*	*nusG* *rho*			*greA*	*greA*
Genes of the RNAP sigma and anti-sigma factors		*rpoE* *rseAB*	*rpoE*	*rseA*	*rpoH*	*rpoD*	*rpoH* *rpoD* *fecI*	*rpoH* *rpoD* *fliA*
Topoisomerase genes	*yrdD* *parC*	*yrdD* *parC*	*yrdD* *parC*	*yrdD* *parE*	*gyrA* *gyrB*	*gyrB*	*gyrB*	*gyrA* *gyrB*

**Table 2:** Impacts of NAPs/global TFs and the RNAP sigma factors in the transcript profiles of growing cells (data from [39]). The first two columns compare the relative impacts in the profiles of wild-type and *rpoZ* mutants, the next two columns compare those in *rpoZ* mutant with and without *rpoD* overexpression.

Strain, Condition	CF1943 Wild Type	CF2790 *rpoZ*	CF2790 rpoZ + rpoD *	CF2790 *rpoZ*
Impacts of NAPs & global TFs	FIS, IHF	CRP	IHF, CRP, Fnr	H-NS, FIS, Lrp
Impacts of RNAP sigma subunits	RpoD	RpoS	RpoD	RpoS
Regulons up		RpoS	CRP, Fnr	RpoS
Couplons up		H-NS/RpoS	CRP/RpoD Fnr/RpoD	CRP/RpoS FIS/RpoS Lrp/RpoS IHF/RpoS Fnr/RpoS

* CF2790 *rpoZ* mutant strain grown with episomal *rpoD* overexpression.

## Supplementary Materials

The following are available online at https://www.mdpi.com/article/10.3390/biom11070924/s1: List of abbreviations used in the text, Figure S1: Genomic organization of the sigma factor regulons; Figure S2: Genomic organization of the NAP regulons; Figure S3: Comparison of the genomic organization of NAP regulons and NAP/RpoD couplons; Figure S4: Comparison of the genomic organization of CRP and Fnr regulons and their cognate RpoD couplons; Figure S5: Comparison of the genomic organization of the NAP and the ASF regulons and cognate couplons; Figure S6: Determination of the temporal expression of regulons and couplons on the example of FIS regulon and, the FIS/RpoD and FIS/RpoS couplons; Supplementary Table S1: Identities of proteins discovered in the RNAP supramolecular complex and their functions; Experimental procedure for Figure 2; Master thesis by Steffi Jimmy: “A study of RNA polymerase and nucleoid-associated protein composition in *Escherichia coli* stringent mutants”.

## References

[1] Travers A., Muskhelishvili G. (2005). DNA supercoiling—A global transcriptional regulator for enterobacterial growth?. Nat. Rev. Microbiol..

[2] Johansson J., Balsalobre C., Wang S.-Y., Urbonaviciene J., Jin D.J., Sondén B., Uhlin B.E., Johansson J., Balsalobre C., Wang S.-Y. (2000). Nucleoid proteins stimulate stringently controlled bacterial promoters: A link between the cAMP-CRP and the (p)ppGpp regulons in *Escherichia coli*. Cell.

[3] Muskhelishvili G., Sobetzko P., Geertz M., Berger M. (2010). General organisational principles of the transcriptional regulation system: A tree or a circle?. Mol. BioSyst..

[4] Cho B.-K., Kim D., Knight E.M., Zengler K., Palsson B.O. (2014). Genome-scale reconstruction of the sigma factor network in *Escherichia coli*: Topology and functional states. BMC Biol..

[5] Marr C., Geertz M., Hütt M.-T., Muskhelishvili G. (2008). Dissecting the logical types of network control in gene expression profiles. BMC Syst. Biol..

[6] Muskhelishvili G., Travers A. (2013). Integration of syntactic and semantic properties of the DNA code reveals chromosomes as thermodynamic machines converting energy into information. Cell. Mol. Life Sci..

[7] Beber M.E., Sobetzko P., Muskhelishvili G., Hütt M.-T. (2016). Interplay of digital and analog control in time-resolved gene expression profiles. EPJ Nonlinear Biomed. Phys..

[8] Salgado H., Peralta-Gil M., Gama-Castro S., Santos-Zavaleta A., Muñiz-Rascado L., García-Sotelo J.S., Weiss V., Solano-Lira H., Martínez-Flores I., Medina-Rivera A. (2012). RegulonDB v8.0: Omics data sets, evolutionary conservation, regulatory phrases, cross-validated gold standards and more. Nucleic Acids Res..

[9] Sonnenschein N., Hütt M.-T., Stoyan H., Stoyan D. (2009). Ranges of control in the transcriptional regulation of *Escherichia coli*. BMC Syst. Biol..

[10] Brinza L., Calevro F., Charles H. (2013). Genomic analysis of the regulatory elements and links with intrinsic DNA structural properties in the shrunken genome of Buchnera. BMC Genom..

[11] Junier I., Frémont P., Rivoire O. (2018). Universal and idiosyncratic characteristic lengths in bacterial genomes. Phys. Biol..

[12] El Houdaigui B., Forquet R., Hindré T., Schneider D., Nasser W., Reverchon S., Meyer S. (2019). Bacterial genome architecture shapes global transcriptional regulation by DNA supercoiling. Nucleic Acids Res..

[13] Peter B.J., Arsuaga J., Breier A.M., Khodursky A.B., Brown P.O., Cozzarelli N.R. (2004). Genomic transcriptional response to loss of chromosomal supercoiling in *Escherichia coli*. Genome Biol..

[14] Jeong K.S., Ahn J., Khodursky A.B. (2004). Spatial patterns of transcriptional activity in the chromosome of *Escherichia coli*. Genome Biol..

[15] Blot N., Mavathur R., Geertz M., Travers A., Muskhelishvili G. (2006). Homeostatic regulation of supercoiling sensitivity coordinates transcription of the bacterial genome. EMBO Rep..

[16] Jiang X., Sobetzko P., Nasser W., Reverchon S., Muskhelishvili G. (2015). Chromosomal “Stress-Response” Domains Govern the Spatiotemporal Expression of the Bacterial Virulence Program. mBio.

[17] Martis B.S., Forquet R., Reverchon S., Nasser W., Meyer S. (2019). DNA Supercoiling: An Ancestral Regulator of Gene Expression in Pathogenic Bacteria?. Comput. Struct. Biotechnol. J..

[18] Hsieh L.S., Rouviere-Yaniv J., Drlica K. (1991). Bacterial DNA supercoiling and [ATP]/[ADP] ratio: Changes associated with salt shock. J. Bacteriol..

[19] McClellan J.A., Boublikova P., Palecek E., Lilley D.M. (1990). Superhelical torsion in cellular DNA responds directly to environmental and genetic factors. Proc. Natl. Acad. Sci. USA.

[20] Workum M., Dooren S.J.M., Oldenburg N., Molenaar D., Jensen P.R., Snoep J.L., Westerhoff H. (1996). DNA supercoiling depends on the phosphorylation potential in *Escherichia coli*. Mol. Microbiol..

[21] Sonnenschein N., Geertz M., Muskhelishvili G., Hütt M.-T. (2011). Analog regulation of metabolic demand. BMC Syst. Biol..

[22] Berthoumieux S., De Jong H., Baptist G., Pinel C., Ranquet C., Ropers D., Geiselmann J. (2013). Shared control of gene expression in bacteria by transcription factors and global physiology of the cell. Mol. Syst. Biol..

[23] Dillon S.C., Dorman C. (2010). Bacterial nucleoid-associated proteins, nucleoid structure and gene expression. Nat. Rev. Microbiol..

[24] Luijsterburg M.S., White M., Van Driel R., Dame R.T. (2008). The Major Architects of Chromatin: Architectural Proteins in Bacteria, Archaea and Eukaryotes. Crit. Rev. Biochem. Mol. Biol..

[25] Rimsky S., Travers A. (2011). Pervasive regulation of nucleoid structure and function by nucleoid-associated proteins. Curr. Opin. Microbiol..

[26] Schultz S.G., Epstein W., Solomon A.K. (1963). Cation Transport in *Escherichia coli*. J. Gen. Physiol..

[27] Dinnbier U., Limpinsel E., Schmid R., Bakker E.P. (1988). Transient accumulation of potassium glutamate and its replacement by trehalose during adaptation of growing cells of *Escherichia coli* K-12 to elevated sodium chloride concentrations. Arch. Microbiol..

[28] McLaggan D., Naprstek J., Buurman E., Epstein W. (1994). Interdependence of K+ and glutamate accumulation during osmotic adaptation of *Escherichia coli*. J. Biol. Chem..

[29] Sobetzko P., Glinkowska M., Muskhelishvili G. (2017). GSE65244: Temporal Gene Expression in *Escherichia coli*. Gene Expression Omnibus. https://www.ncbi.nlm.nih.gov/geo/query/acc.cgi?acc=GSE65244.

[30] Sobetzko P., Glinkowska M., Travers A., Muskhelishvili G. (2013). DNA thermodynamic stability and supercoil dynamics determine the gene expression program during the bacterial growth cycle. Mol. BioSyst..

[31] Stock C., Hielkema L., Tascón I., Wunnicke D., Oostergetel G.T., Azkargorta M., Paulino C., Hänelt I. (2018). Cryo-EM structures of KdpFABC suggest a K+ transport mechanism via two inter-subunit half-channels. Nat. Commun..

[32] Cheng X., Guinn E.J., Buechel E., Wong R., Sengupta R., Shkel I.A., Record M.T. (2016). Basis of Protein Stabilization by K Glutamate: Unfavorable Interactions with Carbon, Oxygen Groups. Biophys. J..

[33] Blundell T., Barlow D., Borkakoti N., Thornton J. (1983). Solvent-induced distortions and the curvature of α-helices. Nature.

[34] Anderson P., Bauer W. (1978). Supercoiling in closed circular DNA: Dependence upon ion type and concentration. Biochemistry.

[35] Rybenkov V.V., Vologodskii A.V., Cozzarelli N.R. (1997). The effect of ionic conditions on DNA helical repeat, effective diameter and free energy of supercoiling. Nucleic Acids Res..

[36] Wu C., Travers A. (2019). Modelling and DNA topology of compact 2-start and 1-start chromatin fibres. Nucleic Acids Res..

[37] Ohlsen K.L., Gralla J.D. (1992). Melting during steady-state transcription of the rrnB P1 promoter in vivo and in vitro. J. Bacteriol..

[38] Travers A., Muskhelishvili G. (2020). Chromosomal Organization and Regulation of Genetic Function in *Escherichia coli* Integrates the DNA Analog and Digital Information. EcoSal Plus.

[39] Geertz M., Travers A., Mehandziska S., Sobetzko P., Janga S.C., Shimamoto N., Muskhelishvili G. (2011). Structural coupling between RNA polymerase composition and DNA supercoiling in coordinating transcription: A global role for the omega subunit?. mBio.

[40] Vinella D., Potrykus K., Murphy H., Cashel M. (2012). Effects on growth by changes of the balance between GreA, GreB, and DksA suggest mutual competition and functional redundancy in *Escherichia coli*. J. Bacteriol..

[41] Cheng B., Zhu C.-X., Ji C., Ahumada A., Tse-Dinh Y.-C. (2003). Direct interaction between *Escherichia coli* RNA polymerase and the zinc ribbon domains of DNA topoisomerase I. J. Biol. Chem..

[42] Gupta R., China A., Manjunatha U.H., Ponnanna N., Nagaraja V. (2006). A complex of DNA gyrase and RNA polymerase fosters transcription in *Mycobacterium smegmatis*. Biochem. Biophys. Res. Commun..

[43] Verma S., Xiong Y., Mayer M.U., Squier T.C. (2007). Remodeling of the bacterial RNA polymerase supramolecular complex in response to environmental conditions. Biochemistry.

[44] Banda S., Cao N., Tse-Dinh Y.-C. (2017). Distinct Mechanism Evolved for Mycobacterial RNA Polymerase and Topoisomerase I Protein-Protein Interaction. J. Mol. Biol..

[45] Mehandziska S., Petrescu A.M., Muskhelishvili G. (2017). Isolation and Analysis of RNA Polymerase Supramolecular Complex with Associated Proteins. Methods Mol. Biol..

[46] Eason I.R., Kaur H.P., Alexander K.A., Sukhodolets M.V. (2019). Growth phase-specific changes in the composition of *E. coli* transcription complexes. J. Chromatogr. B.

[47] Kohler R., Mooney R.A., Mills D.J., Landick R., Cramer P. (2017). Architecture of a transcribing-translating expressome. Science.

[48] Johnson G.E., Lalanne J.-B., Peters M.L., Li G.-W. (2020). Functionally uncoupled transcription–translation in *Bacillus subtilis*. Nature.

[49] Singh N., Bubunenko M., Smith C., Abbott D.M., Stringer A.M., Shi R., Court D.L., Wade J.T. (2016). SuhB Associates with Nus Factors to Facilitate 30S Ribosome Biogenesis in *Escherichia coli*. mBio.

[50] Dudenhoeffer B.R., Schneider H., Schweimer K., Knauer S.H. (2019). SuhB is an integral part of the ribosomal antitermination complex and interacts with NusA. Nucleic Acids Res..

[51] Hedstrom L. (2009). IMP Dehydrogenase: Structure, mechanism, and inhibition. Chem. Rev..

[52] McLean J.E., Hamaguchi N., Belenky P., Mortimer S.E., Stanton M., Hedstrom L. (2004). Inosine 5′-monophosphate dehydrogenase binds nucleic acids in vitro and in vivo. Biochem. J..

[53] Kozhevnikova E.N., van der Knaap J.A., Pindyurin A.V., Ozgur Z., van Ijcken W., Moshkin Y.M., Verrijzer C.P. (2012). Metabolic enzyme IMPDH is also a transcription factor regulated by cellular state. Mol. Cell.

[54] Pimkin M., Pimkina J., Markham G.D. (2009). A regulatory role of the Bateman domain of IMP dehydrogenase in adenylate nucleotide biosynthesis. J. Biol. Chem..

[55] Snoep J.L., van der Weijden C.C., Andersen H.W., Westerhoff H.V., Jensen P.R. (2002). DNA supercoiling in *Escherichia coli* is under tight and subtle homeostatic control, involving gene-expression and metabolic regulation of both topoisomerase I and DNA gyrase. Eur. J. Biochem..

[56] Riles L., Shaw R.J., Johnston M., Reines D. (2004). Large-scale screening of yeast mutants for sensitivity to the IMP dehydrogenase inhibitor 6-azauracil. Yeast.

[57] Jurkiewicz A., Leśniewska E., Cieśla M., Gorjão N., Kantidakis T., White R.J., Boguta M., Graczyk D. (2019). Inhibition of tRNA Gene Transcription by the Immunosuppressant Mycophenolic Acid. Mol. Cell. Biol..

[58] Mortimer S.E., Xu D., McGrew D., Hamaguchi N., Lim H.C., Bowne S.J., Daiger S.P., Hedstrom L. (2008). IMP dehydrogenase type 1 associates with polyribosomes translating rhodopsin mRNA. J. Biol. Chem..

[59] Böhringer J., Fischer D., Mosler G., Hengge-Aronis R. (1995). UDP-glucose is a potential intracellular signal molecule in the control of expression of sigma S and sigma S-dependent genes in *Escherichia coli*. J. Bacteriol..

[60] Krause K., Maciąg-Dorszyńska M., Wosinski A., Gaffke L., Morcinek-Orłowska J., Rintz E., Bielańska P., Szalewska-Pałasz A., Muskhelishvili G., Węgrzyn G. (2020). The Role of Metabolites in the Link between DNA Replication and Central Carbon Metabolism in *Escherichia coli*. Genes.

[61] Berger M., Farcas A., Geertz M., Zhelyazkova P., Brix K., Travers A., Muskhelishvili G. (2009). Coordination of genomic structure and transcription by the main bacterial nucleoid-associated protein HU. EMBO Rep..

[62] Weng X., Bohrer C.H., Bettridge K., Lagda A.C., Cagliero C., Jin D.J., Xiao J. (2019). Spatial organization of RNA polymerase and its relationship with transcription in *Escherichia coli*. Proc. Natl. Acad. Sci. USA.

[63] Liu L.F., Wang J.C. (1987). Supercoiling of the DNA template during transcription. Proc. Natl. Acad. Sci. USA.

[64] Sutormin D., Rubanova N., Logacheva M., Ghilarov D., Severinov K. (2018). Single-nucleotide-resolution mapping of DNA gyrase cleavage sites across the *Escherichia coli* genome. Nucleic Acids Res..

[65] Muskhelishvili G., Travers A. (2003). Transcription factor as a topological homeostat. Front. Biosci..

[66] Maurer S., Fritz J., Muskhelishvili G., Travers A. (2006). RNA polymerase and an activator form discrete subcomplexes in a transcription initiation complex. EMBO J..

[67] Muskhelishvili G., Travers A. (2016). The regulatory role of DNA supercoiling in nucleoprotein complex assembly and genetic activity. Biophys. Rev..

[68] Menzel R., Gellert M. (1983). Regulation of the genes for *E. coli* DNA gyrase: Homeostatic control of DNA supercoiling. Cell.

[69] Gaal T., Mandel M.J., Silhavy T.J., Gourse R.L., Gaal T., Mandel M.J., Silhavy T.J., Gourse R.L. (2006). Crl facilitates RNA polymerase holoenzyme formation. J. Bacteriol..

[70] Typas A., Barembruch C., Possling A., Hengge R. (2007). Stationary phase reorganisation of the *Escherichia coli* transcription machinery by Crl protein, a fine-tuner of σs activity and levels. EMBO J..

[71] Banta A., Chumanov R.S., Yuan A.H., Lin H., Campbell E., Burgess R.R., Gourse R.L. (2013). Key features of S required for specific recognition by Crl, a transcription factor promoting assembly of RNA polymerase holoenzyme. Proc. Natl. Acad. Sci. USA.

[72] Minakhin L., Bhagat S., Brunning A., Campbell E., Darst S.A., Ebright R., Severinov K. (2001). Bacterial RNA polymerase subunit omega and eukaryotic RNA polymerase subunit RPB6 are sequence, structural, and functional homologs and promote RNA polymerase assembly. Proc. Natl. Acad. Sci. USA.

[73] Artsimovitch I., Landick R. (2000). Pausing by bacterial RNA polymerase is mediated by mechanistically distinct classes of signals. Proc. Natl. Acad. Sci. USA.

[74] Burmann B.M., Schweimer K., Luo X., Wahl M., Stitt B.L., Gottesman M.E., Rösch P., Burmann B.M., Schweimer K., Luo X. (2010). A NusE:NusG complex links transcription and translation. Science.

[75] Webster M.W., Takacs M., Zhu C., Vidmar V., Eduljee A., Abdelkareem M., Weixlbaumer A. (2020). Structural basis of transcription-translation coupling and collision in bacteria. Science.

[76] Wang C., Molodtsov V., Firlar E., Kaelber J.T., Blaha G., Su M., Ebright R.H. (2020). Structural basis of transcription-translation coupling. Science.

[77] Washburn R.S., Zuber P.K., Sun M., Hashem Y., Shen B., Li W., Harvey S., Reyes F.J.A., Gottesman M.E., Knauer S.H. (2020). *Escherichia coli* NusG Links the Lead Ribosome with the Transcription Elongation Complex. iScience.

[78] Dutta D., Shatalin K., Epshtein V., Gottesman M.E., Nudler E. (2011). Linking RNA polymerase backtracking to genome instability in *E. coli*. Cell.

[79] Cardinale C.J., Washburn R.S., Tadigotla V.R., Brown L.M., Gottesman M.E., Nudler E. (2008). Termination factor Rho and its cofactors NusA and NusG silence foreign DNA in *E. coli*. Science.

[80] Peters J.M., Mooney R.A., Kuan P.F., Rowland J.L., Keleş S., Landick R. (2009). Rho directs widespread termination of intragenic and stable RNA transcription. Proc. Natl. Acad. Sci. USA.

[81] Said N., Hilal T., Sunday N.D., Khatri A., Bürger J., Mielke T., Belogurov G.A., Loll B., Sen R., Artsimovitch I. (2020). Steps toward translocation-independent RNA polymerase inactivation by terminator ATPase ρ. Science.

[82] Leela J.K., Syeda A.H., Anupama K., Gowrishankar J. (2012). Rho-dependent transcription termination is essential to prevent excessive genome-wide R-loops in *Escherichia coli*. Proc. Natl. Acad. Sci. USA.

[83] Raghunathan N., Kapshikar R.M., Leela J.K., Mallikarjun J., Bouloc P., Gowrishankar J. (2018). Genome-wide relationship between R-loop formation and antisense transcription in *Escherichia coli*. Nucleic Acids Res..

[84] Rowley G., Spector M., Kormanec J., Roberts M. (2006). Pushing the envelope: Extracytoplasmic stress responses in bacterial pathogens. Nat. Rev. Microbiol..

[85] Paget M.S. (2015). Bacterial Sigma Factors and Anti-Sigma Factors: Structure, Function and Distribution. Biomolecules.

[86] Fernández-Coll L., Maciag-Dorszynska M., Tailor K., Vadia S., Levin P.A., Szalewska-Palasz A., Cashel M. (2020). The Absence of (p)ppGpp Renders Initiation of *Escherichia coli* Chromosomal DNA Synthesis Independent of Growth Rates. mBio.

[87] Rochman M., Aviv M., Glaser G., Muskhelishvili G. (2002). Promoter protection by a transcription factor acting as a local topological homeostat. EMBO Rep..

[88] Potrykus K., Vinella D., Murphy H., Szalewska-Palasz A., D’Ari R., Cashel M. (2006). Antagonistic regulation of *Escherichia coli* ribosomal RNA rrnB P1 promoter activity by GreA and DksA. J. Biol. Chem..

[89] Ueshima R., Fujita N., Ishihama A. (1989). DNA supercoiling and temperature shift affect the promoter activity of the *Escherichia coli* rpoH gene encoding the heat-shock sigma subunit of RNA polymerase. Mol. Genet. Genom..

[90] Sudzinová P., Kambová M., Ramaniuk O., Benda M., Šanderová H., Krásný L. (2021). Effects of DNA Topology on Transcription from rRNA Promoters in *Bacillus subtilis*. Microorganisms.

[91] Kusano S., Ding Q., Fujita N., Ishihama A. (1996). Promoter selectivity of *Escherichia coli* RNA polymerase E sigma 70 and E sigma 38 holoenzymes. Effect of DNA supercoiling. J. Biol. Chem..

[92] Schneider R., Travers A., Muskhelishvili G. (2000). The expression of the Escherichia coli fis gene is strongly dependent on the superhelical density of DNA. Mol. Microbiol..

[93] González-Gil G., Kahmann R., Muskhelishvili G. (1998). Regulation of crp transcription by oscillation between distinct nucleoprotein complexes. EMBO J..

[94] Levanon S.S., San K.-Y., Bennett G.N. (2005). Effect of oxygen on the *Escherichia coli* ArcA and FNR regulation systems and metabolic responses. Biotechnol. Bioeng..

[95] Berger M., Gerganova V., Berger P., Rapiteanu R., Lisicovas V., Dobrindt U. (2016). Genes on a Wire: The Nucleoid-Associated Protein HU Insulates Transcription Units in *Escherichia coli*. Sci. Rep..

[96] Claret L., Rouviere-Yaniv J. (1997). Variation in HU composition during growth of *Escherichia coli*: The heterodimer is required for long term survival. J. Mol. Biol..

[97] Zhou Y.N., Jin D.J. (1998). The rpoB mutants destabilizing initiation complexes at stringently controlled promoters behave like “stringent” RNA polymerases in *Escherichia coli*. Proc. Natl. Acad. Sci. USA.

[98] Balke V.L., Gralla J.D. (1987). Changes in the linking number of supercoiled DNA accompany growth transitions in *Escherichia coli*. J. Bacteriol..

[99] Schmid M.B., Roth J.R. (1987). Gene location affects expression level in *Salmonella typhimurium*. J. Bacteriol..

[100] Sousa C., de Lorenzo V., Cebolla A. (1997). Modulation of gene expression through chromosomal positioning in *Escherichia coli*. Microbiology.

[101] Rocha E.P., Danchin A. (2003). Gene essentiality determines chromosome organisation in bacteria. Nucleic Acids Res..

[102] Fang G., Rocha E.P., Danchin A. (2008). Persistence drives gene clustering in bacterial genomes. BMC Genom..

[103] Sobetzko P., Travers A., Muskhelishvili G. (2011). Gene order and chromosome dynamics coordinate spatiotemporal gene expression during the bacterial growth cycle. Proc. Natl. Acad. Sci. USA.

[104] Kosmidis K., Jablonski K.P., Muskhelishvili G., Hütt M.-T. (2020). Chromosomal origin of replication coordinates logically distinct types of bacterial genetic regulation. NPJ Syst. Biol. Appl..

[105] Gerganova V., Berger M.F., Zaldastanishvili E., Sobetzko P., Lafon C., Mourez M., Travers A., Muskhelishvili G. (2015). Chromosomal position shift of a regulatory gene alters the bacterial phenotype. Nucleic Acids Res..

[106] Soler-Bistué A., Timmermans M., Mazel D. (2017). The Proximity of Ribosomal Protein Genes to *oriC* Enhances *Vibrio cholerae* Fitness in the Absence of Multifork Replication. mBio.

[107] Bogue M.M., Mogre A., Beckett M.C., Thomson N.R., Dorman C.J. (2020). Network Rewiring: Physiological Consequences of Reciprocally Exchanging the Physical Locations and Growth-Phase-Dependent Expression Patterns of the *Salmonella fis* and *dps* Genes. mBio.

[108] Nigatu D., Henkel W., Sobetzko P., Muskhelishvili G. (2016). Relationship between digital information and thermodynamic stability in bacterial genomes. EURASIP J. Bioinform. Syst. Biol..

[109] Travers A.A., Muskhelishvili G. (2013). DNA thermodynamics shape chromosome organization and topology. Biochem. Soc. Trans..

[110] Meyer S., Reverchon S., Nasser W., Muskhelishvili G. (2017). Chromosomal organization of transcription: In a nutshell. Curr. Genet..

[111] Muskhelishvili G., Forquet R., Reverchon S., Meyer S., Nasser W. (2019). Coherent Domains of Transcription Coordinate Gene Expression During Bacterial Growth and Adaptation. Microorganisms.

[112] Reverchon S., Meyer S., Forquet R., Hommais F., Muskhelishvili G., Nasser W. (2020). The nucleoid-associated protein IHF acts as a ‘transcriptional domainin’ protein coordinating the bacterial virulence traits with global transcription. Nucleic Acids Res..

[113] Shimada T., Tanaka K., Ishihama A. (2017). The whole set of the constitutive promoters recognized by four minor sigma subunits of *Escherichia coli* RNA polymerase. PLoS ONE.

[114] Hirsch M., Elliott T., Hirsch M., Elliott T. (2005). Fis regulates transcriptional induction of RpoS in *Salmonella enterica*. J. Bacteriol..

[115] Rice P.A. (1997). Making DNA do a U-turn: IHF and related proteins. Curr. Opin. Struct. Biol..

[116] Pagel J.M., Winkelman J.W., Adams C.W., Hatfield G. (1992). DNA topology-mediated regulation of transcription initiation from the tandem promoters of the ilvGMEDA operon of *Escherichia coli*. J. Mol. Biol..

[117] Travers A. (1997). DNA-protein interactions: IHF—The master bender. Curr. Biol..

[118] Ellenberger T., Landy A. (1997). A good turn for DNA: The structure of integration host factor bound to DNA. Structure.

[119] Green J., Scott C., Guest J.R. (2001). Functional versatility in the CRP-FNR superfamily of transcription factors: FNR and FLP. Adv. Microb. Physiol..

[120] Amouyal M., Buc H. (1987). Topological unwinding of strong and weak promoters by RNA polymerase: A comparison between the lac wild-type and the UV5 sites of *Escherichia coli*. J. Mol. Biol..

[121] Shin M., Song M., Rhee J.H., Hong Y., Kim Y.-J., Seok Y.-J., Ha K.-S., Jung S.-H., Choy H.E. (2005). DNA looping-mediated repression by histone-like protein H-NS: Specific requirement of Esigma70 as a cofactor for looping. Genes Dev..

[122] Cellai S., Mangiarotti L., Vannini N., Naryshkin N., Kortkhonjia E., Ebright R.H., Rivetti C. (2007). Upstream promoter sequences and alphaCTD mediate stable DNA wrapping within the RNA polymerase–promoter open complex. EMBO Rep..

[123] Dorman C.J., Schumacher M.A., Bush M., Brennan R.G., Buttner M.J. (2020). When is a transcription factor a NAP?. Curr. Opin. Microbiol..

[124] Harman J.G. (2001). Allosteric regulation of the cAMP receptor protein. Biochim. Biophys. Acta..

[125] Mettert E.L., Kiley P.J. (2018). Reassessing the Structure and Function Relationship of the O2 Sensing Transcription Factor FNR. Antioxid. Redox Signal..

[126] Alba B.M., Gross C.A. (2004). Regulation of the *Escherichia coli* sigma-dependent envelope stress response. Mol. Microbiol..

[127] Rhodius V.A., Suh W.C., Nonaka G., West J., Gross C.A. (2005). Conserved and variable Functions of the sigmaE stress response in related genomes. PLoS Biol..

[128] Hengge-Aronis R. (2002). Signal transduction and regulatory mechanisms involved in control of the sigma(S) (RpoS) subunit of RNA polymerase. Microbiol. Mol. Biol. Rev..

[129] Bordes P., Conter A., Morales V., Bouvier J., Kolb A., Gutierrez C. (2003). DNA supercoiling contributes to disconnect sigma^S^ accumulation from sigma^S^-dependent transcription in *Escherichia coli*. Mol. Microbiol..

[130] Janga S.C., Salgado H., Martínez-Antonio A. (2009). Transcriptional regulation shapes the organization of genes on bacterial chromosomes. Nucleic Acids Res..

[131] Llopis P.M., Jackson A.F., Sliusarenko O., Surovtsev I., Heinritz J., Emonet T., Jacobs-Wagner C. (2010). Spatial organization of the flow of genetic information in bacteria. Nature.

[132] Kuhlman T.E., Cox E.C. (2012). Gene location and DNA density determine transcription factor distributions in *Escherichia coli*. Mol. Syst. Biol..

[133] Fritsche M., Li S., Heermann D.W., Wiggins P.A. (2011). A model for *Escherichia coli* chromosome packaging supports transcription factor-induced DNA domain formation. Nucleic Acids Res..

[134] Hardy C.D., Cozzarelli N.R. (2005). A genetic selection for supercoiling mutants of *Escherichia coli* reveals proteins implicated in chromosome structure. Mol. Microbiol..

[135] Wu F., Japaridze A., Zheng X., Wiktor J., Kerssemakers J.W.J., Dekker C. (2019). Direct imaging of the circular chromosome in a live bacterium. Nat. Commun..

[136] Képès F., Jester B.C., Lepage T., Rafiei N., Rosu B., Junier I. (2012). The layout of a bacterial genome. FEBS Lett..

[137] Képès F. (2003). Periodic epi-organization of the yeast genome revealed by the distribution of promoter sites. J. Mol. Biol..

[138] Couturier E., Rocha E.P.C. (2006). Replication-associated gene dosage effects shape the genomes of fast-growing bacteria but only for transcription and translation genes. Mol. Microbiol..

[139] Norris V., Blaauwen T.D., Cabin-Flaman A., Doi R.H., Harshey R., Janniere L., Jimenez-Sanchez A., Jin D.J., Levin P.A., Mileykovskaya E. (2007). Functional taxonomy of bacterial hyperstructures. Microbiol. Mol. Biol. Rev..

