# Supplementary material for: Composition of Transcription Machinery and Its Crosstalk with Nucleoid-Associated Proteins and Global Transcription Factors
Source: Biomolecules. 2021 Jun 22;11(7):924. doi: 10.3390/biom11070924 (PMC8301835; doi:10.3390/biom11070924)
Supplement: Supplementary file 1 [file biomolecules-11-00924-s001.zip › biomolecules-1254538-supplementary.pdf]

Supplementary data

List of abbreviations used in the text

Supplementary Figures

Experimental procedure to Figure 2

Appendix

List of abbreviations used in the text:

RNAP – RNA polymerase

NAP- nucleoid associated protein

TRN- Transcriptional regulatory network

TF- transcription factor

TG- target gene

TCA –tricarboxylic acid cycle

CODO – coherent domain of transcription

ASF – alternative sigma factors

αCTD - C-terminal domain of the RNAP α subunit

IMPDH - inosine 5'-monophosphate dehydrogensase

guaB – gene encoding inosine 5'-monophosphate dehydrogensase

suhB – gene encoding inositol-1-monophosphatase

kdpA - gene encoding K^+ transporting P-type ATPase subunit KdpA

kdpB - gene encoding the largest subunit of a potassium ion importing P-type ATPase KdpB

gltP - gene encoding a proton-dependent glutamate and aspartate transporter

suhB - gene encoding inositol 1-monophosphatase involved in ribosomal RNA maturation

opgG – gene encoding osmoregulated periplasmic glucans biosynthesis protein G

yrdD - gene encoding putative type 1 DNA topoisomerase

parC - gene encoding DNA topoisomerase IV subunit A

parE - gene encoding DNA topoisomerase IV subunit B

FIS –factor for inversion stimulation encoded by *fis* gene

IHF – integration host factor encoded by *ihfA* and *ihfB* genes

HU- histone-like ptotein from strain U93 encoded by *hupA* and *hupB* genes

H-NS – histone like nucleoid structuring protein, encoded by *hns* gene

Lrp – leucine responsive protein encoded by *lrp* gene

Dps - DNA-binding protein from starved cells encoded by *dps* gene

CRP - catabolite repressor protein encoded by *crp* gene

Fnr - fumarate and nitrate Reduction regulator encoded by *fnr* gene

RpoD – major $sigma^{70}$ factor encoded by *rpoD* gene

RpoS – stationary phase $sigma^{38}$ (aka $sigma^{S}$) factor encoded by *rpoS* gene

Composition of transcription machinery and its crosstalk with nucleoid-associated proteins and global transcription factors

Georgi Muskhelishvili[1], Patrick Sobetzko[2], Sanja Mehandziska[3,4], and Andrew Travers[5,6]

[1]Agricultural University of Georgia, School of Natural Sciences, David Aghmashenebeli Alley 24, 0159 Tbilisi, Georgia
[2]Philipps-Universität Marburg, LOEWE-Zentrum für Synthetische Mikrobiologie, Department of Chromosome Biology, Hans-Meerwein-Straße, 35043 Marburg, Germany
[3]Jacobs University Bremen, School of Engineering and Science, Campus Ring 1, D-28759 Bremen, Germany
[4] Current address: ZAN MITREV CLINIC, Str. Bledski Dogovor no.8, Skopje, 1000, Macedonia
[5]MRC Laboratory of Molecular Biology, Francis Crick Avenue, Cambridge Biomedical Campus, Cambridge, UK, CB2 0QH.
[6]Department of Biochemistry, University of Cambridge, Tennis Court Road, Cambridge, UK, CB2 1GA.

Supplementary data

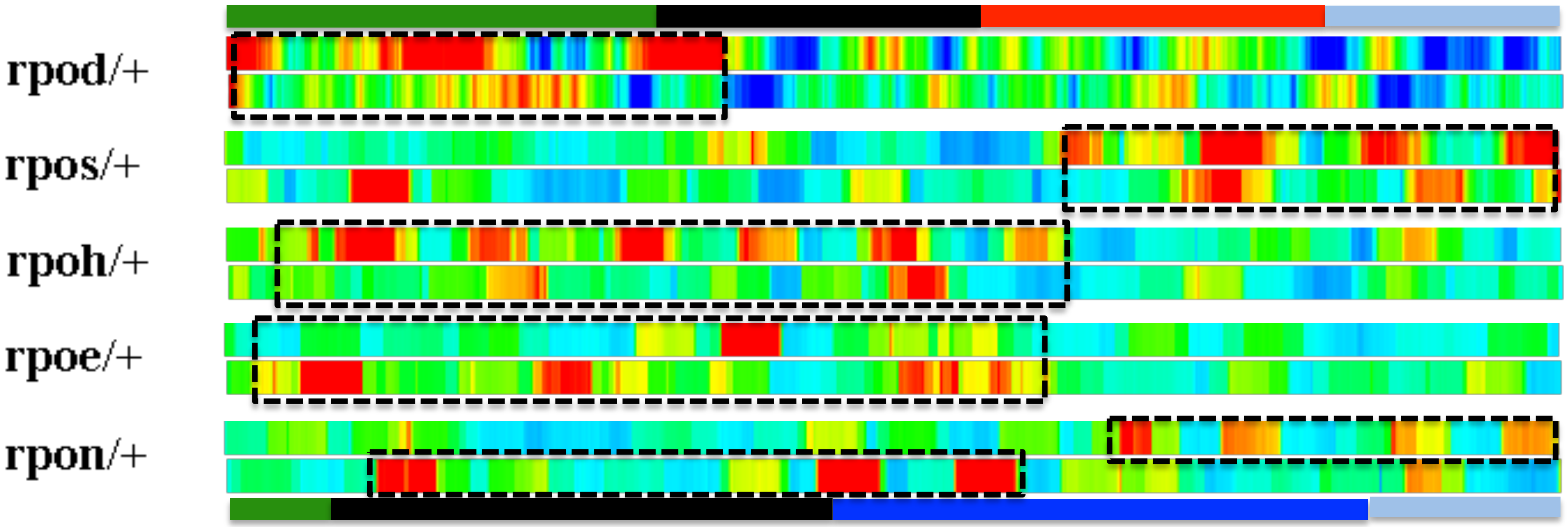

Suppl.Figure 1
Genomic organization of the s factor regulons. The circular chromosome is divided into the right (top) and left (bottom) replichores. The origin of replication is situated on the left, the terminus region on the right. The red and blue colors respectively indicate a significant enrichment (Z score >2) and depletion (Z score <-2) in relative frequency of the regulon genes. The chromosomal macrodomains are aligned to the right (at the top) and left (at the bottom) replichores and color-coded: green – Ori, red-Right; light blue - Ter; blue – Left; black – non-structure right (top) and non-structured left (bottom). Chromosomal regions showing relative enrichment for regulon genes are highlighted by dashed rectangles.

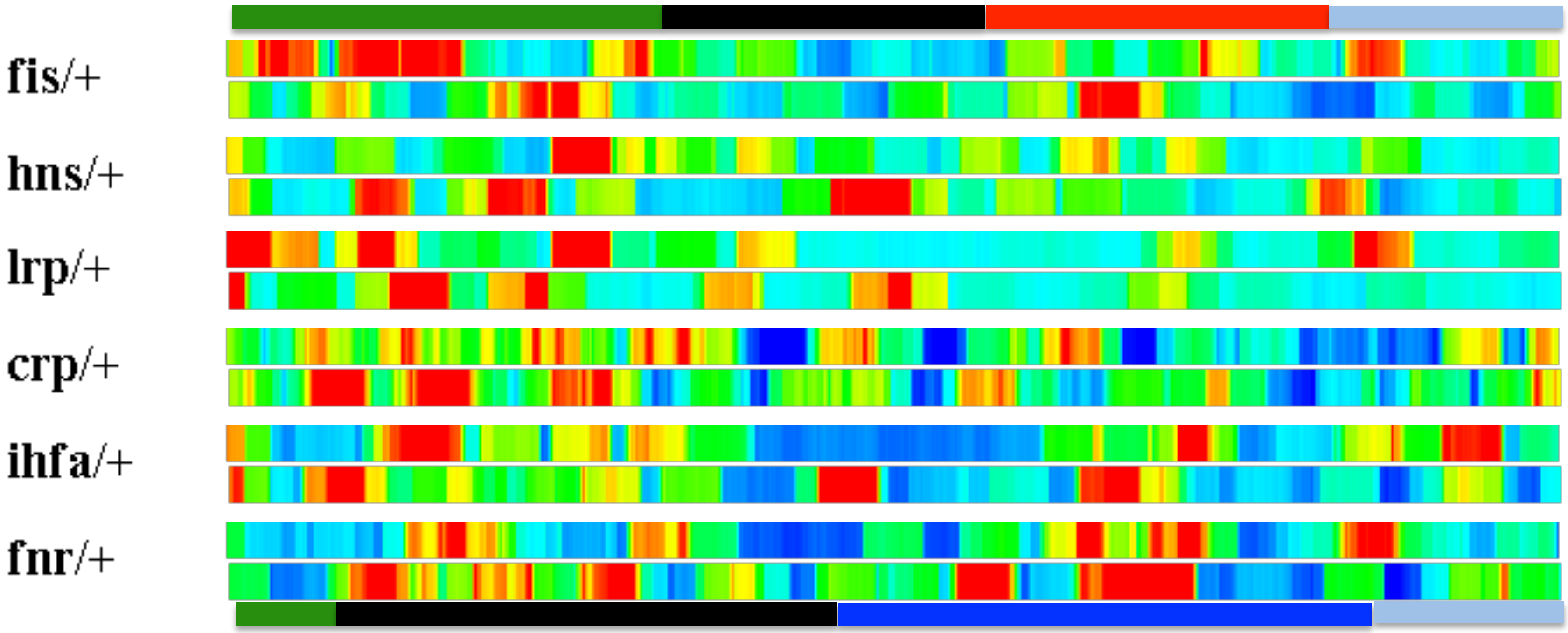

Suppl.Figure 2

Genomic organization of the NAP regulons. The circular chromosome is divided in the right replichore (top) and left replichore (bottom). The origin of replication is situated on the left, the terminus region on the right. The red and blue colors respectively indicate a significant enrichment (Z score >2) and depletion (Z score <-2) in relative frequency of the regulon genes. The chromosomal macrodomains are color-coded as in Suppl. Fig.1.

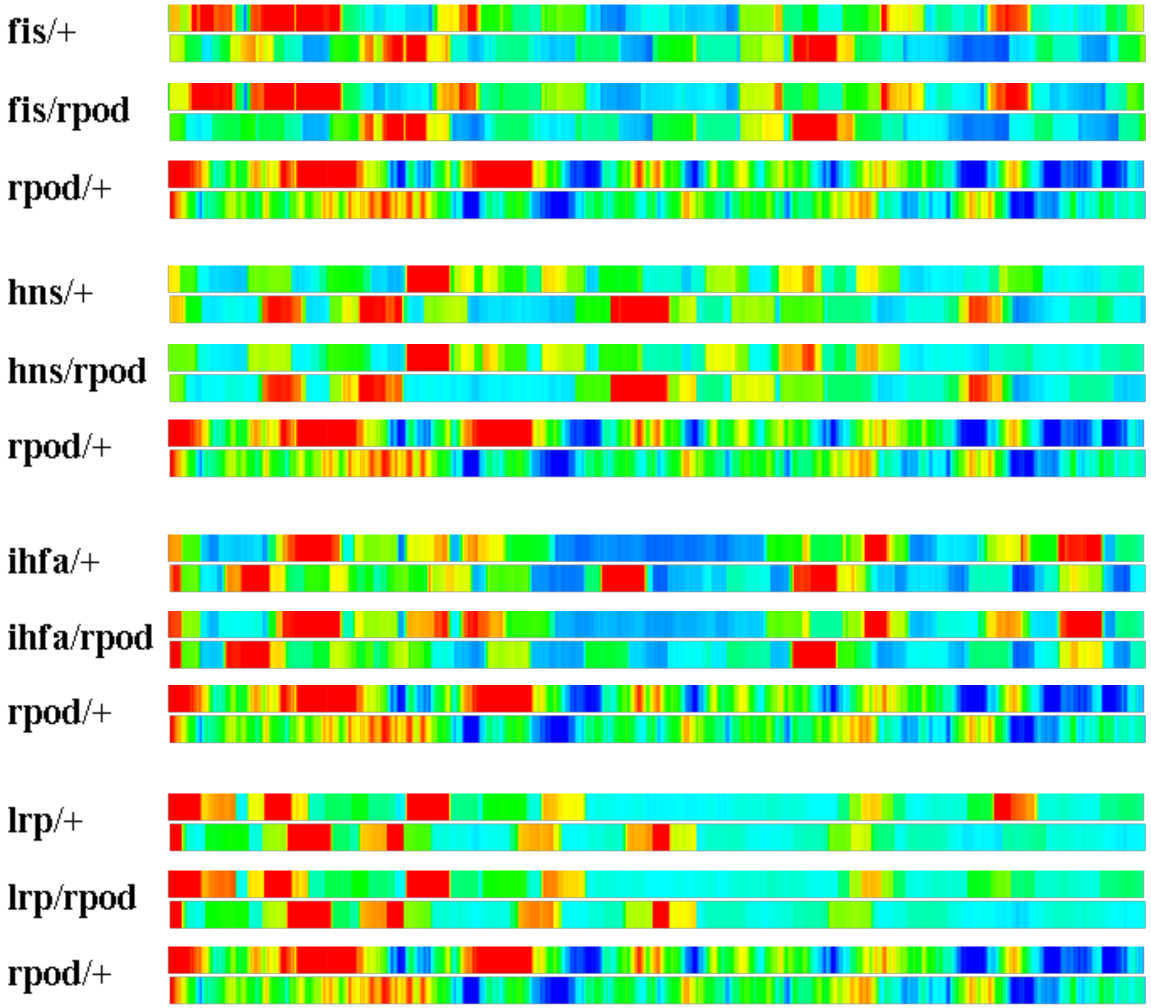

Suppl.Figure 3.
Comparison of the genomic organisation of NAP regulons and NAP/RpoD couplons. The circular chromosome is divided in the right replichore (top) and left replichore (bottom). The origin of replication is situated on the left, the terminus region on the right. The red and blue colors respectively indicate a significant enrichment (Z score >2) and depletion (Z score <-2) in relative frequency of the regulon or couplon genes. Note that the couplon patterns largely coincide with those of the NAP regulons.

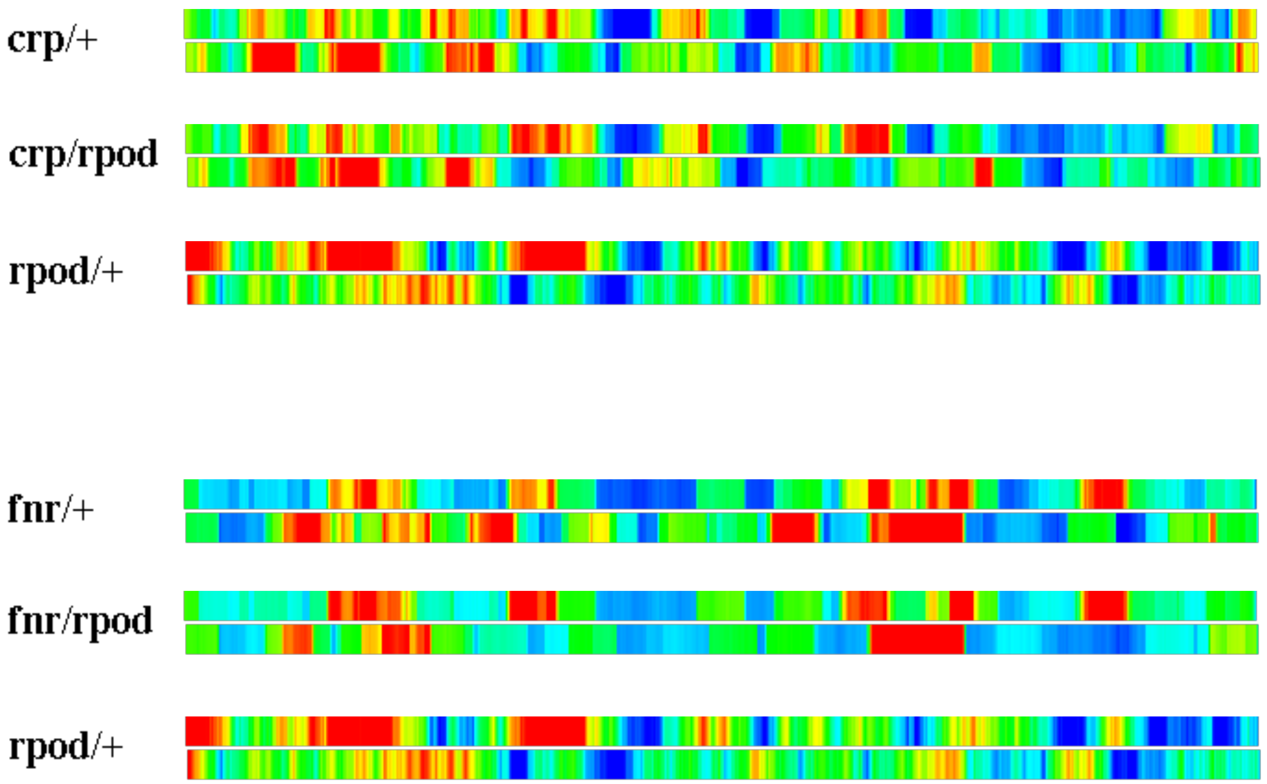

Suppl.Figure 4.
Comparison of the genomic organisation of global TF (CRP and Fnr) regulons and their cognate RpoD couplons. The circular chromosome is divided in the right replichore (top) and left replichore (bottom). The origin of replication is situated on the left, the terminus region on the right. The red and blue colors respectively indicate a significant enrichment (Z score >2) and depletion (Z score <-2) in relative frequency of the regulon or couplon genes. Note that the couplon patterns largely coincide with those of the global TF regulons.

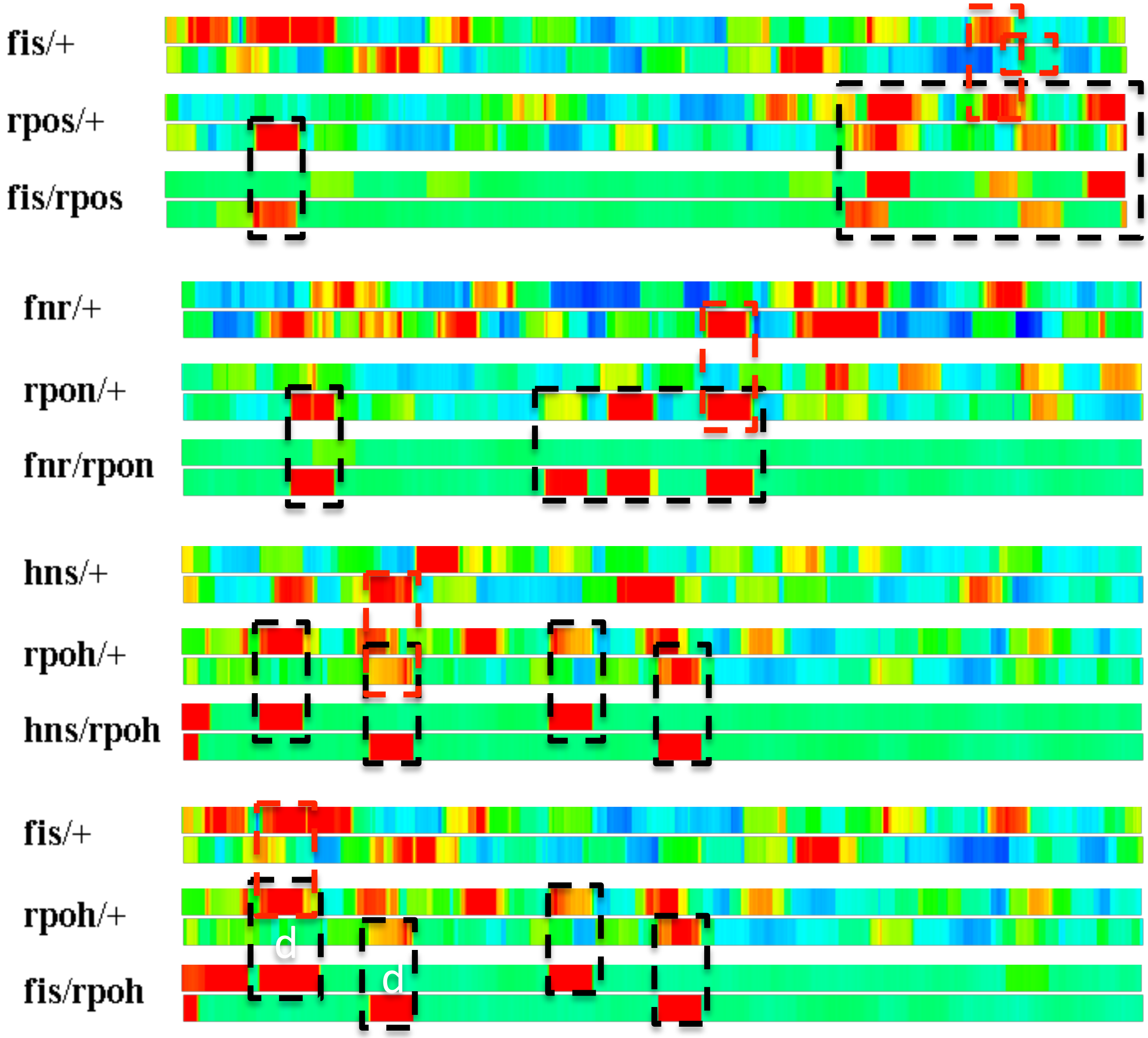

Suppl.Figure 5.
Comparison of the genomic organisation of the NAP and the ASF (RpoN and RpoH) regulons and cognate couplons as well as the Fnr and RpoN regulons and Fnr/RpoN couplons. The circular chromosome is divided in the right replichore (top) and left replichore (bottom). The origin of replication is situated on the left, the terminus region on the right. The red and blue colors respectively indicate a significant enrichment (Z score >2) and depletion (Z score <-2) in relative frequency of the regulon or couplon genes. The similarities between the spatial organization of ASF regulons and the NAP/ASF and Fnr/RpoN couplons are highlighted by black dashed retangles.

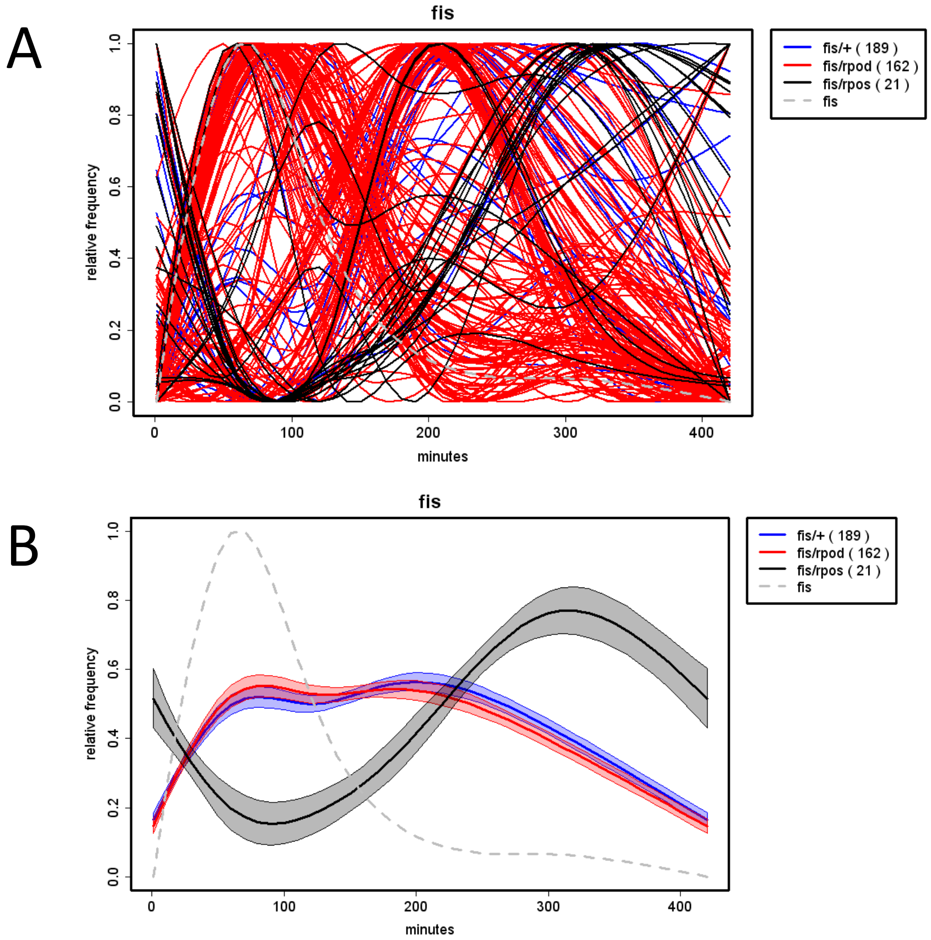

Suppl.Figure 6

Determination of the temporal expression of regulons and couplons on the example of FIS regulon and, the FIS/RpoD and FIS/RpoS couplons **A.** Normalized expression patterns of the FIS regulon (blue curves) and those of FIS/RpoD couplons (red curves) and FIS/RpoS couplons (black curves). Corresponding gene numbers are indicated in the inset. The different curves were normalized to [0;1] to compare them in one plot. **B**. The averaged expression patterns of the FIS regulon and FIS/RpoD, FIS/RpoS couplon genes shown in **A**. The envelopes of the curves indicate the standard deviation at 10% random remapping of the expression patterns to genes. The temporal expression pattern of the *fis* gene is indicated by dashed grey line (data from ref. 29).

Supplementary Table 1| Protein identities of the proteins discovered in the RNAP supramolecular complex (Figure 1) and their functions. Protein functions quoted from Keseler et al., 2013 (EcoCyc www.ecocyc.org) and references therein.

Band No.	Identified proteins	Standard MS-MS Score	Peptide mass tolerance	Gene Name	Function
**1**	RNAP β subunit	101	± 120 ppm	***rpoB***	RNAP subunit<br>Important for catalytic activity. Binds DNA and forms active site with β' subunit.
**2**	DNA Gyrase subunit A	119	± 150 ppm	***gyrA***	Type II topoisomerase. Carries out ATP-dependent supercoiling of chromosomal DNA, as well as potentially being involved in decatenation of newly synthesized chrosomal and plasmid DNA.
**2a**	DNA Gyrase subunit A	78	± 100 ppm	***gyrA***	Same as above.
**3**	30S ribosomal subunit protein S1	117	± 250 ppm	***rpsA***	Component of 30S Ribosome.<br>Required for translation of most mRNAs (essential).
**4**	Glucansbiosynthesis protein G	129	± 300 ppm	***mdoG***	
**5**	Transcriptional termination factor Rho	80	± 150 ppm	***rho***	A hexameric helicase required for termination of RNA transcription. Binds to nascent RNA transcripts and halts transcription by displacing the template DNA.
**6**	RNAP α subunit	114	± 50 ppm	***rpoA***	RNAP subunit<br>Assembles in RNAP as dimers via amino-terminus. The carboxy-terminal domain of RpoA is involved in antitermination, rho-dependent termination, and is a target for interactions with transcription.
**7**	30S ribosomal subunit protein S2	50*	± 110 ppm	***rpsB***	Component of 30S Ribosome.<br>Required for binding of S1 to the ribosome (essential).
**8**	30S ribosomal subunit protein S3	123*	± 100 ppm	***rpsC***	Component of 30S Ribosome.<br>Surface-accessible and located on the head of the 30S subunit. It can be cross-linked to rRNA and nascent peptides as well as to IF3. It is the

					main target of modification by reaction with pyridoxal phosphate.
**9**	30S ribosomal subunit protein S4	32*	± 50 ppm	***rpsD***	Component of 30S Ribosome. Functions in the assembly of the 30S ribosomal subunit, the mRNA helicase activity of the ribosome, the regulation of translation of a subset of ribosomal proteins, and transcription antitermination of rRNA operons.
**10**	Inosine 5'-monophosphate dehydrogenase (IMPDH)	89	± 150 ppm	***guaB***	Catalyzes reaction: IMP + NAD^+ + H2O <=> XMP + NADH + H^+ First committed, rate-limiting step in de novo guanine nucleotide biosynthesis in most organisms.
**11**	Isocitrate dehydrogenase	69	± 150 ppm	***icd***	Catalyzes reaction: D-threo-isocitrate + $NADP^+$ <=> 2-oxoglutarate + CO_2 + NADPH Is regulated by phosphorylation/dephosphorylation. The modulation of this key enzyme activity enables *E. coli* to make rapid shifts between TCA and glyoxalate bypass pathways.
**12**	rRNA large subunit methyltransferase	78	±250 ppm	***rlmA***	Responsible for methylation of 23S rRNA at the N1 position of the G745 nucleotide. Its activity is required for wild-type translation and cell growth. The methylated base is estimated to reside near the peptide exit channel of the ribosome.
**13**	Acetyl-CoA carboxylase carboxyl-transferase	96	± 100 ppm	***accA***	Catalyzes reaction: ATP + acetyl-CoA + a carboxylated-biotinylated [BCCP dimer] + H_2O <=> malonyl-CoA + a biotinylated [BCCP dimer] + ADP + phosphate + H^+ The enzyme is part of the acetyl-CoA carboxylase complex, catalyzing the second half-reaction, the carboxylation of acetyl-CoA to form malonyl-CoA (essential).
**14**	Inositol-1-monophosphatase	102	± 60 ppm	***suhB***	Catalyzes reaction: glycerol 2-phosphate + H_2O <=> glycerol + phosphate Exists as a mixture of monomers and dimers in solution. The monomeric form can interact with RNA polymerase, and the ability of mutants to bind to RNA polymerase correlates with their ability to rescue the cold-sensitive growth defect of an *suhB* mutant.

**15**	30S ribosomal subunit protein S21	76	± 60 ppm	***rpsU***	Component of 30S ribosome. Interacts with 16S rRNA. Also crosslinks to the 4.5S RNA of the signal recognition particle.<br>It is required for full activity of translation initiation. S21 was also shown to crosslink to IF3.
**16**	30S ribosomal subunit protein S13	107*	± 150 ppm	***rpsM***	Component of 30S ribosome.<br>Plays a key role in ribosomal subunit association and the fidelity of translocation. Contacts the L5 protein of the large ribosomal subunit.
**17**	30S ribosomal subunit protein S9	81	± 250 ppm	***rpsI***	Component of 30S ribosome.<br>Crosslinks to domains 3 and 4 of 16S rRNA and increases protection against ribunuclease digestion of the hairpin loop 41 near the 3' terminus of 16S rRNA by a mixture containing S7, S14, and S19. S9 crosslinks to tRNA at the ribosomal P site and weakly to tRNA at the A site. S9 is one of the subunits required for the ribosome-dependent GTPase activity of EF-G.
**18**	30S ribosomal subunit protein S7	46*	± 60 ppm	***rpsG***	Component of 30S ribosome.<br>S7 binds to 16S rRNA. It is required for assembly of S9, S13, S19, S10, S14, and S3 ribosomal subunits.<br>S7 crosslinks to the anticodon loop of tRNAs, indicating its location close to the decoding site of the ribosome. It also crosslinks to mRNA and to IF3.<br>S7 is a translational feedback repressor, regulating the synthesis of itself and the other genes in its operon: S12, S7, EF-G and possibly EF-Tu.

*MudPIT scoring

Experimental procedure for Figure 2:

Strain Growth and Protein Extraction *E. coli CSH50* wild type and NAP mutant strains were grown in 3l dYT medium (1.6% tryptone 1% yeast extract, 0.05% NaCl) in BIOSTAT Bplus fermentor Sartorius AB, Göttingen, Germany). Conditions were set to 37°C, pH 7.5, 500 rpm, and pO2 of 100% at the start of the growth, and carefully controlled throughout the growth. At mid-exponential phase (OD600 ~0.5), for crude extracts 4 × 50 ml samples were collected, 5 ml of 10 × Killing buffer (50mM Tris-HCl pH 7.5, 15 mg/ml sodium azide, 0.6 mg/ml chloramphenicol) was added to them, vortexed, and then centrifuged at 10 000 rpm for 10 min at 4°C in a Beckman Coulter centrifuge, C1050 rotor (Beckman Coulter GmbH, Germany). Pellets were washed twice with Phosphate-Buffered Saline (PBS), supernatant was discarded, and pellets were frozen and kept at -20°C until further use. For RNAP extracts 3 × 500 ml samples from the same culture were centrifuged for 20 min at 4200 rpm in a Multifuge 3 S-R Heraus centrifuge (Schnakenberg Medizin -& Labortechnik GmbH, Germany). Supernatant was discarded, pellets were washed twice with PBS and kept at -20°C until further use.

Cell pellets were thawed on ice and resuspended in 2 ml of ice-cold sonication/equilibration buffer (10 mM Tris-HCl pH 8.0, 10 mM MgCl2, 0.1 mM EDTA, 0.1 mM DTT, 5% (v/v) Glycerin, 230 mM NaCl), supplemented with freshly added 1× Protease Inhibitor Mix HP PLUS (SERVA Electrophoresis, Heidelberg, Germany), 70U/ml Benzonase (Sigma-Aldrich Chemie GmbH, Steinheim, Germany) and 2g glass beads (diameter 0.45 µm - 0.50 µm) (B.Braun Biotech International, Melsungen, Germany). Lysis was performed using 0.5 s bursts at 100% amplitude with a UP 200H ultrasonication processor (Dr. Hielscher GmbH, Germany) for 30 s, followed by 1 min incubation on ice, and vortexing for 30 s. Five to seven sonication cycles were carried out. Sonicated sample was incubated for 1h on ice, suspension was transferred into 2ml microtubes and cleared by centrifugation at 13000 × g for 30 min at 4°C. Supernatant was transferred to fresh microcentrifuge tube and centrifuged for another 10 min, to remove residual cell debris. Protein extract was transferred to fresh tube again and kept on ice before purification.

Purification of the RNAP Complex: The RNAP supramolecular complex was purified from the cleared whole-cell extracts of exponentially growing cells (OD600 ~0.5) by high-performance liquid chromatography (HPLC), using ÄKTApurifier 10 system (GE Healthcare, Munich, Germany). The cleared cell extract was loaded onto a HiTrap Heparin 1ml column (GE Healthcare, Munich, Germany), equilibrated with 10 column volumes (CV) of sonication/equilibration buffer. Protein was eluted by gradient purification with increasing NaCl concentration from 230 mM to 1 M, at a flow rate of 0.5 ml/min. 1 ml protein fractions were collected.

Peak was eluted at ~530 mM NaCl concentration. Peak containing fractions were then concentrated using an Amicon Ultra-4 100K centrifugal filter (Millipore, Schwalbach, Germany) at 4°C and 3200 × g for 20 minutes in a swing-rotor centrifuge. Afterwards, 2 ml of equilibration buffer with no NaCl was added and the centrifugation step was repeated. Once the sample volume decreased to ~200 µl, 2 × 1 ml of RNAP storage buffer (20mMTris-HCl pH 7.5, 100 mM NaCl, 0.1 mM EDTA, 1 mM DTT, 50% glycerol v/v) were passed through the column. Centrifugation step varies according to the sample concentration (~1 – 2 h). Once ~250 µl of sample was left in the filter, it was transferred to 1.5 ml ice-cold microcentrifuge tube. The protein concentration was then determined using a Pierce BCA ® Protein Assay Kit (Thermo Scientific, Darmstadt, Germany) following the procedure in the manufacturer's instruction manual.

Western blotting: 10 µg of protein from crude cell extracts or purified RNAP fractions were premixed with 4 × loading dye (150 mM Tris HCl pH 6.8, 2.8 M □-mercaptoethanol, 3% SDS, 20% glycerol, bromophenolblue), denatured by boiling at 95°C for 10 min, and loaded on 8%-15% gradient SDS-PAGE gel. In addition, 7.5 µl of Color-Plus™ Prestained Protein Marker (New England Biolabs GmbH, Frankfurt, Germany) were used for small gels. Electrophoresis was carried out at 80 V for 20 min followed by 120 V for 2 h. Proteins were then transferred onto a Whatman™ Protran® BA83 nitrocellulose membranes (GE Healthcare, Munich, Germany) via semi-dry blotting. Primary antibodies used: Monoclonal mouse IgG antibodies raised against E. coli RNAP □' and σ factors were purchase from Neoclone, Madison, MA, USA and used as 1:1000 dilutions in 1% milk in TBST. Secondary antibodies used: 1:1000 dilution of AP-linked goat anti-mouse IgG (Cell SIgnalling Technology, Beverly, MA, USA).

Quantitative western blot analysis was done with ECF^{TM} kit (GE Healthcare, Munich, Germany) and detected with a Fuji-Film FLA-3000 Fluorescent Image Analyzer. The BAS Reader v3.14 software was used for scanning using the following parameters: 473 nm laser, O580 filter, resolution of 16 bit per pixel, 100 µm pixel size, and 1000 sensitivity.

A Study of *RNA* Polymerase and Nucleoid Associated Protein composition

in *Escherichia coli* Stringent Mutants

by

Steffi Jimmy

A thesis submitted in partial fulfilment
of the requirements for the degree of

Master of Science in Molecular Life Sciences

Approved by the thesis Committee:

____

(Prof. Dr. Georgi Muskhelishvili)

____

(Prof. Dr. Sebastian Springer)

____

(Prof. Dr. Matthias Ullrich)

Date of Defence: August 30, 2013

School of Engineering and Science

Abstract

There must exist a structural coupling between the transcription machinery (RNA Polymerase), DNA topology and the metabolic energy in *E.coli* bacteria which renders the robustness to the bacterial system. The above three parameters were studied in the wild type, Δ*dksA*, *rpoB114*, *rpoB3370* and *rpoB3449* strains along different stages of their growth cycle. Western blot analysis results showed a clear modulation in the σ factor composition of RNA polymerase (RNAP) in the mutants in contrast to the wild type strain during the growth cycle. Also, the nucleoid associated protein (NAP) composition also varied radically in these strains. Higher concentrations of H-NS and IHF proteins were observed in the *rpoB* mutants which may contribute to the changes in DNA topology. The DNA in *rpoB* mutants is less relaxed in the early and late stationary growth phases when compared to the wild type cells. BN–PAGE and subsequent MALDI–TOF studies revealed the presence of certain functionally relevant metabolic enzymes and several ribosomal proteins (part of 30S subunit of the ribosome) in the RNAP complex. Presence of ribosomal proteins shows the coupling between transcription and translation processes and co-operation between the RNAP and ribosome of an appropriate configuration.Presence of metabolic enzymes points to the fact that the chromatin is in constant contact to the metabolic network and any changes in the metabolic energy would directly affect the DNA topology and in turn the RNAP subunit composition and the NAP composition.

Contents

1 Introduction **1**

2 Materials and Methods **11**

2.1 RNAP σ Factor and NAP Composition 11

2.1.1 Bacterial Strains 11

2.1.2 Bacterial Growth 11

2.1.3 Protein Extraction for SDS-PAGE 12

2.1.4 Protein Concentration Quantification 12

2.1.5 SDS-PAGE 12

2.1.6 Western Blotting 12

2.1.7 Phenotypic Assay 13

2.2 DNA Topology 13

2.2.1 Bacterial Growth for DNA Topology Analysis 13

2.2.2 High Resolution Gel Electrophoresis 14

2.3 RNAP Supramolecular Composition 14

2.3.1 Protein Purification through HPLC 14

2.3.2 Blue Native Polyacrylamide Gel Electrophoresis (BN-PAGE) . . 15

2.3.3 MALDI-TOF 16

3 Results and Discussion **19**

3.1 Expression of σ Factors and NAPs in *DksA* and Stringent Mutants . . . 19

3.2 Changes in DNA Topology 25

3.3 Changes in RNAP Supramolecular Composition 30

4 Conclusion and Outlook **35**

References **39**

Acknowledgment **45**

1 Introduction

The chromosomal DNA of *Escherichia coli* bacteria is about 4.6 Mbp in size and to fit this DNA into a bacterial cell, it must be compacted to at least a 1000 fold. Bacterial genomes are organized and compacted by several chromatin proteins called as *Nucleoid associated proteins* (NAPs) and by various physical mechanisms. These proteins were discovered in 1970s and since then the spatial organization of bacterial DNA has been vastly studied upon. Bacterial genome organization can be described in terms of different length scales; NAPs modulate the DNA supercoiling on a nanometer scale, the genome folds into loops on the order of 10 kbp in size on an intermediate scale and the genome gets divided into independent domains on the order of 1 Mbp in size on a micrometer scale [1].

The Core RNAP Enzyme

RNAP enzyme has been found to be evolutionarily conserved in sequence, structure and function in all living organisms but the only difference that was observed was the subunit composition [2] [3]. The bacterial core RNAP (E) has a subunit composition of $\alpha_2\beta\beta'\omega$ ($\approx$400 kDa complex). β' is the largest subunit followed by β subunit and the active centre of the RNAP (responsible for RNA synthesis) is shared by these two subunits. α subunit is the third largest subunit and helps in the interaction of RNAP to the promoter region of the target DNA where transcription needs to be carried out. Each α subunit consists of two domains: α-NTD (N-terminal domain) and α-CTD (C-terminal domain) joined by a flexible linker [4]. α-NTD consists of information for the assembly of RNAP and α-CTD contains information for interaction with the promoter region. ω subunit is the smallest subunit and is responsible for the stability of the assembled RNAP [4]. Although core RNAP enzyme consists of all catalytic information which is required for transcription, it is not able to bind to the promoter DNA with much efficiency [5]. For RNAP to initiate transcription at a specific promoter region, it must bind to another important subunit called the sigma (σ) factor which in turn forms the

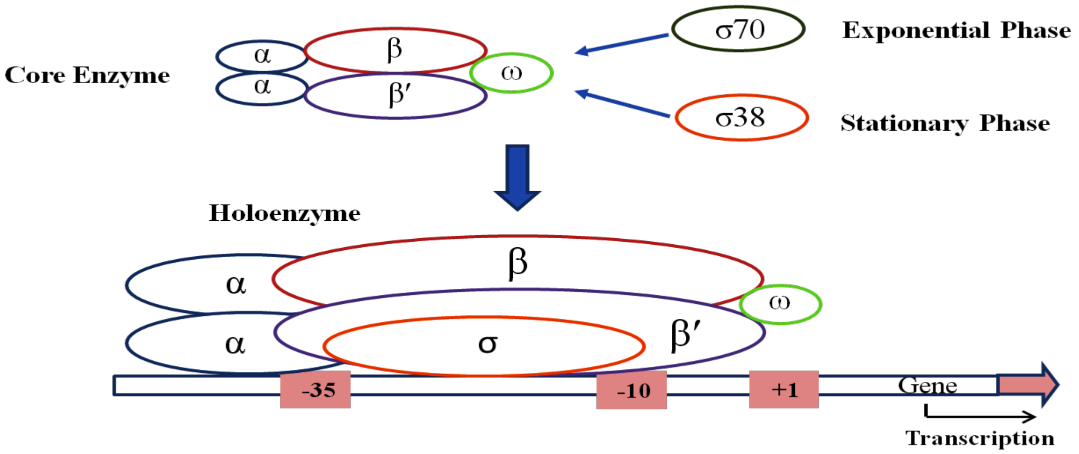

Figure 1.1: *A schematic representation of RNAP subunit composition.*

functionally important RNAP holoenzyme (Eσ). The σ factor along with the β and β' subunits regulate the opening and closing of the channel in the catalytic centre of RNAP during transcription. This interaction helps in the addition of nucleotides into the active site channel of RNAP and thereby transcribing the required gene (fig. 1.1).

The Sigma Factors

The most vital component of RNAP which recognizes and binds specific promoter regions and drives RNAP to initiate the process of transcription is the σ factor. It has a pivotal role in the functioning of RNAP holoenzyme. Sigma factors are involved in various functions like: promoter melting near the transcription initiation site, interaction with transcription activators, inhibition of non-specific interactions and regulation of transcription by releasing the promoter from RNAP after completion of initiation [6].

Different sigma factors are activated according to the change in environmental conditions. Under a specific environmental condition, a specific sigma factor would be activated which in turn would recognize a specific promoter DNA and increase the transcription of those special genes. There are seven sigma factors in *E.coli* which help in transcription of different genes: σ^{70}(*RpoD*) - the "housekeeping" sigma factor that transcribes most genes in growing cells and keeps essential genes and pathways operating, σ^{19} (*FecI*) - the ferric citrate sigma factor which regulates the *fec* gene for iron transport, σ^{24} (*RpoE*) - the extracytoplasmic/extreme heat stress sigma factor, σ^{28} (*RpoF*) - the flagellar sigma factor, σ^{32} (*RpoH*) - the heat shock sigma factor and is turned on when exposed to heat, σ^{38} (*RpoS*) - the starvation/stationary phase sigma

factor and σ^{54} (*RpoN*) - the nitrogen-limitation sigma factor [7].

σ^{70} Holoenzyme

Known as the primary sigma factor, σ^{70} is essential for transcription in exponentially growing *E.coli* cells and can be replaced by alternative sigma factors which express genes involved in a number of diverse functions like stress response, mobility development, iron uptake, morphological changes, etc. σ^{70}- RNAP holoenzyme (Eσ^{70}) binds to promoters with conserved recognition motifs at -35 (TTGACA) and -10 (TATAAT) regions upstream of the transcription start site at position +1 [5]. As soon as σ^{70} binds to the promoter region, a transition takes place from closed complex to an open complex (isomerisation) and various regulatory proteins help in the regulation of this transition. DNA strands separate at the -10 consensus motif and the template strand is loaded onto the active site channel of RNAP for RNA synthesis [3].

σ^{S} Holoenzyme

σ^{S} encoded by *rpoS* gene is an alternative sigma factor which acts as the major regulator of stress response in *E.coli* bacteria [13]. It is involved in regulating expression of various stringent response genes related to stress resistance, virulence, metabolism, cell morphology, lysis, etc. [14] [15]. Eσ^{S} holoenzyme recognizes almost similar consensus promoter sequences as Eσ^{70} holoenzyme [16]. When nutrient limiting conditions arise during starvation or oxidative stress, σ^{S} leads RNAP to the genes important for cell survival that are recognized by σ^{70} during exponential growth. Therefore, a distinction between these identical promoter sequences is of prime importance. Primarily, the degree of supercoiling can affect the specific promoter recognition by Eσ^{S} as Eσ^{S} holoenzyme prefers relaxed form of DNA to the negatively supercoiled one preferred by Eσ^{70} holoenzyme [17]. Secondly, a number of NAPS like H-NS and IHF can modulate promoter recognition by either blocking or promoting the recognition by Eσ^{70} or Eσ^{S} [18]. In addition, σ^{S} production and its activity are affected by molecules like ppGpp and DksA during transition from exponential to starvation phase [19].

The Sigma Cycle

Although the bacterial RNAP core enzyme consists of all the necessary determinants to start the process of transcription, it is incapable of promoter-specific initiation without the σ factors. The binding of the σ factor to the RNAP, followed by recognition of promoter sequence and melting of the DNA to expose the transcription start site to the active site of RNAP, is a very complex process and is described as a *Sigma Cycle*.

During the sigma cycle, σ binds to the RNAP and helps in initiation of transcription and then dissociates when a stable elongation complex (EC) is formed [20]. Once the transcription is complete, RNAP releases the DNA and RNA, and can bind to a new or different sigma factor and begin a new cycle of transcription of a different set of genes [21]. With the help of σ cycle, the cell is able to adapt to the change in external conditions by rapidly changing transcription patterns by using different σ factors with varying promoter specificities to regulate the gene expression. σ^{70} is present in relatively high quantities as it regulates the transcription of housekeeping genes and other σ factors get activated when there is a certain type of external signal. In this way transcription is regulated with very high efficiency with the help of σ cycle and the change in external conditions is relayed into the core of the cell with high competence.

Nucleoid-Associated Proteins (NAPs)

Unlike eukaryotes, bacteria do not contain any histone proteins to wrap and compact their genome. Instead a class of proteins is present that alter the shape of the DNA and make it more compact and additionally affect transcription. These proteins are referred to as *Nucleoid-Associated Proteins* because of their location and function. They possess DNA-binding properties and ability to change the DNA topology by bending, wrapping or bridging it. These properties help them to affect transcription either positively or negatively by affecting the architecture of the bacterial genome [23]. NAPs not only affect the structure of the DNA but also regulate gene expression and they are classified as follows:

H-NS: Histone-like Nucleoid-Structuring protein is referred to as the guardian of the genome. It constrains supercoils in DNA and also reinforces duplex interwinding in plectonemically supercoiled DNA [24]. It also forms bridges with DNA which allows it to not only change the shape but also regulate the gene expression [25]

HU: Heat Unstable protein is referred to as regulator of DNA flexibility. It affects the flexibility of the bacterial DNA by bending the duplex and in turn reducing the stiffness of the DNA over short distances (when present in low concentrations) [26]. This helps DNA loop formation which affects the gene regulation [27]. It has been previously observed that HU has a role in expression of genes related to central metabolism and respiration [28]. It also interacts with topoisomerase I causing a change in the DNA superhelicity and coiling [29].

IHF: Integration Host Factor is a DNA binding protein that introduces a U-turn into the DNA and binds a well conserved DNA sequence [30]. It consists of an α-subunit and β-subunit and forms $\alpha\beta$ heterodimer [31]. It helps interaction between

regulatory proteins and RNAPb [32]. It bends the DNA by utilizing the energy that was present in the twist in the negatively supercoiled DNA. It also affects initiation of replication at the origin [33]. Therefore, not only does IHF have a role in structuring of the genome, but also in chromosome replication and DNA rearrangement and the most important being the role in transcription.

Fis: Factor for Inversion Stimulation bends the DNA at its binding site and prefers to bind to sequences that are located at transcription promoter regions [34]. Fis can act in two ways: either by causing an obstruction at the target promoter for the RNAP and thereby causing repression or by mediating the transition from closed complex to an open complex (isomerisation) and activating transcription [35]. Fis also works by binding at negative supercoils and preserving the DNA writhe [36]. It regulates the expression of two major topoisomerases: DNA gyrase and DNA topoisomerase I. These functions render Fis to influence the DNA supercoiling globally and thereby affecting the promoters at stable RNA genes [38].

Stringent Response

Every organism needs to respond to the changes in its environment in order to survive. In response to environmental changes, species have evolved in such a way that their intracellular and intercellular regulatory systems could adapt to such changes. The most important regulatory system in bacteria is the *Stringent Response* [39], which was discovered over 40 years ago in *E.coli*. Stringent response in bacteria consists of pppGpp (Guanosine $5'$–triphosphate $3'$–diphosphate) and ppGpp (Guanosine $5'$–diphosphate $3'$–diphosphate) as its main components, which help in mediating the signals of changes in environment and act as secondary messengers. In nutrient limiting conditions, intracellular level of ppGpp increases [39] and this affects several vital processes like transcription and translation by directly binding to the RNAP, thereby altering the transcription profile. Thus, when the nutrient availability gets limited during the stationary phase of growth cycle, stringent response is activated which modulates the trancription levels of several genes.

When growth conditions become limiting, *E.coli* cells modulate their gene expression profile from supporting growth in exponential phase to improved survival in stationary phase. This switch is brought about by accumulation of alarmones (p)ppGpp. During starvation, amino acids become limiting which causes uncharged tRNAs to bind to ribosomal A site which in turn signals ribosome-associated *RelA* to synthesize ppGpp [40]. Now comes the role of a protein called DksA, which helps ppGpp to bind to the secondary channel of RNAP near the active site. This causes immediate

termination of trancription of stable RNAs (ribosomal and transfer RNAs). Ribosomal protein gene expression is also controlled by rRNA levels and thus, translation process also gets affected. Moreover, several amino acid biosynthesis genes have been shown to be up-regulated at the onset of stationary phase by ppGpp with the help of DksA.

Stringent Polymerases

RNAP mutants (point mutations in β subunit) have shed more light on the coordinated regulation of stringently controlled genes. It was observed that these mutants have an altered interaction with the stringently controlled promoters [41]. They behave as *Stringent Polymerases* even in absence of stringent response in vivo and activate expression of several genes which normally require the stringent response [41]. A study by Zhou and Jin in the year 1998 on RNA Polymerase mutants (used in

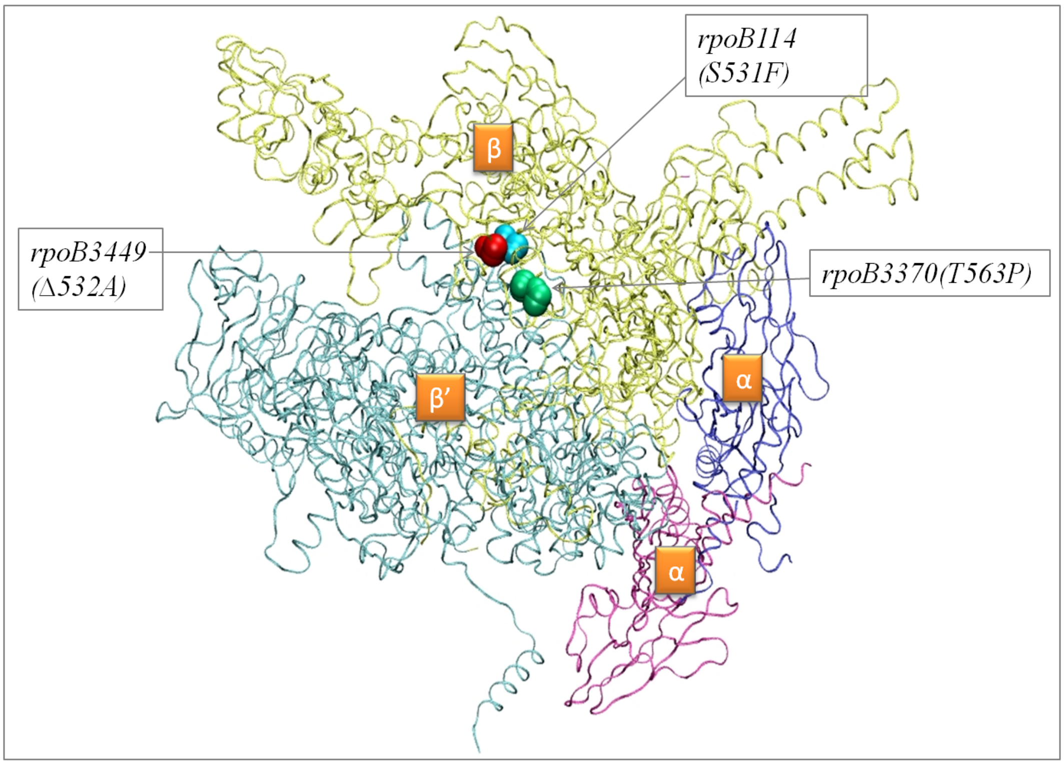

Figure 1.2: *A 3D molecular structure of RNAP core enzyme depicting the three point mutations in the β subunit forming RNAP stringent mutants.*

our study), found that three *rpoB* mutations: *rpoB114*(S531F), *rpoB3449*(Δ532A) and *rpoB3370*(T563P) which confer rifampicin resistance (Rif^T), have in particular reduced

transcription from promoters P1 and P2 of *rpoD* operon [42], which are negatively controlled by stringent response. Furthermore, it was also observed that these stringent polymerases have an altered expression of the genes that are positively controlled by stringent response [43]. This points to the fact that there must be a functional relevance within the region of β subunit (coded by *rpoB* gene) of the RNA Polymerase defined by Rif^{T} mutations. They are an useful tool to investigate further the complexity of the stringent response in *Escherichia coli* at the genetic level and can help us to understand the regulation of stringent promoters in bacterial system.

Analog and Digital Control

The complete genetic system of *E.coli* can be described in terms of Analog and Digital control. *Analog Control* refers to the changes in DNA topology i.e. DNA supercoiling. *Digital Control* refers to the Transcriptional Regulatory Network (TRN) formed by the transcription factors which further leads to the Metabolic Network [44] [45]. Supercoil dynamics of the chromosomal DNA can be considered as an analogous form of information i.e. continuous in its function of regulating expression of specific genes. While, the transcriptional regulatory network provides static information through interaction between specific discontinuous elements (a pair of regulating or regulated genes) [45]. It has been observed that there is a coherent relationship between chromosomal organization and the genetic expression pattern. DNA topology denotes the amount of energy that is present inside the cells which is the utmost requirement for cell growth. During the exponential growth phase in *E.coli*, a more negatively supercoiled DNA is preferred by σ^{70} holoenzyme (Eσ^{70}) whereas more relaxed DNA is preferred by σ^{S} holoenzyme (Eσ^{S}) during the stationary phase of the growth cycle [46]. Same is true for the NAPs which are expressed in different stages of growth as shown in fig.1.3. Consequently, specific genes are transcribed by the RNA Polymerase with the help of σ factors and NAPs, producing specific proteins and metabolic enzymes according to the requirement of the growth phase. Furthermore, energy is produced as a by product of the metabolic pathways in the form of ATP/ADP and ppGpp which can thus be sensed by the DNA topology and then the cycle of genetic regulation (above mentioned) starts again. Hence, a feedback loop is formed between the DNA supercoil dynamics, the transcription machinery and the metabolic network. Therefore, it can be said that 3D spatial arrangement of the chromosome can be considered as the *brain* of the transcriptional regulation, both at the local and global level.

The aim of this study was to determine a structural coupling between *Transcription Machinery* (RNAP + σ factors + NAPs), *DNA Topology* and *Metabolic Energy* in *E.col*i

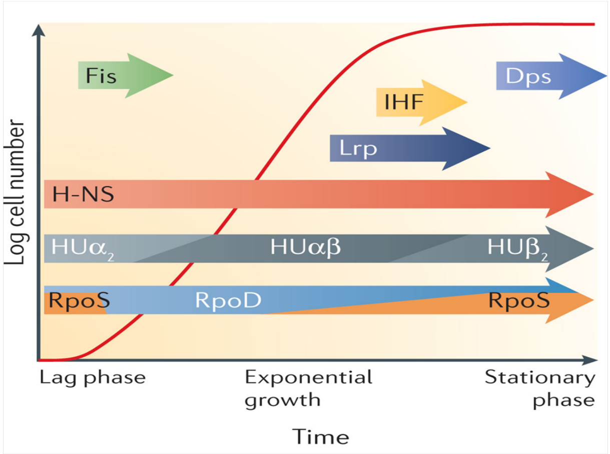

Figure 1.3: *The expression patterns of the major nucleoid-associated proteins and rpoDand rpoS genes which code for σ^{70} and σ^{S} respectively, depicted on the bacterial growth curve along lag, exponential and stationary phase (taken from [47])*

stringent mutants as depicted in fig.1.4. Three RNA polymerase mutants, *rpoB114*(S531F), *rpoB3449*(Δ532A), *rpoB3370*(T563P) and *DksA* mutant were compared to the wild type cells in this study. We expected to see an opposite behaviour genotypically as well as phenotypically because *dksA* mutant lacks DksA protein which is a co–regulator of ppGpp during the stringent response. Therefore, the *dksA* mutant might lack an efficient stringent response in absence of DksA protein and the *rpoB* mutants have been shown to act like stringent mutants even in absence of stringent response.

In order to establish the structural coupling theory, three strategies were employed. (1) The change in the transcription machinery was analysed by determining the RNAP

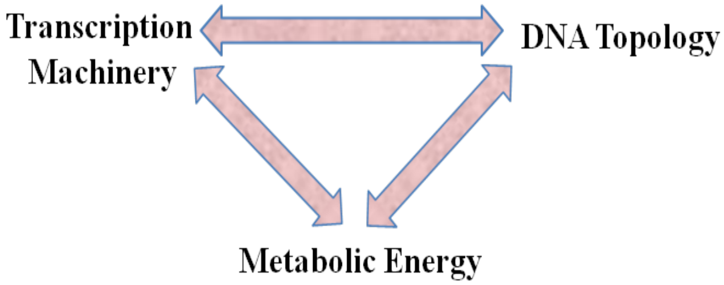

Figure 1.4: *Structural coupling between the transcription machinery formed by RNAP, σ factors and NAPs, the DNA supercoil dynamics and the metabolic energy produced by the central metabolism in Escherichia coli.*

σ factor composition and variation in levels of NAPs during the course of the growth. (2) Changes in DNA topology were examined by looking at the distribution of topoisomers in plasmid (pUC18) transformed into these strains using high resolution gel electrophoresis. Finally, (3) the metabolic energy changes in these strains were determined by investigating the RNA supramolecular composition i.e. all the proteins which bind to RNAP during initiation of transcription.

2 Materials and Methods

2.1 RNAP σ Factor and NAP Composition

2.1.1 Bacterial Strains

The following bacterial strains were used: CF2010 wild type (CF2010 WT), CF9239 **Δ***dksA* :: kan (CF9239 **Δ***dksA*), CF2016 *rpoB114* :: rif (CF2016 *rpoB114*), CF2024 *rpoB3370* :: rif (CF2024 *rpoB3370*), CF2030 *rpoB3449* :: rif (CF2030 *rpoB3449*)

2.1.2 Bacterial Growth

Bacterial strains from the glycerol stocks, were plated on dYT agar plates containing the respective antibiotic. They were grown overnight at 37°C and then used for inoculation of 10 ml dYT media containing the respective antibiotic. After about 12 hours of growth in a shaker at 37°C, respective amounts were used to inoculate 100 ml of dYT in order to get an $\mathbf{O.D_{600}}$ of 0.1. The cultures were grown in the fermenter (Sartorius, Göttingen, Germany) until they reached $\mathbf{O.D_{600}}$ of 0.5 (mid exponential phase), around 2.0 (transition phase) and 4.0 (early stationary phase). The O.D measurements were done every 30 min. 4×50 ml samples were collected and 5 ml of 10× killing buffer (50 mM Tris–HCl pH 7.5, 15 mg/ml sodium azide, 0.6 mg/ml chloramphenicol) was added. The samples were centrifuged at 10,000 rpm for 10 min at 4°C in Beckman Coulter centrifuge, C6050 rotor (Beckman Coulter GmbH, Germany). The pellets were washed twice with Phosphate Buffered Saline (PBS) and then they were stored at -20°C until further use (for SDS–PAGE studies). Also 4×2 ml samples were collected in 2 ml eppendorf tubes and 200 μl 10× killing buffer was added to them followed by centrifugation at 5,000 g at 4°C for 5 min. Supernatant was discarded and pellets were snap frozen in liquid nitrogen and stored at -80°C (for RT–PCR studies). Furthermore, 3×500 ml samples from the rest of the culture were collected and centrifuged for 20 min at 4,200 rpm in a Multifuge–3 S–R Heraeus centrifuge (Schnakenberg Medi-

zin & Labortechnik GmbH, Germany). After discarding the supernatant, pellets were washed twice with PBS and stored at -20°C until further use (for BN–PAGE studies).

2.1.3 Protein Extraction for SDS-PAGE

The above frozen pellets were thawed on ice and resuspended in 1 ml of solubilization buffer slowly, so that no foam is formed and transferred to 2 ml eppendorf tubes.Samples were sonicated (90% power, 5 sec ON/5 sec OFF) for 30 sec and then kept on ice for 30 sec. This procedure was repeated 3 times. Afterwards, they were centrifuged at 13,000 rpm for 30 min at 4°C in a Biofuge fresco Heraus centrifuge (Schnakenberg Medizin & Labortechnik GmbH, Germany). Obtained supernatants were transferred into new tubes and were stored at -20°C until further use.

2.1.4 Protein Concentration Quantification

The protein concentration of the samples was determined using BCA^{TM} Protein Assay Kit (Pierce Chemical Company, Rockford, IL, USA), according to the Microplate Procedure. The microplate reader GENius (Tecan Instruments, Salzburg, Austria) and the Magellan V 6.5 software were used for detection and susequent quantification of the protein concentration in the respective bacterial samples.

2.1.5 SDS-PAGE

For the gel electrophoresis experiments, 8–15% gradient gel for the σ factors and 12–20% gradient gel for the NAPs were prepared. 15 μg of proteins were loaded per gel well. 4× cracking buffer (3 ml of 0.5 M Tris HCl pH 6.8, 3 ml of 10% SDS, 2 ml of 8% Glycerol, 2 ml of β–mercaptoethanol and a tip of bromophenol blue) was added to the samples and respective amounts of ddH_20 to get the same final volume. 10 μl of ColorPlusPrestained Protein Marker, Broad Range (7–175 kDa) (New England BioLabs, Frankfurt, Germany) was used. The gel was run for 20 min at 80 V followed by 1.5 h at 120 V or until running front reached the bottom of the gel.

2.1.6 Western Blotting

Proteins were transferred using Glassycarbon Semidry Transfer System (Schleicher & Schnell, Dassel, Germany) on a Nitrocellulose membrane (Schleicher & Schnell, Dassel, Germany) and the blotting was done for 1.5 h. After the transfer, the membrane was stained with Ponceau S solution to see the presence of protein bands. Then the

membrane was destained with 0.1 M NaOH and washed with Tris-Buffered Saline Tween-20 (TBST). The membrane was then blocked overnight with 5% milk powder in TBST at -4°C on a rocking table. Quantitative Western analysis was performed using the ECF^{TM} kit (GE Healthcare, Munich, Germany) and the primary rabbit monoclonal antibodies raised against H-NS, Fis, Ihf and Dps proteins and primary mouse monoclonal antibodies against β', σ^{70}, σ^{S}, σ^{F} and σ^{E} subunits of the RNAP complex (Neoclone, Madison, USA). All of the antibodies were diluted 1:1000 in 1% milk powder in TBST except for the σ^{70} antibody which was diluted 1:10000. After blocking the membrane, it was incubated in primary antibodies for 2 h at RT on a rocking table. Then the membrane was washed 3×10 min with TBST and incubated with a secondary anti-rabbit (for NAPs) and anti-mouse (for σ factors) IgG AP-linked antibody (Cell Signaling Technology, Inc., Danvers, MA, USA) for 1 h at RT. After the incubation, the membrane was washed 3×7 min with TBST and incubated with ECF (GE Healthcare, Munich, Germany) for 5 min followed by scanning with Fuji Phosphoimager FLA7000 (Fujifilm, Tokyo, Japan). Quantification was done using AIDA 2D densitometry analysis, version 4.0 software.

Western Blot Stripping

In order to detect more than one substrate nitrocellulose membranes were stripped using Roth ®– Free Stripping (Carl Roth GmbH + Co. KG, Karlsruhe, Germany). Membranes were incubated at 56°C for 1 h followed by washing 8×5 min with TBST and then blocking overnight in 5% milk powder in TBST at -4°C.

2.1.7 Phenotypic Assay

Vials containing api®M medium from bioMerieux® (Marcy l'Etoile, France) were boiled in water bath at 95°C for 8 min and then kept in upright position for 1 h to let the medium solidify. Then single colonies of the strains were inoculated followed by incubation at 37°C for 24 h. At the end, pictures were taken and mobilities of the individual strains were assessed.

2.2 DNA Topology

2.2.1 Bacterial Growth for DNA Topology Analysis

Cells transformed with pUC18 were inoculated in 10 ml of dYT medium containing ampicillin and grown overnight at 37°C, 150 rpm. Appropriate volumes of the

overnight culture were inoculated in 200 ml dYT containing ampicillin to get an $\mathbf{O.D_{600}}$ of 0.1 and incubated at 37°C at 150 rpm on a shaker. The growth was monitored by measuring the $\mathbf{O.D_{600}}$ every 30 min. Cells were harvested at different growth phases, exponential, transition, early stationary and late stationary, by centrifuging 50 ml of the culture at 7,000 rpm for 5 min. The harvested cells were frozen and stored for plasmid isolations.

2.2.2 High Resolution Gel Electrophoresis

From the harvested cells, plasmids were isolated in different growth phases using Roti®–Prep Plasmid MINI kit (Carl Roth GmbH + Co. KG, Karlsruhe, Germany). Concentrations of these isolated plasmids were determined using NanoDrop 2000 (Thermo Fisher Scientific Inc, GmbH, Germany). Distribution of the topoisomerases of the isolated plasmids were visualized using high resolution gel electrophoresis. 400 ml of 1% agarose was prepared using 1× TBE buffer containing 1.5 μg/ml chloroquine. The gel was first pre-run at 40V for 30 min at 4°C with 1× TBE buffer also containing 1.5 μg/ml chloroquine. 500 ng of samples were loaded along with Fermentas® Lambda DNA/PstI marker and run at 45V at 4°C for 21 h. The gels were washed with water for 30 min followed by staining with Ethidium Bromide for 2 h and then washed with water again for 2 h. The gels were then visualized using Insta Gel documentation system (BioRad, Munich, Germany). Quantification of the gels was performed using AIDA, version 4.0 software.

2.3 RNAP Supramolecular Composition

2.3.1 Protein Purification through HPLC

The frozen bacterial pellets were thawed on ice, followed by resuspension in 2 ml Sonication/Equilibration buffer (10 mM Tris-HCl pH 8.0, 10 mM MgCl2, 0.1 mM EDTA, 0.1 mM DTT, 5% (v/v) Glycerin, 230 mM NaCl). 20 μl of 100× stock Protease Inhibitor Mix HP PLUS (SERVA Electrophoresis, Heidelberg, Germany), 70 U/ml Benzonase (Sigma-Aldrich Chemie GmbH, Steinheim, Germany) and 2 g of 0.25–0.50 mm glass beads (Carl Roth GmbH, Karlsruhe, Germany) were added to the above solution. Cracking of cells was brought about by 30 sec sonication (90% power, 5 sec ON/5 sec OFF), 1 min on ice and 30 sec vortexing. This cycle was repeated 5 times. Afterwards, incubation on ice for 1 h was done and supernatants were transferred to 2 ml eppendorf tubes. They were centrifuged at 13,000 rpm for 30 min at 4°C in

a Biofuge fresco Heraus centrifuge (Schnakenberg Medizin & Labortechnik GmbH, Germany). They were further centrifuged for 10–15 min to remove the cell debris completely. These extracts were passed through an affinity column HiTrap Heparin 1 ml (GE Healthcare, Munich, Germany) using an ÄKTA^{TM} system (GE Healthcare, Munich, Germany). By increasing the NaCl concentration in the Equilibration buffer, from 230 mM to 1 M, at a flow rate of 0.5 ml/min, the RNAP complex was eluted using gradient elution. 1 ml fractions of the proteins were collected. Fractions A8, A9 and A10 were pulled together and concentrated on Amicon® Ultra Centrifugation Filters with a 3 kDa molecular weight cut-off filter (MWCO) (Millipore Corporation, Billerica, MA, USA), by centrifugation at 3,990 g in a swing-rotor centrifuge. To concentrate the samples, Equilibration buffer containing no NaCl was used, to reach the final salt concentration of < 50 mM, which was a requirement for Blue-Native gel electrophoresis studies. Protein concentrations were determined using BCA^{TM} Protein Assay Kit described before.

2.3.2 Blue Native Polyacrylamide Gel Electrophoresis (BN-PAGE)

The BN-PAGE experiment was performed according to [48]. 32:1 ratio of the acrylamide-bisacrylamide was made according to Recipe 8 from the paper. 20 ml of 4%-15% seperating gel was prepared. 50 μg of the concentrated and desalted protein samples were prepared and respective volumes of 4$\times$ native loading buffer (Invitrogen, Life Technologies GmbH, Frankfurt, Germany) were added to each sample and loaded in triplicates on the gel. 15 μl of the unstained native protein marker from Invitrogen was loaded as a standard. The gel was run at 150 V for 8 h at RT. The marker lane was cut out, destained shortly in coommassie destaining solution (ethanol 10% (v/v), orthophosphoric acid 2% (v/v)) [49] rinsed with water shortly and put back on the glass slide next to the gel at approximately matching position. The RNAP band was cut out from this gel by looking at the marker lane at 460 kDa size. The these bands were incubated in sample buffer (Recipe 16: [48]) for 10 min and boiled for 20 sec in the microwave, and then kept at RT for 15-20 min. 8-20% SDS-PAGE gel was prepared with a 3.5% stacking gel and these bands were stacked in each well. After this step, 0.5% low melting agarose (Biozym, Hessisch Oldendorf, Germany) in SDS-PAGE running buffer was prepared and put on top of these gel pieces as well as in empty wells. 15 μl of ColorPlus Prestained Protein Marker, Broad Range (7-175 kDa) (New England Biolabs GmbH, Frankfurt, Germany) was put in the centre of small pieces of whatman paper and stacked in the wells on either side of these gel pieces. After a few minutes

till the agarose solidified, the gel was run at 150 V for 15 h or until the running front reached the bottom of the gel. The gel was then first washed thrice for 15 min in ddH_2O followed by staining in coomassie staining solution (CBB G-250 0.2% (w/v), aluminum sulfate 14-18 hydrate 5% (w/v), ethanol 10% (v/v), orthophosphoric acid 2% (v/v)) O/N and then destained for 2 h the next day [49]. Subsequently, the gel was put in ddH_2O for intensifying the color of the bands. The gels were kept in 5% acetic acid solution at 4°C for storage.

2.3.3 MALDI-TOF

The protein bands observed in the BN-PAGE gels, which are assumed to be a part of the RNAP complex, were cut out and processd for MALDI-TOF detection using [50] protocol: In-gel digestion for mass spectrometric characterization of proteins and proteomes. The gel pieces were put in 500 μl acetonitrile for 10 min and spinned down after which the liquid was discarded. Pieces were put in 30-50 μl of DTT solution (10 mM DTT in 100 mM ammonium bicarbonate) and incubated at 56°C for 30 min. 500 μl acetonitrile was added to the tubes, followed by 10 min incubation. Then 30-50 μl of iodoacetamide solution (55 mM iodoacetamide in 10 mM ammonium bicarbonate) was added followed by incubation at RT in a dark place for 20 min. Gel pieces were then shrinked by adding acetonitrile and then destained by adding 100 μl of 100 mM ammonium bicarbonate/acetonitrile (1:1 v/v). They were incubated for 30 min with occasional vortexing. Again 500 μl of acetonitrile was added and pieces were incubated for some minutes and vortexed. Now the pieces were ready for in-gel digestion. The in-gel digestion of the samples was performed using Protease MAXTM Surfactant Trypsin Enhancer in gel digestion protocol. Afterwards, 1 μl of each sample was spotted on an AnchorChip PAC 384 HCCA plate (Bruker Daltonics, Bellirica, MA, USA), left for drying for 3 min and then washed with 25 μl 0.1% TFA in 10 mM ammonium dihydrogen phosphate by slowly pippeting in an up and down motion. Now the samples were ready for MALDI MS analysis using the Bruker Daltonics Autoflex II TOF/TOF instrument (Bruker Daltonics, Bellirica, MA, USA), as described in Yu-Xuan and Wei-Hua, 2010. External calibration was done using Bruker peptide calibration standards pre-spotted on the plate. The mass spectra (MH+) were detected by Flex Control (version 3.0, Bruker Daltonics) recording in the range of 500 – 4,000 Da. After the MS were detected in LIFT (Laser-induced forward transfer) mode, they were combined using the BioTools software (version 3.1, Bruker Daltonics). Furthermore, they were searched against theoretical digests using the MASCOT software (Matrix

Science). For our experiments, the search parameters were set as $\pm$ 50 ppm for the PMF peptide tolerance and $\pm$ 0.5 Da for the MS/MS tolerance. Search results that were statistically significant ($p < 0.05$) were taken into account. In some cases the error range was put to the highest after 50 ppm to get a significant result with a score above 60. The BN–PAGE and MALDI techniques are detailed in [51].

3 Results and Discussion

3.1 Expression of σ Factors and NAPs in *DksA* and Stringent Mutants

Wild type, **Δ***dksA* and *rpoB* mutant strains of *E.coli* were grown under same conditions until they reached $\mathbf{O.D_{600}}$ of 0.5 (mid exponential phase), $\mathbf{O.D_{600}}$ of around 2.0 (transition phase) and $\mathbf{O.D_{600}}$ of around 4.0 (early stationary phase) and then pellets were collected for these three different phases. The growth of the two biological replicates was recorded after every 30 min and growth curves were plotted. 15 μg crude protein extract of each sample was loaded onto an SDS gel and transferred onto a nitrocellulose membrane by western blotting technique. Presence of σ factors and NAPs was determined using enhanced chemifluorescence (ECF).

Western blot analysis and the relative concentration plot of the RNAP subunits (fig.3.1 and fig.3.2) depict the amounts of σ^{70} and σ^{S} in the whole cell extract in comparison to their amounts bound to the RNAP enzyme during the course of the growth of these strains. For the exponential phase data of σ factors, strains were grown in flasks until they reached an $\mathbf{O.D_{600}}$ of 0.5 and for the transition and stationary phase, strains were grown in a fermenter until they reached an $\mathbf{O.D_{600}}$ of 2.0 and 4.0 respectively. σ^{S} and other alternative σ factors could not be detected in fermenter grown samples and therefore, data was analysed from the flask grown samples for the same. The results show that σ^{70} is present in higher amounts in the crude cellular extracts and very little is bound to the RNAP. However, in the transition phase, all σ^{70} appears to be associated with the RNAP and very little is left inside the cells across all strains. Further towards stationary phase, σ^{70} seems to be released from the polymerase as its amount increases in the whole cell extract and becomes negligible in terms of associated with the polymerase. So concentration of σ^{70} remains more or less constant throughout the growth cycle but is much more uniform in the *rpoB* mutants. When we look at the σ^{S} levels, none of it is bound to the polymerase and all σ^{S} seems

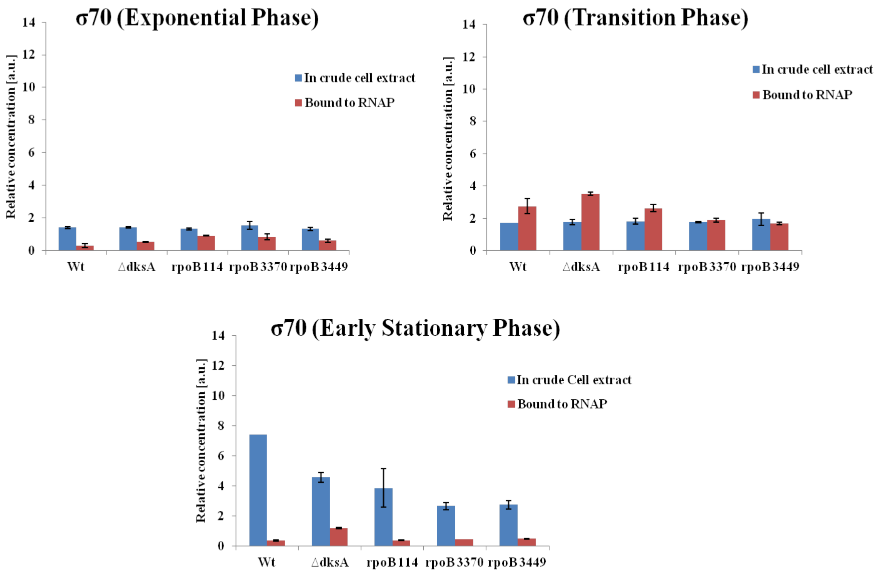

Figure 3.1: *Quantification of the σ^{70} normalized to β' values in crude protein extracts obtained from CF2010 wild type, CF9239 ΔdksA, CF2016 rpoB114, CF2024 rpoB3370 and CF2030 rpoB3449 in exponential, transition and early stationary growth phases using western blot analysis. Bars indicate the standard error.*

to be available in the crude cell extract. However, in the transition phase, the amount of σ^S that is available in the crude cell extract gets associated with the RNAP in the wild type and *rpoB114* mutant. However, in the *dksA* and *rpoB3449* mutant, it was observed that very low concentrations of σ^S is present in the crude cellular extract and therefore lower concentrations bound to the polymerase. In *rpoB3370* however, higher concentration of σ^S seen in the crude extract whereas drastically lower in association with RNAP. Moreover, in the early stationary phase, it was observed that relatively, the amounts of σ^S are more in the crude cellular extracts and less in the context of bound to the RNAP in all the strains. Concentration of σ^S remains relatively constant from transition to stationary phase in Δ*dksA* and *rpoB3449* strains. However, in wild type and *rpoB114*, more σ^S is seen in the crude cell extract than in association with RNAP and *rpoB3370* shows lower amounts in both. Analysing fig.3.1 and fig.3.2, it can be deduced that σ^{70} is produced in fairly good amounts during the exponential phase of growth in the wild type, Δ*dksA* and *rpoB* mutants but this σ^{70} does not bind to the RNAP fully. Why such huge amounts of σ^{70} is produced by the bacterial system is still unclear. On the contrary, σ^S is produced in the exponential phase and also

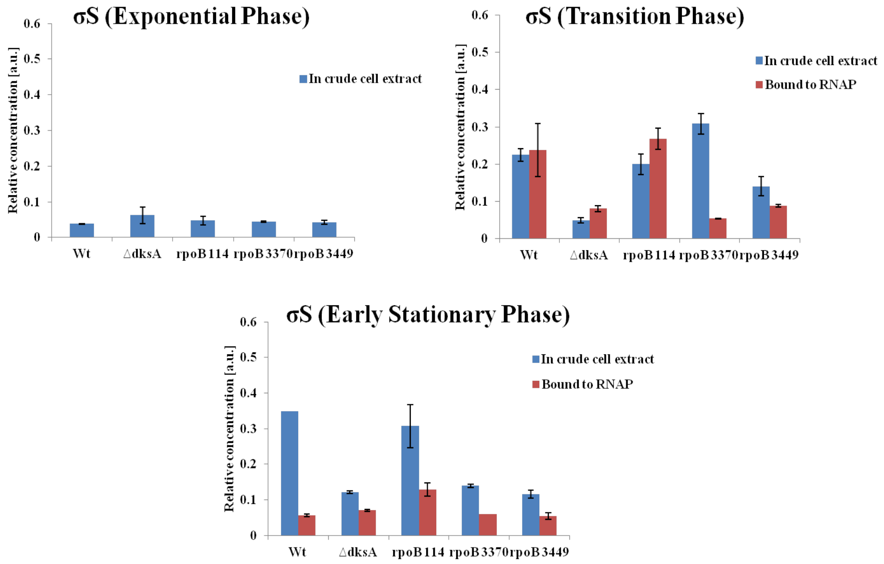

Figure 3.2: *Quantification of the σ^S normalized to β' values in crude protein extracts obtained from CF2010 wild type, CF9239 ΔdksA, CF2016 rpoB114, CF2024 rpoB3370 and CF2030 rpoB3449 in exponential, transition and early stationary growth phases using western blot analysis. Bars indicate the standard error.*

in the transition phase and gets immediately bound to the RNAP. These two opposite observations suggest that there must exist a competition between these two σ factors in the transition phase. At that particular time of growth, the cell has to decide whether to continue the exponential way of growth or to go into the stationary phase or the resting phase. Then at this moment, the RNAP has to decide whether to keep σ^{70} bound to itself and release σ^S or to in contrast, release σ^{70} and keep σ^S in association with it. This decision making process is a crucial step during the bacterial growth cycle as both these σ factors are required for transcription of functionally different set of genes and prefer distinct organization of the chromosome.

When alternative σ factors were investigated in these strains, rather peculiar results arose. Wild type, Δ*dksA* and *rpoB* mutants were grown in flasks under similar conditions until they reached an **O.D**$_{600}$ of 0.5 which is assumed to be mid-exponential growth phase. σ^E shows an increase in the *dksA* mutant and a decrease in the *rpoB* mutant *E.coli* cells (fig.3.3). Moreover, same pattern is observed in case of σ^F factor as well (fig.3.4). To further confirm a low quantity of σ^F factor (responsible for the flagellar movement of *E.coli* cells) in the *rpoB* mutants and a rather high quantity in the

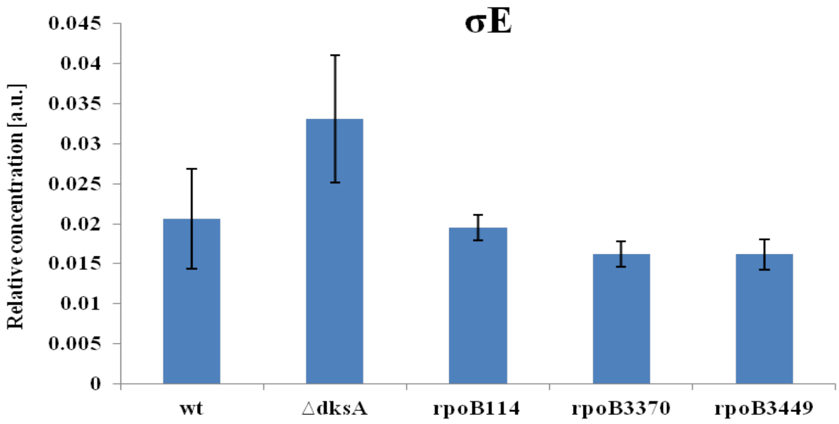

Figure 3.3: *Quantification of the σ^E normalized to β' values in crude protein extracts obtained from CF2010 wild type, CF9239 ΔdksA, CF2016 rpoB114, CF2024 rpoB3370 and CF2030 rpoB3449 in exponential growth phase using western blot analysis. Bars indicate the standard error.*

dksA mutant (from blot analysis), a phenotypic swarming assay was performed and the results showed that the motility of CF2016 *rpoB114*, CF2024 *rpoB3370* and CF2030 *rpoB3449* cells does reduce dramatically and the motility of the *dksA* mutant increases when compared to the wild type strain (fig.3.5). Above observations lead to the conclusion that all σ factors work in a unified manner. If one of the two major σ factors (σ^{70} or σ^S) get reduced, then the system up-regulates the production of other alternative σ factors to compensate the absence of the most important ones. From fig.3.2, we can see that σ^S reduces in the *dksA* mutant and concentrations of alternative σ factors like σ^E and σ^F increase drastically in the same. Whereas, in the *rpoB* mutants, σ^{70} and σ^S more or less remains the same throughout the growth cycle but then σ^E and σ^F reduce significantly in these mutant strains when compared to the wild type in which amounts of all sigma factors remain relatively constant. Hence, this proves the fact that σ factor composition of RNAP varies considerably between different backgrounds during the growth of *E.coli* bacteria and proportion of σ factors is different in different strains.

Changes in NAP Composition

Fig.3.6 shows that the amount of H-NS protein increases radically in the *rpoB* mutants whereas remains almost the same in the *dksA* mutant when compared to the wild type in the early stationary phase. Whereas, Fis protein levels increase in Δ*dksA* and

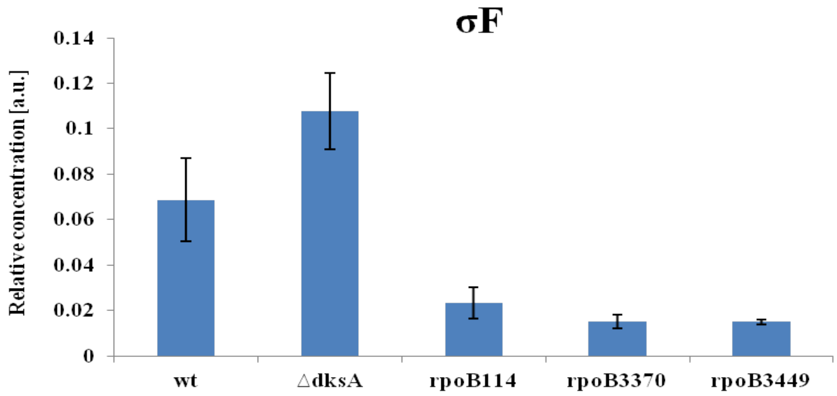

Figure 3.4: *Quantification of the σ^F normalized to β' values in crude protein extracts obtained from CF2010 wild type, CF9239 ΔdksA, CF2016 rpoB114, CF2024 rpoB3370 and CF2030 rpoB3449 in exponential growth phase using western blot analysis. Bars indicate the standard error.*

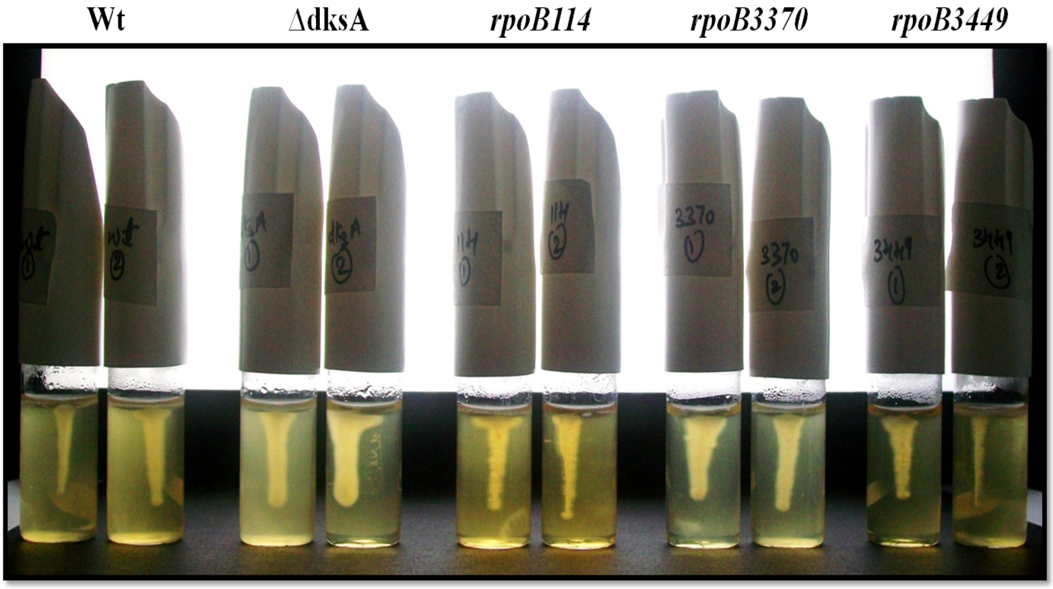

Figure 3.5: *Swarming/Phenotypic assay of two biological replicates of CF2010 wild type, CF9239 ΔdksA, CF2016 rpoB114, CF2024 rpoB3370 and CF2030 rpoB3449 strains depicting the motility differences.*

rpoB3449 mutant in comparison to the wild type during the transition phase and remains relatively same in the exponential and early stationary phase in all the strains. However, Dps protein increases considerably in the *rpoB* mutants as compared to the wild type and *dksA* mutant in the early stationary phase. Moreover, IHF protein as well shows increment in the *rpoB* mutants in the early stationary phase while decrease in *dksA* mutant and vice-versa in the transition phase.

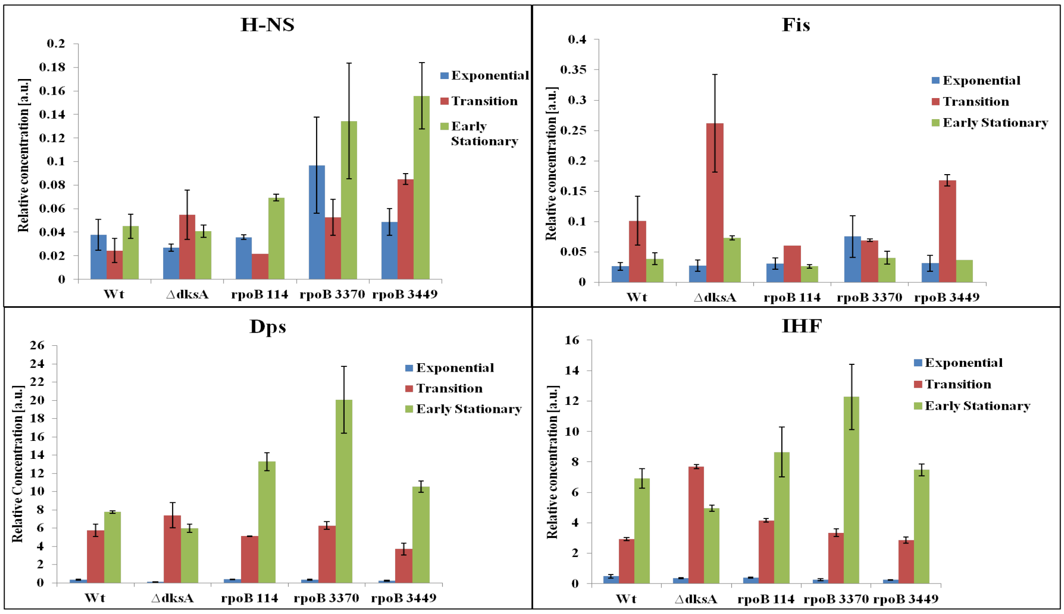

Figure 3.6: *Quantification of the NAPs (Fis, H-NS, Dps and IHF) normalized to β' values in crude protein extracts obtained from CF2010 wild type, CF9239 ΔdksA, CF2016 rpoB114, CF2024 rpoB3370 and CF2030 rpoB3449 in exponential, transition and early stationary growth phases using western blot analysis. Bars indicate the standard error.*

Nucleoid associated proteins also show a difference in their amount patterns in the mutant strains when we compare their amounts to the wild type strain. All mutants i.e. Δ*dksA* and *rpoB114*, *rpoB3370* and *rpoB3449*, produce more NAPs in contrast to the wild type. The only difference is seen in case of Fis protein which is produced more in Δ*dksA* than the *rpoB* mutants as well as the wild type as the Fis protein is expressed mostly during the exponential phase and *rpoB* mutants have been known to have enhanced stationary phase even in absence of it. In conclusion, there are more radical changes in the NAP composition in the mutants in contrast to the wild type cells.

3.2 Changes in DNA Topology

For demonstrating the changes in DNA supercoiling in Δ*dksA* and *rpoB* mutant strains in comparison to the wild type cells, high resolution gel electrophoresis was performed. This high resolution gel resolves the different populations of topoisomers of the plasmid. Consequently, we analysed these bands by AIDA (1D-densitometry) software, to see how topoisomer distribution changes along the growth of these strains. PUC18 plasmid which was transformed into the wild type and mutant cells and further isolated, as described in the methods section. This procedure was done for four different phases of growth: exponential, transition, early stationary and late stationary and all mutant cells were analysed against the wild type *E.coli* cells. When we

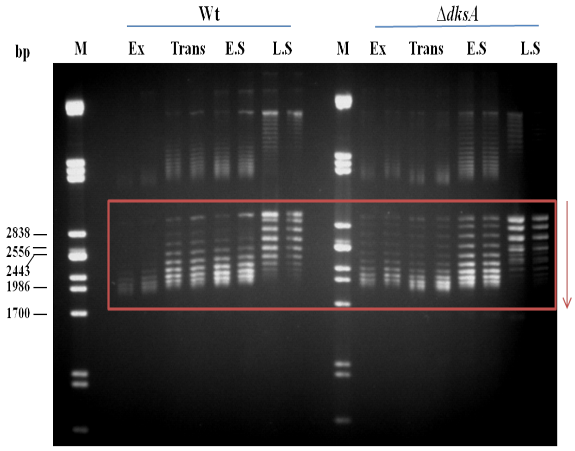

Figure 3.7: *High resolution gel electrophoresis indicating topoisomer distribution in wild type and ΔdksA strain. Topoisomers of plasmid pUC18 were isolated from two biological replicates of CF2010 wild type and CF9239 ΔdksA at exponential (Ex), transition (Trans), early stationary (E.S) and late stationary (L.S) growth phases. Topoisomers (red square) were separated on agarose gel containing 1.5μg/ml chloroquine. At this concentration of chloroquine, more negatively supercoiled topoisomers migrate faster in the gel than the relaxed topoisomers as indicated by the arrow. M–λDNA/PstI marker*

compare the topoisomers of pUC18 plasmid isolated from *dksA* mutant and the wild type strain (fig.3.8), it can be clearly seen that the wild type *E.coli* cells show a gradual change in the DNA supercoiling from being highly supercoiled to getting relaxed while going from exponential to the late stationary phase. Whereas, the *dksA* mutant displays a much stronger change in topology from being more supercoiled in the exponential, transition and early stationary phase and then suddenly getting relaxed in the late stationary phase. This shows the stability and robustness of the system in the wild type *E.coli* cells and a hypersensitive behaviour in the *dksA* mutant. However,

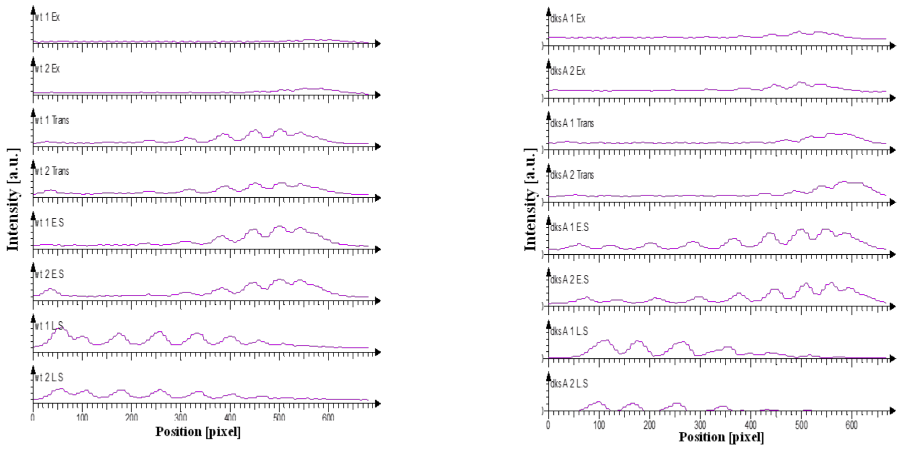

Figure 3.8: *Analysis of the gel.Topoisomers on the gel were analysed using AIDA 1D-densitometry analysis indicating the DNA supercoiling from more negatively supercoiled DNA towards the right and more relaxed DNA towards the left of the graph.*

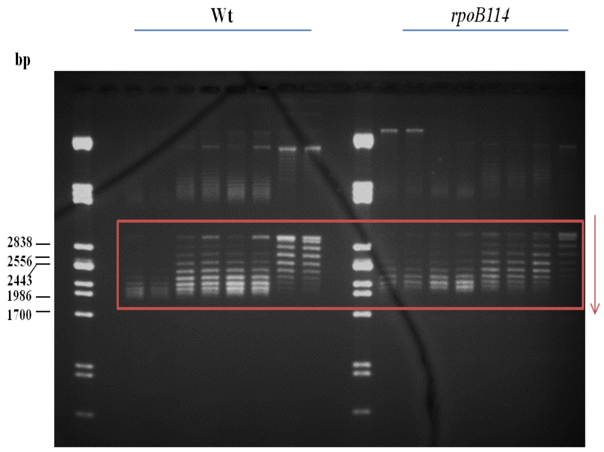

Figure 3.9: *High resolution gel electrophoresis indicating topoisomer distribution in wild type and rpoB114 mutant. Topoisomers of plasmid pUC18 were isolated from two biological replicates of CF2010 wild type and CF2016 rpoB114 at exponential (Ex), transition (Trans), early stationary (E.S) and late stationary (L.S) growth phases. Topoisomers (red square) were separated on agarose gel containing 1.5μg/ml chloroquine. At this concentration of chloroquine, more negatively supercoiled topoisomers migrate faster in the gel than the relaxed topoisomers as indicated by the arrow. M–λDNA/PstI marker.*

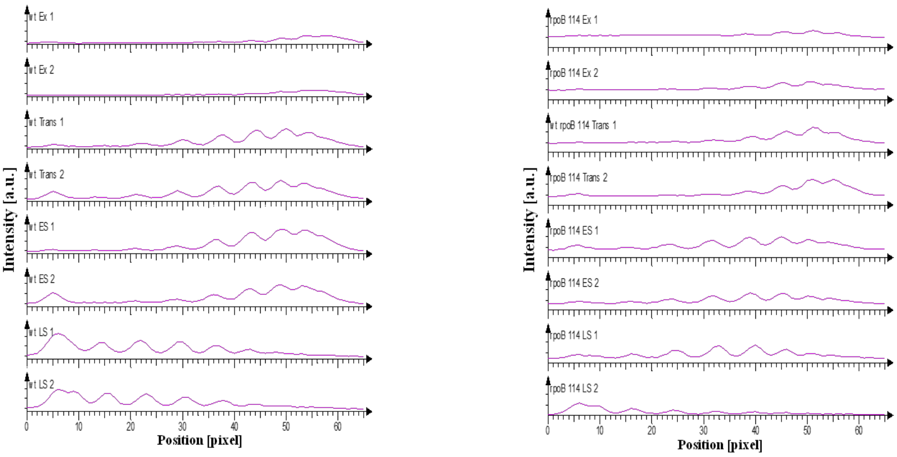

Figure 3.10: *Analysis of the gel.Topoisomers on the gel were analysed using AIDA 1D-densitometry analysis indicating the DNA supercoiling from more negatively supercoiled DNA towards the right and more relaxed DNA towards the left of the graph.*

fig.3.10 shows the topoisomer distribution in the *rpoB114* mutant cells wherein the DNA topology seems to again lack the gradual change as in the wild type. They show more supercoiling in the exponential and transition phase while an early relaxation in the early stationary phase. Nonetheless, they do not relax completely in the late stationary phase as can be seen in the wild type cells. Fig.3.12 and fig.3.14 depict the changes in the DNA topology of *rpoB3370* and *rpoB3449* respectively. In both of these mutants, DNA supercoiling does not change much over the course of the growth cycle. DNA remains supercoiled till the late stationary phase when it relaxes a bit. So, the DNA topology of *rpoB3370* and *rpoB3449* becomes different than **Δ***dksA* and *rpoB114* mutants in the late stationary phase. From the DNA topology analysis of the mutant strains when compared to the wild type, it becomes apparent that variation in the DNA topology becomes less pronounced in the mutants than the wild type along the growth cycle. The gradual change in the topology is lost in the mutants as the cells try to cope up with environmental stresses and all kinds of other stresses, induced due to the mutations in these strains. While the wild type cells show two stages of relaxation in the early and late stationary stages of growth, the *dksA* and *rpoB* mutant *E.coli* cells show less relaxation in these stages. Furthermore, with western blot analysis it was observed that *rpoB* mutants show an increase in the H-NS and IHF proteins (fig.3.6) in the crude cell extracts during the early stationary phase of growth. This finding can be correlated with the supercoiled DNA or less relaxed DNA seen in the early

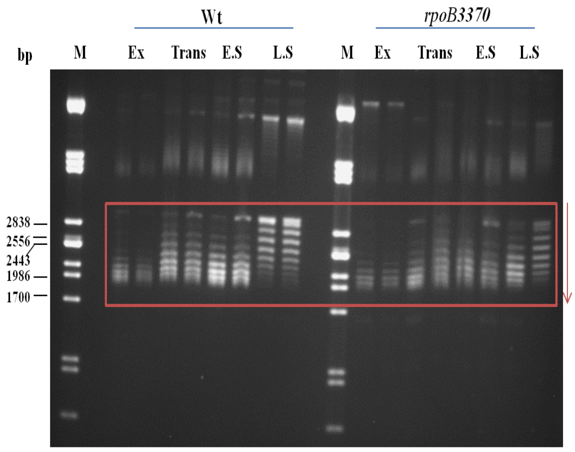

Figure 3.11: *High resolution gel electrophoresis indicating topoisomer distribution in wild type and rpoB3370 mutant.Topoisomers of plasmid pUC18 were isolated from two biological replicates of CF2010 wild type and CF2024 rpoB3370 at exponential (Ex), transition (Trans), early stationary (E.S) and late stationary (L.S) growth phases. Topoisomers (red square) were separated on agarose gel containing 1.5µg/ml chloroquine. At this concentration of chloroquine, more negatively supercoiled topoisomers migrate faster in the gel than the relaxed topoisomers as indicated by the arrow. M–λDNA/PstI marker.*

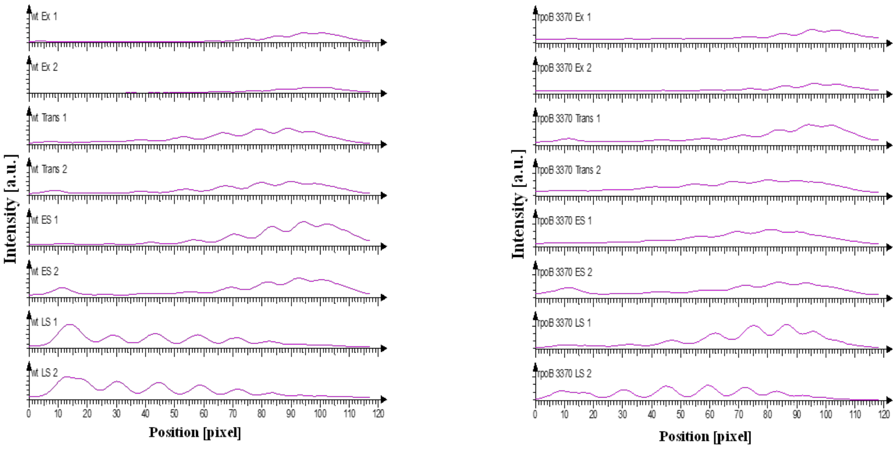

Figure 3.12: *Analysis of the gel.Topoisomers on the gel were analysed using AIDA 1D-densitometry analysis indicating the DNA supercoiling from more negatively supercoiled DNA towards the right and more relaxed DNA towards the left of the graph.*

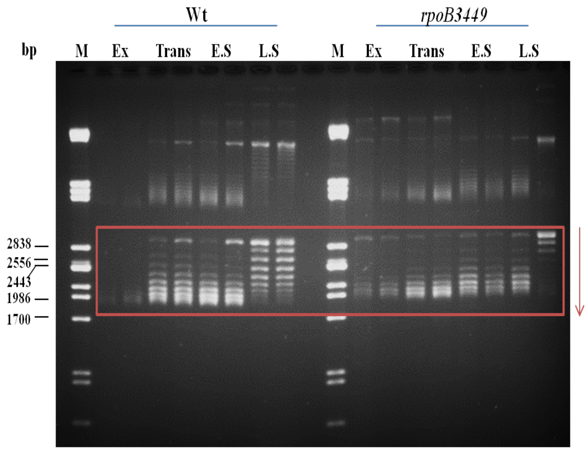

Figure 3.13: *High resolution gel electrophoresis indicating topoisomer distribution in wild type and rpoB3449 mutant.Topoisomers of plasmid pUC18 were isolated from two biological replicates of CF2010 wild type and CF2030 rpoB3449 at exponential (Ex), transition (Trans), early stationary (E.S) and late stationary (L.S) growth phases. Topoisomers (red square) were separated on agarose gel containing 1.5μg/ml chloroquine. At this concentration of chloroquine, more negatively supercoiled topoisomers migrate faster in the gel than the relaxed topoisomers as indicated by the arrow. M–λDNA/PstI marker.*

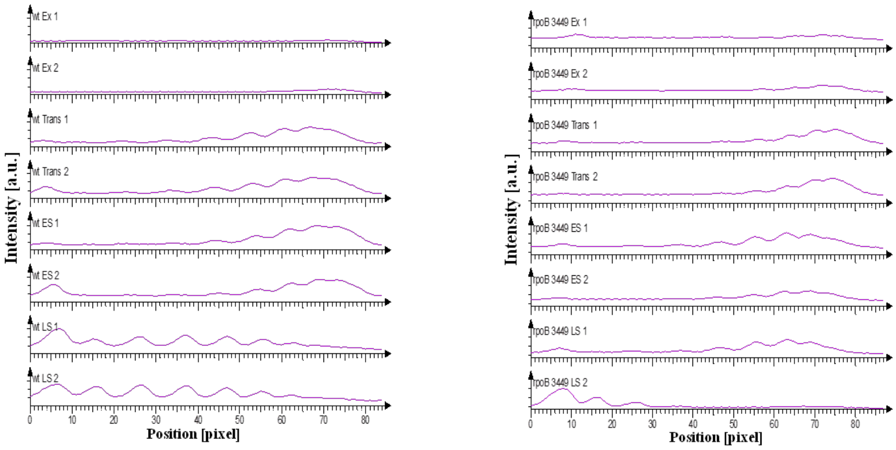

Figure 3.14: *Analysis of the gel.Topoisomers on the gel were analysed using AIDA 1D-densitometry analysis indicating the DNA supercoiling from more negatively supercoiled DNA towards the right and more relaxed DNA towards the left of the graph.*

and late stationary phases as these NAPs directly modulate DNA topology in *E.coli* by bringing about changes in the shape and organization of the DNA [52].

3.3 Changes in RNAP Supramolecular Composition

To find out the components of the complex that RNAP forms during transcription initiation in **Δ***dksA* and *rpoB* mutants of *E.coli*, BN-PAGE was demonstrated to separate the RNAP complex into the respective proteins bound to the RNAP on a gel followed by MALDI-TOF to detect these proteins. 50 μg of the proteins was loaded onto the gel. Some very interesting results came out of this experimental set-up. In the ex-

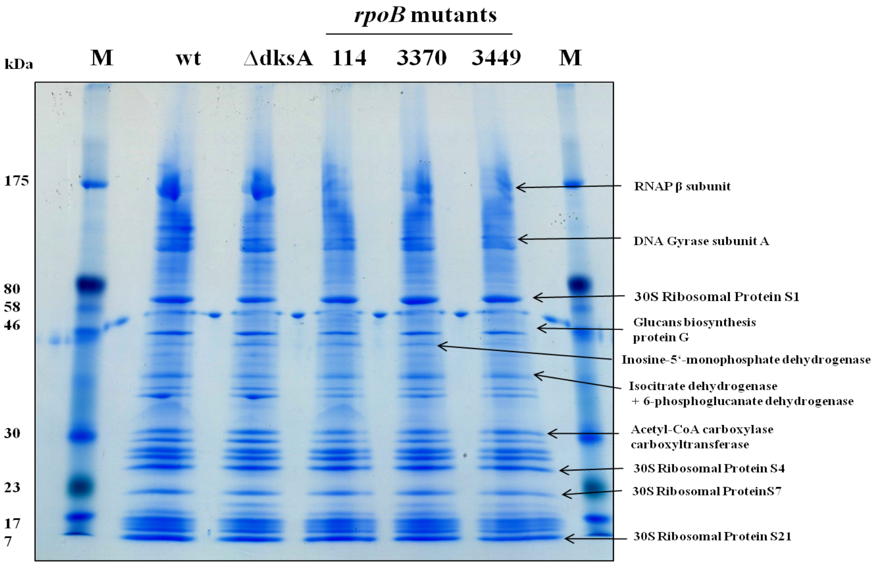

Figure 3.15: *2nd dimension SDS–PAGE gel (8-12%) of CF2010 wild type, CF9239* **Δ***dksA, CF2016 rpoB114, CF2024 rpoB3370 and CF2030 rpoB3449 RNAP complex (from 1st dimension BN–PAGE) in exponential growth phases. M–Prestained Protein Marker. Presence of various ribosomal proteins, metabolic enzymes, RNAP* β *subunit and DNA Gyrase subunit A is indicated by arrows.*

ponential phase, an enrichment of several metabolic enzymes and ribosomal proteins were observed and later on in the transition as well as early stationary phase, this enrichment slowly faded and presence of ribosomal proteins became very little. The most interesting observation was the presence of four enzymes bound to the RNAP complex (fig.3.15 and fig.3.16). These have been known to be involved in different metabolic pathways like biosynthesis of memebrane–derived oligosaccharides, *de novo*

guanine nucleotide biosynthesis, Citric Acid Cycle, Pentose Phosphate Pathway and Lipid Metabolism in *E.coli* bacteria and hence the RNAP complex has been named RNAP supramolecular complex [53].

The most enriched enzyme that is observed is Glucans Biosynthesis Protein G (MdoG) which is present in all the strains throughout the growth cycle. It appears to be getting reduced when the cells reach stationary phase of growth. MdoG has been shown to be very important for synthesis of a class of glucans called membrane-derived oligosaccharides (MDO) [54]. These oligosaccharides are found in the periplasmic space of *E.coli* gram negative bacteria. Moreover, it has also been found that MdoG has similarity to pathogenesis-associated protein of *Pseudomonas syringae pv. syringae* [54] as well as to pathogenesis-associated OpgG protein of *Erwinia chrysanthemi* [55]. Although its involvement in the biosynthesis of MDO is known, its exact function is still in question.

Another important finding of this study was the presence of Inosine $5'$–monophosphate dehydrogenase (IMPDH) which seems to be more expressed in Δ*dksA*, *rpoB114* and *rpoB3370* mutants as compared to the wild type strain. However, further into the growth in transition and early stationary phase, they tend to almost disappear from the RNAP complex. IMPDH catalyzes conversion of IMP to XMP which is the first rate-limiting step in guanine nucleotide biosynthesis and then further gets converted to GMP by the action of GMP synthetase [56]. This IMPDH pathway is found in all organisms. Synthesis of guanine nucleotides is extremely important in any organism as they act as the precursors for RNA and DNA and source of energy for the translation process [57]. Additionally, a mixture of two enzymes: Isocitrate dehydrogenase (IDH) and 6–phosphogluconate dehydrogenase, was found in higher quantities in the wild type, *rpoB3370* and *rpoB3449* mutants and in lesser amounts in Δ*dksA* and *rpoB114* mutants in the exponential phase. But these two enzymes seem to become very little in these strains in the transition and stationary phase. Isocitrate dehydrogenase is a key enzyme in the Tricarboxylic Acid Cycle (TCA cycle). It catalyzes the oxidative decarboxylation of isocitrate producing α–ketoglutarate and CO_2. The first bacterial enzyme shown to be regulated by phosphorylation or dephosphorylation is IDH [58]. *E.coli* is able to make quick shifts between TCA and Glyoxylate bypass pathway with the help of IDH [59]. On the other hand, 6–phosphogluconate dehydrogenase is involved in the production of ribulose 5–phosphate which is a part of Pentose Phosphate Pathway as the major generator of cellular NADPH and also functions in nucleotide synthesis [60]. 6–Phosphogluconate dehydrogenase has an important function of protecting the cells against oxidative damage because of its function of generating NADPH.

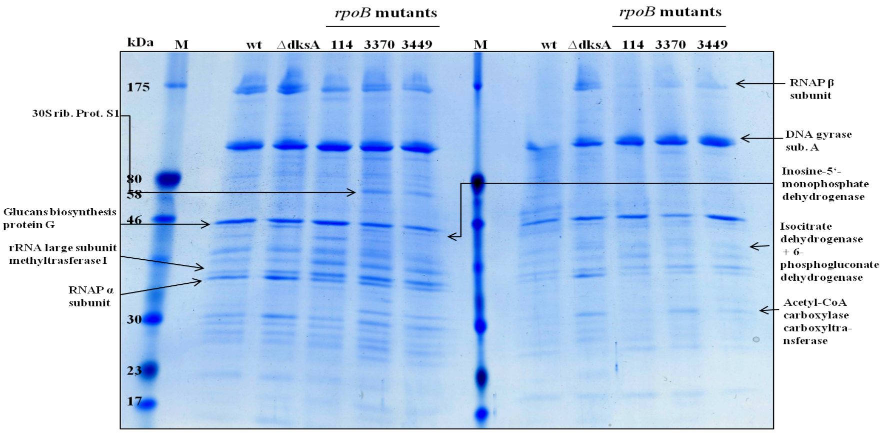

Figure 3.16: *2nd dimension SDS–PAGE gel (8-12%) of CF2010 wild type, CF9239* **Δ***dksA, CF2016 rpoB114, CF2024 rpoB3370 and CF2030 rpoB3449 RNAP complex (from 1st dimension BN–PAGE) in transition and early stationary growth phases. M–Prestained Protein Marker. Presence of 30S ribosomal protein S1, metabolic enzymes, RNAP* **β** *and* **α** *subunit and DNA Gyrase subunit A is indicated by arrows.*

Acetyl–CoA carboxylase carboxyltransferase is seen to be in higher amounts in the wild type and *dksA* mutant in the exponential phase as compared to the *rpoB* mutants and then appears to be more only in the *dksA* mutant than wild type and *rpoB* mutants. This is in agreement with previous observation that carboxyltransferase component of acetyl–CoA carboxylase in *E.coli* is inhibited by ppGpp and pppGpp [61]. *DksA* mutant is assumed to have an impaired stringent response due to absence of DksA protein which is a co-regulator of ppGpp to initiate the stringent response in *E.coli*. Fatty acid synthesis is also reduced during amino acid starvation in stringent response by inhibiting carboxyltransferase by ppGpp and pppGpp. Acetyl–CoA carboxylase catalyzes a very important first committed step in synthesis of long chain fatty acids in the lipid metabolism pathway. In *E.coli*, this enzyme consists of three components: a carboxyltransferase protein, a biotin carboxylase protein and a biotin carboxyl carrier protein [62]. Carboxyltransferase catalyzes carboxylation of acetyl–CoA to form malonyl–CoA. It comprises of a Zinc binding domain, suggesting it has the ability to bind to DNA [63]. This may be the reason why its presence is seen in the RNAP complex during bacterial growth.

Finally, an important finding is the presence of 30S ribosomal proteins (S4, S7 and S21) in all the strains during exponential phase followed by their disappearance in the transition and stationary phases of growth. This presence of ribosomal proteins could

be an artefact of this procedure as transcription and translation processes occur in very close proximity to each other in *E.coli* bacteria. However, another 30S ribosomal protein, S1 was detected which showed some variation in its quantity across these strains. It seems to be present in almost equal amounts in all the strains in the exponential phase but during the transition phase, it appears to be present only in *rpoB3370* and *rpoB3449* and then none of it is there in the early stationary phase. 30S ribosomal protein S1 is a component of the ribosome and has a molecular size of 61 kDa. It has many properties like binding to the $5'$-termini of mRNAs with very high affinity, and its interaction with RNAP [64]. Its ability to interact with RNAP proposes it as a link between transcription and translation. It has been found that S1 protein enhances transcription cycling by binding to the RNAP as well as to the RNA transcript at the same time followed by transcript release from RNAP, thereby allowing the core RNAP to continue the process of re–initiation of transcription [64]. This also suggests that S1 acts at the termination stage of transcription and then continues to function in the translation process.

4 Conclusion and Outlook

The aim of this study was to find a structural and functional coupling between three parameters: the transcription machinery, DNA topology and the metabolic energy in CF2010 wild type, CF9239 Δ*dksA*, CF2016 *rpoB114*, CF2024 *rpoB3370* and CF2030 *rpoB3449* during their growth cycle. This aim was achieved to a large extent as we see some motivating results from the experiments. Firstly, composition of the transcription machinery was studied and changes were analysed. Modulation in σ factor composition in the mutants in contrast to the wild type strain could be clearly observed across the growth cycle. σ^{70} seems to be present in relatively equal ratios across all the strains and does not show much difference since its the housekeeping σ factor and helps in recognition of majority of promoters in *E.coli*. On the contrary, σ^S shows a radical variation in its amounts across the strains. The most vital finding was the presence of low concentrations of σ^S in crude cell as well as in asscociation with RNAP in the *dksA* mutant in all stages of growth. This is in agreement with the observation that deleting DksA blocks the induction of *rpoS* by ppGpp, since the efficiency of *rpoS* mRNA translation is increased by ppGpp with the help of DksA protein [65]. *RpoS* participates in regulation of almost 50 genes and is also referred to as the *Master regulator of stringent response* in *E.coli* [66]. Moreover, DksA is an RNAP binding transcription factor and directly affects the regulation of stringent genes by working in close association with ppGpp. Consequently, if DksA protein is absent, then this function is impaired leading to an altered function of ppGpp and thereby affecting *rpoS* translation. Additionally, low amounts of σ^S were also observed in *rpoB3449* mutant which can further be investigated upon using oxidative stress experiments with hydrogen peroxide. σ^S is known to regulate oxidative stress in *E.coli* bacteria and therefore, it will be interesting to see how these mutants adapt to such stress conditions with low amounts of σ^S or do other alternative sigma factors and/or NAPs take over the function of σ^S.

Pronounced changes in the competition between σ^{70} and σ^S were also detected in the transition phase of growth in the mutants compared to the wild type. This

tells us that genetic regulation is largely affected due to mutations in the bacterial system. Nonetheless, robustness of the system is such that even after mutations, not only do they try to adapt to the altered conditions, but are able to survive almost as well as the wild type. Furthermore, NAPs come into play and buffer the changes in DNA topology in such a way that RNAP is able to choose between appropriate σ factor and proceeds with the transcription initiation process. This is proved with the detection of varying levels of NAPs in the mutant strains as compared to the wild type through western blot analysis. Higher concentrations of H-NS and IHF proteins were seen in the *rpoB* mutants than the wild type and Δ*dksA*. Both of these are associated with altering the shape of DNA in bacterial systems and involved in introducing negative supercoils in the DNA. This is in agreement with our results of presence of higher amounts of H-NS and IHF in *rpoB* mutants as well as presence of more supercoiled DNA in these mutants throughout the growth when compared to the wild type. Hence, it can be established that DNA topology is constantly regulated by the chromatin remodelling proteins in addition to the σ factors during transcription initiation by RNAP.

Through BN-PAGE and subsequent MALDI-TOF studies about the supramolecular composition of RNAP, it was revealed that several essential metabolic enzymes are bound to the RNAP or present in its close viscinity. This further proves our theory that in *E.coli* bacteria, the metabolic energy is structurally coupled to the transcription machinery i.e. the RNAP enzyme along with the σ factors and NAPs. Also, it has been found that ppGpp levels tend to be low in *rpoB* mutant strains which results in increased ribosome synthesis [67]. These findings are consistent with the results we got. We can clearly see in fig.3.16, that the amounts of ribosomal protein S1 are rather high in *rpoB3370* and *rpoB3449* mutants which may be due to the fact that ppGpp amounts reduce in these mutants and ribosomal genes are transcribed at an increased rate. Also, it may be assumed that some kind of interaction takes place between RNAP and structures of the ribosome and this may be the reason why we see so many ribosomal proteins bound to the RNAP complex in the wild type as well as Δ*dksA* and *rpoB* mutant strains of *E.coli*. This is consistent with one of the studies on ribosomal proteins stating that optimal synthesis of stable RNA is crucially dependent on the co-operation between the RNA polymerase and the ribosome of an appropriate configuration [68]. Moreover, presence of some important metabolic enzymes points to the fact that the chromatin is in constant contact to the metabolic network and any changes in the metabolic energy would directly affect the DNA topology and in turn the RNAP subunit composition and the NAP composition.

In future plans, double mutants like NAP and *rpoB* double mutants could be studied and the effect of these double mutations on the transcription pattern could be analysed. Combinatorial mutations can help us to understand the structural coupling in a more pronounced way and moreover, a global effect could be observed on the genetic expression and transcription profiles in *E.coli* bacteria.

References

[1] Dame, R. T., Espeli, O., Grainger, D. C., and Wiggins, P. A. *Molecular Microbiology* **86**(5), 1023–1030 (2012).

[2] Ebright, R. H. *Journal of Molecular Biology* **304**(5), 687–698 (2000).

[3] Haugen, S. P., Ross, W., and Gourse, R. L. *Nature Reviews Microbiology* **6**(7), 507–519 (2008).

[4] Blatter, E. E., Ross, W., Tang, H., Gourse, R. L., and Ebright, R. H. *Cell* **78**(5), 889–896 (1994).

[5] Paget, M. S. and Helmann, J. D. *Genome Biology* **4**(1), 203 (2003).

[6] Buck, M., Gallegos, M. T., Studholme, D. J., Guo, Y., and Gralla, J. D. *Journal of Bacteriology* **182**(15), 4129–4136 (2000).

[7] Gruber, T. M. and Gross, C. A. *Annual Review of Microbiology* **57**(1), 441–466 (2003).

[8] Lonetto, M., Gribskov, M., and Gross, C. A. *Journal of Bacteriology* **174**(12), 3843–3849 (1992).

[9] Campbell, E. A., Muzzin, O., Chlenov, M., Sun, J. L., Olson, C., Weinman, O., Trester-Zedlitz, M. L., and Darst, S. A. *Molecular Cell* **9**(3), 527–539 (2002).

[10] Heyduk, E., Kuznedelov, K., Severinov, K., and Heyduk, T. *Journal of Biological Chemistry* **281**(18), 12362–12369 (2006).

[11] Schroeder, L. A., Karpen, M. E., and deHaseth, P. L. *Journal of Molecular Biology* **376**(1), 153–165 (2008).

[12] Murakami, K. S., Masuda, S., Campbell, E. A., Muzzin, O., and Darst, S. A. *Science* **296**(5571), 1285–1290 (2002).

[13] Lange, R. and Hengge-Aronis, R. *Molecular Microbiology* **5**(1), 49–59 (1991).

[14] Rahman, M., Hasan, M. R., Oba, T., and Shimizu, K. *Biotechnology and Bioengineering* **94**(3), 585–595 (2006).

[15] Lange, R. and Hengge-Aronis, R. *Genes & Development* **8**(13), 1600–1612 (1994).

[16] Gaal, T., Ross, W., Estrem, S. T., Nguyen, L. H., Burgess, R. R., and Gourse, R. L. *Molecular Microbiology* **42**(4), 939–954 (2001).

[17] Kusano, S., Ding, Q., Fujita, N., and Ishihama, A. *Genes & Development* **271**(4), 1998–2004 (1996).

[18] Colland, F., Barth, M., Hengge-Aronis, R., and Kolb, A. *EMBO Journal* **19**(12), 3028–3037 (2000).

[19] Lange, R., Fischer, D., and Hengge-Aronis, R. *Journal of Bacteriology* **177**(16), 4676–4680 (1995).

[20] Travers, Andrew A.Burgess, R. R. *Nature* **222**(5193), 537–540 (1969).

[21] Mooney, R. A., Darst, S. A., and Landick, R. *Molecular Cell* **20**(3), 335–345 (2005).

[22] Gill, S. C., Weitzel, S. E., and von Hippel, P. H. *Journal of Molecular Biology* **220**(2), 307–324 (1991).

[23] Dillon, S. C. and Dorman, C. J. *Nature Reviews of Microbiology* **8**(3), 185–195 (2010).

[24] Maurer, S., Fritz, J., and Muskhelishvili, G. *Journal of Molecular Biology* **387**(5), 1261–1276 (2009).

[25] Dame, R. T., Luijsterburg, M. S., Krin, E., Bertin, P. N., Wagner, R., and Wuite, G. J. *Journal of Bacteriology* **187**(5), 1845–1848 (2005).

[26] Luijsterburg, M. S., White, M. F., van Driel, R., and Dame, R. T. *Critical Review of Biochemistry and Molecular Biology* **43**(6), 393–418 (2008).

[27] Becker, N. A., Kahn, J. D., and Maher, L. J. *Nucleic Acids Research* **35**(12), 3988–4000 (2007).

[28] Oberto, J., Nabti, S., Jooste, V., Mignot, H., and Rouviere-Yaniv, J. *PLoS ONE* **4**(2), e4367 (2009).

[29] Broyles, S. S. and Pettijohn, D. E. *Journal of Molecular Biology* **187**(1), 47–60 (1986).

[30] Swinger, K. K. and Rice, P. A. *Current Opinion in Structural Biology* **14**(1), 28–35 (2004).

[31] Nash, H. A., Robertson, C. A., Flamm, E., Weisberg, R. A., and Miller, H. I. *Journal of Bacteriology* **169**(9), 4124–4127 (1987).

[32] Santero, E., Hoover, T. R., North, A. K., Berger, D. K., Porter, S. C., and Kustu, S. *Journal of Molecular Biology* **227**(3), 602–620 (1992).

[33] Leonard, A. C. and Grimwade, J. E. *Molecular Microbiology* **55**(4), 978–985 (2005).

[34] Pedersen, A. G., Jensen, L. J., Brunak, S., Staerfeldt, H. H., and Ussery, D. W. *Journal of Molecular Biology* **299**(4), 907–930 (2000).

[35] Schneider, R., Travers, A., Kutateladze, T., and Muskhelishvili, G. *Molecular Microbiology* **34**(5), 953–964 (1999).

[36] Auner, H., Buckle, M., Deufel, A., Kutateladze, T., Lazarus, L., Mavathur, R., Muskhelishvili, G., Pemberton, I., Schneider, R., and Travers, A. *Journal of Molecular Biology* **331**(2), 331–344 (2003).

[37] Weinstein-Fischer, D. and Altuvia, S. *Molecular Microbiology* **63**(4), 1131–1144 (2007).

[38] Rochman, M., Blot, N., Dyachenko, M., Glaser, G., Travers, A., and Muskhelishvili, G. *Molecular Microbiology* **53**(1), 143–152 (2004).

[39] Cashel, M., Gentry, D. R., Hernandez, V. J., and Vinella, D. *American Society for Microbiology Press* , 1458–1496 (1996).

[40] Wendrich, T. M., Blaha, G., Wilson, D. N., Marahiel, M. A., and Nierhaus, K. H. *Molecular Cell* **10**(4), 779–788 (2002).

[41] Zhou, Y. N. and Jin, D. J. *Proceedings of the National Academy of Sciences U.S.A.* **95**(6), 2908–2913 (1998).

[42] Burton, Z. F., Gross, C. A., Watanabe, K. K., and Burgess, R. R. *Cell* **32**(2), 335–349 (1983).

[43] Zhou, Y. N. and Jin, D. J. *Journal of Bacteriology* **179**(13), 4292–4298 (1997).

[44] Marr, C., Geertz, M., Hutt, M. T., and Muskhelishvili, G. *BMC Systems Biology* **2**, 18 (2008).

[45] Sonnenschein, N., Geertz, M., Muskhelishvili, G., and Hutt, M. T. *BMC Systems Biology* **5**, 40 (2011).

[46] Bordes, P., Conter, A., Morales, V., Bouvier, J., Kolb, A., and Gutierrez, C. *Molecular Microbiology* **48**, 561–571 (2003).

[47] Dorman, C. J. *Nat. Rev. Microbiol.* **11**(5), 349–355 (2013).

[48] Swamy, M., Siegers, G. M., Minguet, S., Wollscheid, B., and Schamel, W. W. *Science STKE* **2006**(345), pl4 (2006).

[49] Dyballa, N. and Metzger, S. *Journal of Visualized Experiments* (30) (2009).

[50] Shevchenko, A., Tomas, H., Havlis, J., Olsen, J. V., and Mann, M. *Nature Protocols* **1**(6), 2856–2860 (2006).

[51] Mehandziska, S., (2011).

[52] Parekh, B. S. and Hatfield, G. W. *Proceedings of the National Academy of Sciences U.S.A.* **93**(3), 1173–1177 (1996).

[53] Verma, S., Xiong, Y., Mayer, M. U., and Squier, T. C. *Biochemistry* **46**(11), 3023–3035 (2007).

[54] Loubens, I., Debarbieux, L., Bohin, A., Lacroix, J. M., and Bohin, J. P. *Molecular Microbiology* **10**(2), 329–340 (1993).

[55] Page, F., Altabe, S., Hugouvieux-Cotte-Pattat, N., Lacroix, J. M., Robert-Baudouy, J., and Bohin, J. P. *Journal of Bacteriology* **183**(10), 3134–3141 (2001).

[56] Hedstrom, L. *Chemical Reviews* **109**(7), 2903–2928 (2009).

[57] Allison, A. C. and Eugui, E. M. *Immunopharmacology* **47**(2–3), 85–118 (2000).

[58] Borthwick, A. C., Holms, W. H., and Nimmo, H. G. *Biochemical Journal* **222**(3), 797–804 (1984).

[59] Walsh, K. and Koshland, D. E. *Journal of Biological Chemistry* **260**(14), 8430–8437 (1985).

[60] He, W., Wang, Y., Liu, W., and Zhou, C. Z. *BMC Structural Biology* **7**, 38 (2007).

[61] Polakis, S. E., Guchhait, R. B., and Lane, M. D. *Journal of Biological Chemistry* **248**(22), 7957–7966 (1973).

[62] Guchhait, R. B., Polakis, S. E., Hollis, D., Fenselau, C., and Lane, M. D. *Journal of Biological Chemistry* **249**(20), 6646–6656 (1974).

[63] Bilder, P., Lightle, S., Bainbridge, G., Ohren, J., Finzel, B., Sun, F., Holley, S., Al-Kassim, L., Spessard, C., Melnick, M., Newcomer, M., and Waldrop, G. L. *Biochemistry* **45**(6), 1712–1722 (2006).

[64] Sukhodolets, M. V., Garges, S., and Adhya, S. *RNA* **12**(8), 1505–1513 (2006).

[65] Brown, L., Gentry, D., Elliott, T., and Cashel, M. *Journal of Bacteriology* **184**(16), 4455–4465 (2002).

[66] Becker, G., Klauck, E., and Hengge-Aronis, R. *Molecular Microbiology* **35**(3), 657–666 (2000).

[67] Little, R., Ryals, J., and Bremer, H. *Journal of Bacteriology* **154**(2), 787–792 (1983).

[68] Chakrabarti, S. L. and Gorini, L. *Proceedings of the National Academy of Sciences U.S.A.* **74**(3), 1157–1161 (1977).

Acknowledgment

Bremen, August 19, 2013
